# Transcriptomic analysis of *Eruca vesicaria* subs. *sativa* lines with contrasting tolerance to polyethylene glycol-simulated drought stress

**DOI:** 10.1186/s12870-019-1997-2

**Published:** 2019-10-11

**Authors:** Bang-Lian Huang, Xuan Li, Pei Liu, Lan Ma, Wenhua Wu, Xuekun Zhang, Zaiyun Li, Bangquan Huang

**Affiliations:** 1 Hubei Collaborative Innovation Center for Green Transformation of Bio-Resources, College of Life Science, Hubei University, Wuhan, 430062 China; 2 Jiangsu Microbe Biological & Environmental Engineering Co., Ltd, Wuxi, 214122 China; 3 Key Laboratory of Biology and Genetic Improvement of Oil Crops, Ministry of Agriculture, Oil Crops Research Institute of the Chinese Academy of Agricultural Sciences, Wuhan, 430062 China; 4 National Key Lab of Crop Genetic Improvement, College of Plant Science and Technology, Huazhong Agricultural University, Wuhan, 430070 China

**Keywords:** *Eruca vesicaria* subs. *Sativa*, Genotypes, Drought tolerance, RNA-seq, Gene expression

## Abstract

**Background:**

*Eruca vesicaria subsp. sativa* is one of the Cruciferae species most tolerant to drought stress. In our previous study some extremely drought-tolerant/sensitive *Eruca* lines were obtained. However little is known about the mechanism for drought tolerance in *Eruca*.

**Methods:**

In this study two *E. vesicaria* subs. *sativa* lines with contrasting drought tolerance were treated with liquid MS/PEG solution. Total RNA was isolated from 7-day old whole seedlings and then applied to Illumina sequencing platform for high-throughput transcriptional sequencing.

**Results:**

KEGG pathway analysis indicated that differentially expressed genes (DEGs) involved in alpha-Linolenic acid metabolism, Tyrosine metabolism, Phenylalanine, Tyrosine and tryptophan biosynthesis, Galactose metabolism, Isoquinoline alkaloid biosynthesis, Tropane, Piperidine and pyridine alkaloid biosynthesis, Mineral absorption, were all up-regulated specifically in drought-tolerant (DT) *Eruca* line under drought stress, while DEGs involved in ribosome, ribosome biogenesis, Pyrimidine metabolism, RNA degradation, Glyoxylate and dicarboxylate metabolism, Aminoacyl-tRNA biosynthesis, Citrate cycle, Methane metabolism, Carbon fixation in photosynthetic organisms, were all down-regulated. 51 DEGs were found to be most significantly up-regulated (log_2_ ratio ≥ 8) specifically in the DT line under PEG treatment, including those for ethylene-responsive transcription factors, WRKY and bHLH transcription factors, calmodulin-binding transcription activator, cysteine-rich receptor-like protein kinase, mitogen-activated protein kinase kinase, WD repeat-containing protein, OPDA reductase, allene oxide cyclase, aquaporin, O-acyltransferase WSD1, C-5 sterol desaturase, sugar transporter ERD6-like 12, trehalose-phosphate phosphatase and galactinol synthase 4. Eight of these 51 DEGs wre enriched in 8 COG and 17 KEGG pathways.

**Conclusions:**

DEGs that were found to be most significantly up-regulated specifically in the DT line under PEG treatment, up-regulation of DEGs involved in Arginine and proline metabolism, alpha-linolenic acid metabolism and down-regulation of carbon fixation and protein synthesis might be critical for the drought tolerance in *Eruca*. These results will be valuable for revealing mechanism of drought tolerance in *Eruca* and also for genetic engineering to improve drought tolerance in crops.

**Electronic supplementary material:**

The online version of this article (10.1186/s12870-019-1997-2) contains supplementary material, which is available to authorized users.

## Background

Drought is one of the most important environmental stresses in the world [1–3]. Previous studies indicated that plant responses to drought stress is quite complicated and exploring drought tolerance mechanism in plants is still a challenge [4–7]. Next generation sequencing (NGS) using Illumina HiSeq2000 covers the transcriptome many folds and allows quantification of the detected transcripts [8, 9]. By using next generation sequencing the gene expression is quantified, making it possible for quantitative comparisons [10]. Many researchers have used transcriptome sequencing to study the responses to drought stress in crops [10–16].

Biological and genetic diversity exists among and within plant species regarding drought tolerance, however many of these adaptive mechanisms are not completely understood. Genome-wide identification of drought-responsive transcriptions using genotypes with contrasting drought-tolerance will help revealing interactions among metabolic pathways in response to drought stress [12]. *Eruca vesicaria subsp. sativa* is one of the Cruciferae species most tolerant to drought stress [17, 18]. In our previous study some extremely drought-tolerant/sensitive *Eruca* lines were obtained [19]. In this study, Illumina sequencing technology was used to identify the differentially expressed genes (DEGs) and their biochemical pathways related to drought tolerance in drought tolerant (DT) versus drought-susceptible (DS) *Eruca* genotypes. Expression level of some selected drought-responsive genes was validated by using quantitative RT-PCR. To our knowledge, this is the first report about the whole transcriptome analysis in *E. vesicaria* subsp. *sativa*. The results provide transcriptome data valuable for understanding drought tolerance mechanism in *Eruca* and the differentially expressed genes could be valuable for improving drought tolerance in crops.

## Results

### Overview of Illumina RNA sequencing and de novo assembly of *E. vesicaria* subs. *sativa* transcriptome

In total 314,322,210 raw reads after sequencing produced 254,660,002 clean reads including 430,166 unique genes with N50 value of 638 bp and average length of 511.56 bp. The unigene length ranged from 201 to 16, 517 bp (Table 1).

**Table 1:** Illumina RNA-Seq reads and de novo assembly statistics of *Eruca* transcriptome

Total number of raw reads	314,322,210
Total number of clean reads	254,660,002
Mean length of reads (bp)	90
Number of total unigenes	430,166
Mean length of unigenes (bp)	511.56
Minimum unigene length (bp)	201
Maximum unigene length (bp)	16,517
N50 (bp)	638
Unigenes annotated to Nr	139,090
Unigenes annotated to KEGG	40,463
Unigenes annotated to COG	105,052

The unigenes included 94,463 clusters and 335,703 singletons. Among the unigenes there were 308,310 (71.67%) with length from 200 to 500 bp, 76,255 (17.73%) with length from 500 bp to 1000 bp, 24,662 (5.73%) with length from 1000 bp to 1500 bp, 11,229 (2.61%) with length from 1500 bp to 2000 bp, 9709 (2.26%) with length longer than 2000 bp, and no unigenes shorter than 200 bp (Table 2).

**Table 2:** Length distribution of the Unigenes

Length (nt)	Total	< 200	200–500	500–1000	1000–1500	1500–2000	≥2000
Number of Unigenes	430,166	0	308,310	76,255	24,662	11,229	9709
%	100	0.00	71.67	17.73	5.73	2.61	2.26

**Fig. 1:**
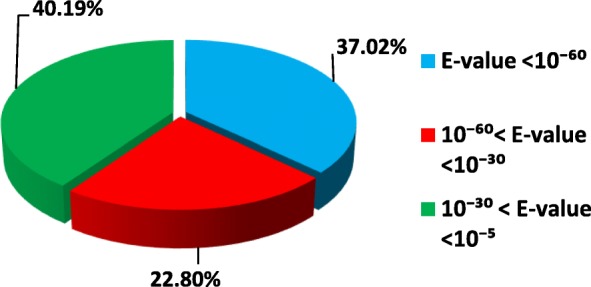
Homology search of unigenes against the Nr database

Based on Nr annotation and the E-value distribution, it was found that among the annotated unigenes 51,486 (37.02%) shared very strong homology (E-value < 10^− 60^), 31,700 (22.80%) had strong homology (10^− 60^ < Evalue < 10^− 30^) and 55, 904 (40.19%) showed homology (10^− 30^ < E-value < 10^− 5^) (Fig. 1). Among the unigenes 83,392 had identity between 22.86–80% and 55,698 higher than 80%.

**Fig. 2:**
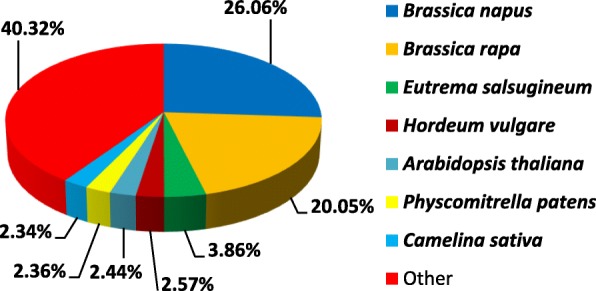
Species distribution of Unigenes in the Nr database

Regarding the species distribution, 83,034 unigene (59.70%) had similarity between 19.32–80%, 56, 056 unigene (40.30%) had similarity higher than 80%. Of the 139,090 unigenes annotated to Nr, 36,254 (26.06%) had matches to sequences from *Brassica napus*, followed by that from *Brassica rapa* (27,894, 20.05%), *Eutrema salsugineum* (5366, 3.86%), *Hordeum vulgare* (3578, 2.57%), *Arabidopsis thaliana* (3391, 2.44%), *Physcomitrella patens* (3289, 2.36%), *Camelina sativa* (3249, 2.34%) (Fig. 2).

### COG annotation and enrichment

**Fig. 3:**
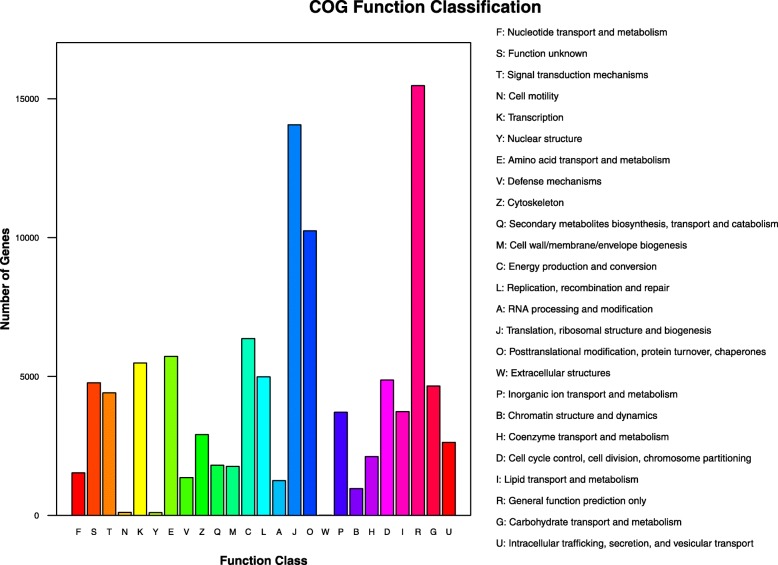
COG Functional classification and distribution

In total 105,052 unigenes were anotated to COG (Clusters of Orthologous Groups of proteins) database and assigned to 25 COG functional clusters (Fig. 3), among which the “general function prediction only” cluster comprised the highest number of unigenes (15,472, 14.73%), followed by the cluster “translation, ribosomal structure and biogenesis” (14,062, 13.39%) and “Posttranslational modification, protein turnover, chaperones” (10,244, 9.75%). By contrast, only 110 unigenes were classified into “Cell motility”, 98 unigenes classified into “Nuclear structure” and 3 unigenes classified into “Extracellular structures” (Fig. 3).

### GO anotation and enrichment

GO (Gene ontology) with Nr annotation indicated that 4953 unigenes were classified into 69 groups that could be categorized into three main classifications: “biological process” (2859), “cellular component” (643) and “molecular function” (1451). In the “molecular function” class the largest number of unigenes (984) were involved in “catalytic activity”; in the “cellular component” class the largest number of unigenes (168) were involved in “cell part”; in the “biological process” class the largest number of unigenes (534) were involved in “metabolic process” (Additional file 1).

### KEGG pathway annotation and unigene enrichment

In total 28,295 unigenes were annotated to 344 KEGG pathways (Additional file 2). The number of unigenes in different KEGG pathways ranged from 1 to 3245. The map with the highest unigene representation was Ribosome (ko03010, 3245 unigenes, 11.47%), followed by Carbon metabolism (ko01200, 1483 unigenes, 5.24%), Biosynthesis of amino acids (ko01230, 1360 unigenes, 4.81%), Protein processing in endoplasmic reticulum (ko04141, 1230 unigenes, 4.35%).

### KEGG pathways and DEGs related to drought tolerance

**Fig. 4:**
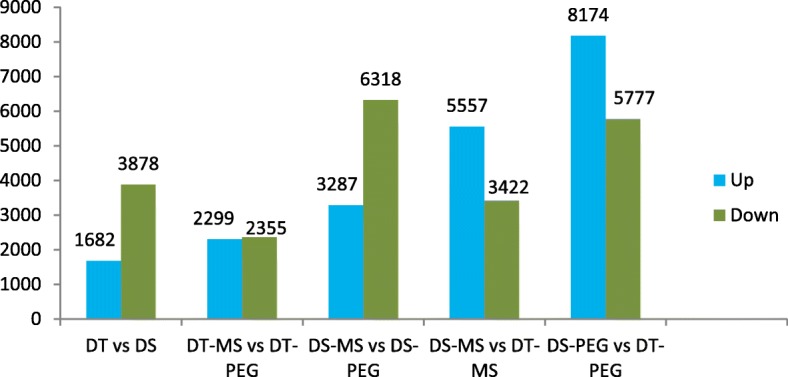
Significantly and differentially expressed genes between drught-tolerant/ sensitive *Eruca* with PEG/MS treatment

Difference in gene expression between drought-tolerant/sensitive *Eruca* lines under MS/PEG treatment was analyzed by mapping back to the previous de novo assembling results using RSEM. As shown in Fig. 4 there were 5560, 4654, 9605, 8979 and 13,951 unigenes showing differential expression including both up- and down-regulated unigenes in DT vs DS, DT-MS vs DT-PEG, DS-MS vs DS-PEG, DS-MS vs DT-MS, DS-PEG vs DT-PEG, respectively.

In the DS-MS vs DS-PEG group, 646 DEGs were enriched in 14 KEGG pathways (Q value < 0.05), i.e., 405 in Ribosome, 32 in Drug metabolism-cytochrome P450, 32 in Plant-pathogen interaction, 26 in Tyrosine metabolism, 40 in Phenylalanine metabolism, 37 in Plant hormone signal transduction, 32 in Metabolism of xenobiotics by cytochrome P450, 30 in Chemical carcinogenesis, 36 in Phenylpropanoid biosynthesis, 47 in Glutathione metabolism, 54 in Lysosome, 7 in D-Glutamine and D-glutamate metabolism, 3 in Biosynthesis of siderophore group nonribosomal peptides and 1 in Atrazine degradation (Additional file 3).

In the DT-MS vs DT-PEG group, 395 DEGs were enriched in 8 KEGG pathways (Q value < 0.05), i.e., 257 in ribosome, 42 in Plant-pathogen interaction, 20 in Phenylalanine metabolism, 31 in Arginine and proline metabolism, 39 in Plant hormone signal transduction, 21 in Phenylpropanoid biosynthesis, 13 in alpha-Linolenic acid metabolism and 41 in D-Glutaminehe D-glutamate metabolism (Additional file 4).

Common pathways betweem DS-MS vs DS-PEG and DT-MS vs DT-PEG are Plant-pathogen interaction, Phenylalanine metabolism, Plant hormone signal transduction, Ribosome, Phenylpropanoid biosynthesis, D-Glutamine and D-glutamate metabolism; Pathways specifically in DS-MS vs DS-PEG are Drug metabolism-cytochrome P450, Atrazine degradation, Tyrosine metabolism, Metabolism of xenobiotics by cytochrome P450, Biosynthesis of siderophore group nonribosomal peptides, Chemical carcinogenesis, Glutathione metabolismand Lysosome; pathways specifically in DT-MS vs DT-PEG are Arginine and proline metabolism and alpha-Linolenic acid metabolism (Additional files 3 and 4).

Referring to Audic’s algorithm [20], FDR (False Discovery Rate) was calculated based on the *P*-value that corresponds to differential expression tests of transcripts. By using “FDR ≤ 0.05 and the absolute value of log_2_ Ratio ≥1” as the threshold, we identified in the DS-MS vs DS-PEG group 3287 unigenes up-regulated and 6318 down-regulated; in the DT-MS vs DT-PEG couple, 2299 unigenes up-regulated and 2355 down-regulated (Fig. 4). It was specifically in DT-MS vs DT-PEG but not in DS-MS vs DS-PEG that 2946 DEGs were detected, among which 1584 were up-regulated and 1362 down-regulated, 449 had Nr annotation and 552 annotated to 232 KEGG pathways (data not shown).

KEGG pathway analysis indicated that in DT-MS vs DT-PEG but not in DS-MS vs DS-PEG, DEGs involved in alpha-Linolenic acid metabolism, Tyrosine metabolism, Phenylalanine, tyrosine and tryptophan biosynthesis, Galactose metabolism, Isoquinoline alkaloid biosynthesis, Tropane, piperidine and pyridine alkaloid biosynthesis, Mineral absorption, were all up-regulated (log_2_ Ratio ≥ 1); DEGs involved in ribosome, ribosome biogenesis, Pyrimidine metabolism, RNA degradation, Glyoxylate and dicarboxylate metabolism, Aminoacyl-tRNA biosynthesis, Citrate cycle, Methane metabolism, Carbon fixation in photosynthetic organisms, were all down-regulated (log_2_ Ratio ≤ − 1). Most DEGs involved in Porphyrin and chlorophyll metabolism, Ubiquinone and other terpenoid-quinone biosynthesis, Arachidonic acid metabolism, Glutathione metabolism, glycerophospholipid metabolism, Phenylalanine metabolism, Plant-pathogen interaction, were up-regulated (log_2_ Ratio ≥ 1); Most DEGs involved in Cell cycle, Peroxisome, Carbon metabolism, protein processing in ER, Regulation of actin cytoskeleton, Arginine and proline metabolism, Pyruvate metabolism, Pentose phosphate pathway, Nitrogen metabolism, Spliceosome, Apoptosis, Lysine degradation, lysosome, calcium signaling pathway, MAPK signaling pathway, Propanoate metabolism, Valine, leucine and isoleucine degradation, Alanine, aspartate and glutamate metabolism, Proteasome, Purine metabolism, RNA transport, Oxidative phosphorylation, down-regulated (Additional file 5).

Among the 2946 DEGs detected specifically in DT-MS vs DT-PEG but not in DS-MS vs DS-PEG, 1890 had blast hits against the NCBI database (Additional file 6), among which 51 DEGs were most significantly up-regulated (log_2_ Ratio ≥ 8), including 3 DEGs for trehalose-phosphate phosphatase, 2 for allene oxide cyclase, 2 for ethylene-responsive transcription factor, 1 for aquaporin TIP1–1, 2 for sugar transporter ERD 6-like 12, 1 for transcription factor MYC3-like, 1 for senescence-associated carboxylesterase 101-like, 1 for calmodulin-binding transcription activator 2, 1 for cysteine-rich receptor-like protein kinase 17, 1 for galactinol synthase 4, 1 for mitogen-activated protein kinase kinase 4, 1 for transcription factor bHLH19-like, 1 for C5-sterol desaturase, 1 for WD repeat-containing protein, 1 for WRKY transcription factor, 1 for O-acyltransferase WSD1, 2 for low-temperature-induced 65 kDa protein; 275 unigenes were most significantly down-regulated (log_2_ Ratio ≤ − 8), including 38 DEGs for ribosomal protein. Fourteen of the 51 most significantly up-regulated DEGs were enriched in 8 COG pathways and 7 in 17 KEGG pathways (Additional file 7 and 8).

### RNA-seq validation: qRT-PCR analysis

**Fig. 5:**
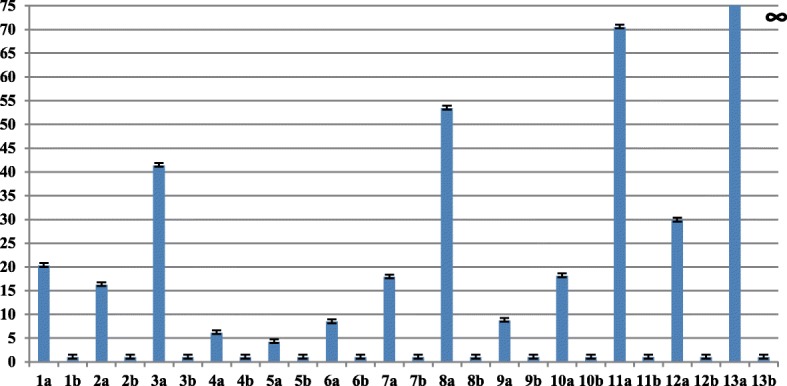
Quantitative RT-PCR confirmation of some up-regulated Unigenes Axis Y represents the relative quantification (RQ) between the PEG and MS treated seedlings, while the error bars indicate standard deviation of three replicates. 1a:c189274_g1_i1-PEG; 1b:c189274_g1_i1-MS; 2a:c205019_g2_i1-PEG; 2b:c205019_g2_i1- MS; 3a:c194336_g1_i1-PEG; 3b:c194336_g1_i1-MS; 4a:c203474_g1_i1-PEG; 4b:c203474_g1_i1-MS; 5a:c194017_g1_i1; 5b:c194017_g1_i1; 6a:c207395_g1_i3-PEG; 6b:c207395_g1_i3-MS; 7a:c175262_g2_i1-PEG; 7b:c175262_g2_i1-MS; 8a:c194457_g2_i2-PEG; 8b:c194457_g2_i2-MS; 9a:c161655_g2_i1-PEG; 9b:c161655_g2_i1-MS; 10a:c197830_g3_i1-PEG; 10b:c197830_g3_i1-MS; 11a:c191638_g3_i8-PEG; 11b:c191638_g3_i8-MS; 12a:c187590_g3_i3-PEG; 12b:c187590_g3_i3-MS; 13a:c200550_g2_i1-PEG; 13b:c200550_g2_i1-MS

**Fig. 6:**
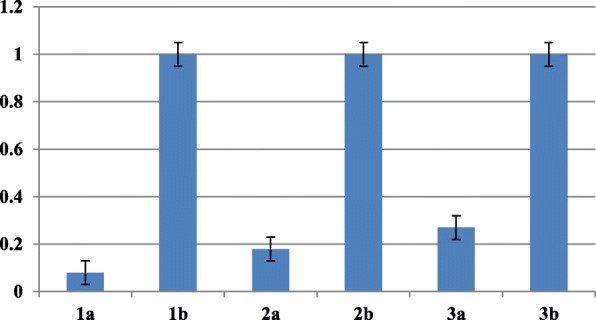
Quantitative RT-PCR confirmation of some down-regulated Unigenes Axis Y represents the relative quantification (RQ) between the PEG and MS treated *Eruca* seedlings, and the error bars indicate standard deviation of three replicates. 1a: proline dehydrogenase (c201592 g1 i1)-PEG; 1b: proline dehydrogenase (c201592 g1 i1)-MS; 2a: NRT1 (c193030 g1 i3)-PEG; 2b: NRT1 (c193030 g1 i3)- MS; 3a: ribosome (c178914 g1 i1)-PEG; 3b: ribosome (c178914 g1 i1) – MS

Quantitative RT-PCR (qRT-PCR) assays were carried out for 16 selected genes to verify the RNA-seq results. ERF4 (c189274 g1 i1), MLO2 (c205019 g2 i1), Ferritin (c194336 g1 i1), receptor-like protein kinase (c203474 g1 i1), dehydrin ERD14 (c194017 g1 i1), disease resistance protein RPP-13 (c207395 g1 i3), O-acyltransferase WSD1-like (c175262_g2_i1), aquaporin TIP1 (c194457_g2_i2), pre-mRNA-splicing factor CLF1 (c161655_g2_i1), potassium transporter (c197830_g3_i1), senescence-associated carboxylesterase (c191638_g3_i8), transcription factor MYC3 (c187590_g3_i3), EARLY FLOWERING 3 (c200550_g2_i1), were found to be up-regulated in DT-MS vs DT-PEG by qRT-PCR; proline dehydrogenase (c201592 g1 i1); NRT1 (c193030 g1 i3), ribosome (c178914 g1 i1), were found to be down-regulated in DT-MS vs DT-PEG by qRT-PCR. The qRT-PCR results are consistent with the RNA-seq results (Fig. 5 and 6).

## Discussion

Comparative study using lines with contrasting drought-tolerance is a useful tool for identifying drought-responsive genes. Identification of DEGs exclusively in the tolerant genotype would be valuable for revealing the mechanisms responsible for stress tolerance [21]. In the present study we provided detailed transcriptomic profiles of whole seedlings of two *Eruca* lines with contrasting drought tolerance.

### ABA responses and stress signaling

Drought stress induces ABA accumulation, which in turn leads to stomatal closure to keep the water status in plants [22–24]. Evidence indicates that *WRKY* proteins, including those induced by ABA, are upregulated under drought stress in rice [25]. *OsWRKY45* overexpression increased drought tolerance [26], while activated expression of *WRKY57* resulted in drought tolerance in *Arabidopsis* [27]. In this study 19 of 26 DEGs for WRKY transcription factor were significantly up-regulated specifically in the drought tolerant *Eruca* line under drought stress (hereafter referred as significantly up-regulated), among which one for WRKY transcription factor 55-like (c195662 g1 i2) was most significantly up-regulated specifically in drought-tolerant *Eruca* line PI 426649 under PEG-simulated drought stress (hereafter referred as most significantly up-regulated).

Basic helix-loop-helix (bHLH) genes are important in phytohormone signaling. Rice OsbHLH148 confers drought tolerance by interacting with OsJAZ proteins [28]. *Arabidopsis* bHLH122 and PebHLH35 from *Populus euphratica* are positive regulators of drought and salt tolerance and osmotic signaling [29, 30]. In this study 5 DEGs for transcription factor bHLH were down-regulated and 8 up-regulated, among which one for transcription factor bHLH19-like (c196938 g3 i6) was most significantly up-regulated (Additional file 6).

### Ethylene-responsive transcription factors

Previous studies have indicated that Ethylene-responsive transcription factors (ERFs) participate in abiotic stress responses in plants [31]. Transcription of *GhERF4* was increased rapidly *Gossypium hirsutum* when plants were exposed to salt stress [32], and overexpression of *ERF* increased drought tolerance [33–36]. In this study 17 of the 18 DEGs for ethylene-responsive transcription factor were up-regulated, among which two for ERF056 (c163592 g1 i1, c163592 g2 i1) were most significantly up-regulated (Additional file 6).

### Antioxidants and ROS modulation

Reactive oxygen species (ROS) often acts as secondary messenger modulating stomatal closure in responses to different stimuli [22, 37]. In *Arabidopsis*, calmodulin-binding transcription activator 1 (AtCAMTA1) is involved in regulation of membrane integrity by inducing ABA responses to drought stress. The *camta1* mutants are highly susceptible to drought stress [38]. In this study 10 DEGs for calmodulin-binding protein were all up-regulated, among which one for calmodulin-binding transcription activator 2-like (c195596 g1 i7) was most significantly up-regulated (Additional file 6).

Mos Cysteine-rich receptor-like kinases (CRKs) are regulated by ROS [39]. It was found that *CRK45* positively regulated plant responses to drought and salt stresses by inducing expression of ABA-responsive and stress-inducible genes. *CRK45* overexpression enhanced drought tolerance in plants [40]. Overexpression of *Arabidopsis CRK5* also increased ABA sensitivity and thus enhanced drought tolerance [41]. In this study 10 of 12 DEGs for cysteine-rich receptor-like protein kinase were up-regulated, among which one for cysteine-rich receptor-like protein kinase 17 (c196393 g1 i2) was most significantly up-regulated (Additional file 6).

It has been shown that MAPKs are involved in plant signal transduction in responses to different environmental stimuli [42–46]. MAPK, MAPKK and MAPKK kinase constitute a functional MAPK cascade. Activation of MAPK helps its translocation to nucleus to phosphorylate and activate transcription factors [47]. AtMKK1 in *Arabidopsis* activates AtMPK3 to transfer abiotic stess signals [48]. NtMEK2 (MAPKK) activates WIPK and SIPK for drought signal transmission [49]. Overexpression of *GhMKK1* increased drought tolerance in *Nicotiana benthamiana* [41]. Overexpression of *AtMKK1* in *Arabidopsis* decreased ROS levels and increased drought tolerance, while *AtMKK1* deficiency resulted in elevated ROS and increased sensitivity to drought tolerance [50]. Plants over expressing *AtMKK4* accumulated fewer ROS and showed less water-loss under drought stress [51]). In this study one DEG for mitogen-activated protein kinase kinase 4 (c198487 g1 i1) was most significantly up-regulated (Additional file 6).

*NAC* genes such as *SNAC3* contributes to drought resistance and osmotic modulation independent of ABA [52]. *SNAC3* could interact with WD domain-containing protein to adjuste ROS in rice. In this study all 13 DEGs for NAC domain-containing protein were up-regulated and one DEG for WD repeat-containing protein 53 (c198319 g1 i2) was most significantly up-regulated (Additional file 6).

### Alpha-linolenic acid, jasmonate signaling and cell membrane stability

Plants keep membrane fluidity and integrity by modulating oleic and linolenic acid levels during stresses [53]. Alpha-linolenic acid is a major precursor for messengers including jasmonic acid generated by oxidative modifications [54–56]. Antisense expression of *Arabidopsis* omega-3 fatty acid desaturase gene led to reduced salt/drought tolerance in tobacco [57], while over-expression of *FAD3* or *FAD8* resulted in increased drought tolerance [58]. Lenka et al. [12] found that eight enzymes involved in alpha-linolenic acid metabolism were significantly induced by drought stress in drought-tolerant rice. In this study we also found all DEGs involved in alpha-linolenic acid metabolism up-regulated (Additional file 5).

Jasmonic acid (JA) plays critical role in stomatal closure during drought stress [59]. It is also involved in the production of antioxidants regulating ascorbate and glutathione metabolism [60]. Based on the pathway established by Vick and Zimmerman [61], JA is produced from alpha-linolenic acid, and in this process allene oxide cyclase and OPDA reductase (OPR) play important roles [62–66]. In this study all four DEGs of OPDA reductase and 6 DEGs for allene oxide cyclase were up-regulated, among which two DEGs for allene oxide cyclase (c184414 g1 i1, c184414 g2 i1) were most significantly up-regulated (Additional file 6).

Previous studies indicated that Aquaporins (AQPs) played an important role in decreasing ion leakage (IL) and malondialdehyde (MDA), thus reducing membrane injury caused by abiotic stresses [67]. Overexpression of banana Aquaporin gene *MaPIP1;1* increased salt tolerance in *Arabidopsis* [68]. In this study one DEG for aquaporin TIP1–1 (c194457 g2 i2) was most significantly up-regulated (Additional file 6).

Cuticular waxes play important roles in reducing non-stomatal water loss under stresses [69]. C-5 sterol desaturase catalyzes the incorporation of C-5 double bond into D7-sterols to form D5, 7-sterols. Overexpression of fungal *C-5 sterol desaturase* increased wax deposition and drought tolerance in tomato [70]. In this study one DEG for C-5 sterol desaturase (c201142 g1 i2) was most significantly up-regulated (Additional file 6).

O-acyltransferase WSD1 is also involved in cuticular wax biosynthesis [71]. Zhang et al. [35] indicated that *KCS* and *WSD*, and their up-stream regulators, were upregulated under drought stress. In this study one DEG for O-acyltransferase WSD1-like (c175262 g2 i1) was most significantly up-regulated (Additional file 6).

### Trehalose, galactinol and sugar transport

In plants various abiotic stresses lead to sugar accumulation in the vacuole [72–74], suggesting that sugar biosynthesis and vacuolar sugar transporters play important roles under these conditions. *Sugar transporter ERD6* expression is induced by drought stress [75], while *ERD6-like transporter* (*ESL1*) expression is enhanced by drought and ABA treatment [76]. In this study 2 DEGs for sugar transporter ERD 6-like 12 (c197650_g1_i3, c197650_g1_i5) were most significantly up-regulated (Additional file 6).

Trehalose is also important in protecting plants from stresses [77]. Trehalose-6-phosphate (T6P) is suggested to act as signaling metabolite responsing to the environment [78]. In plants T6P is dephosphorylated by trehalose-6-phosphate phosphatase to produce trehalose [79]. In this study all 6 DEGs for trehalose-phosphate phosphatase were up-regulated, among which 3 were most-up-regulated (Additional file 6).

Galactinol synthase (GolS) plays important roles in carbon partitioning between sucrose and RFOs and the *GolS* gene products are reported to function in responses to abiotic stresses [80–83]. Transgenic *Arabidopsis* and *Brachypodium distachyon* overexpressing *AtGolS2* accumulated more galactinol and raffinose and showed enhanced drought tolerance [84, 85]. In this study one unigene for galactinol synthase 4 (c194151 g2 i3) was most significantly up-regulated (Additional file 6).

### Down-regulation of carbon fixation and protein synthesis

Lenka et al. [12] found that under drought stress carbon fixation was up-regulated, while in this study we found that in the drought-tolerant *Eruca* line DEGs for carbon fixation in photosynthetic organisms were all down-regulated under drought stress (Additional file 5). Ribosomes are places where proteins are synthesized. Dhindsa and Bewley [86] found decline of amino acid incorporation and polysome population in the drought-tolerant moss *Tortula ruralis* under drought stress. In this study KEGG pathway analysis indicated that DEGs involved in ribosome, ribosome biogenesis, Aminoacyl-tRNA biosynthesis were all down-regulated in drought tolerant *Eruca* under PEG-simulated drought stress (Additional file 5); Blast analysis also indicated that 9 of 10 DEGs for translation initiation factor, 8 DEGs of ATP synthase, 6 DEGs for ribosomal RNA gene, 5 of 6 DEGs for eukaryotic translation initiation factor, 118 of 121 DEGs for ribosomal protein, were down regulated, among which 38 DEGs for ribosomal protein were most significantly down-regulated (Additional file 6), suggesting that *Eruca* might also respond to drought stress by down-regulation of protein synthesis and carbon fixation in photosynthetic organisms.

### Other pathways and DEGs possibly related to drought tolerance

In this study it was found that two DEGs for low-temperature-induced 65 kDa protein-like (c183129 g1 i2, c183129 g3 i4), one for lipase ROG1 (c185493 g1 i1), one for disease resistance protein (c192485 g1 i11), one for amino-acid permease BAT1 (c205647 g1 i3), one for ankyrin repeat-containing protein (c201331 g1 i3), one for acyl-activating enzyme 19 (c188918 g1 i1), one for phospholipid hydroperoxide glutathione peroxidase 6 (c195141 g2 i3), one for potassium transporter 9-like (c197830 g3 i1), one for phosphoglycerate mutase-like protein 2 (c190346 g1 i4) were most significantly up-regulated (Additional file 6). Their roles in drought tolerance need to be further explored.

KEGG pathways in which all DEGs were up-regulated, such as Tyrosine metabolism, Phenylalanine, tyrosine and tryptophan biosynthesis, Galactose metabolism, Isoquinoline alkaloid biosynthesis, Tropane, piperidine and pyridine alkaloid biosynthesis, Mineral absorption; KEGG pathways in which most DEGs were up-regulated, such as Porphyrin and chlorophyll metabolism, Ubiquinone and other terpenoid-quinone biosynthesis, Arachidonic acid metabolism, Glutathione metabolism, glycerophospholipid metabolism, Phenylalanine metabolism, Plant-pathogen interaction, were up-regulated (Additional file 5). Blast analyses indicated that all 5 DEGs for dehydration-responsive element-binding protein, 9 of 10 DEGs for glutathione S-transferase, 8 of 10 DEGs for transcription factor MYB, 4 of 5 DEGs for calcineurin B-like protein, all 16 DEGs for receptor-like protein kinase, 15 of 17 DEGs for zinc finger CCCH domain-containing protein, all 14 DEGs for F-box protein, were up-regulated (Additional file 6). These up-regulated pathways and DEGs might also play important roles in drought tolerance in *Eruca*.

## Conclusions

Based on the transcriptomic analyses we postulated that the most significantly up-regulated DEGs for Ethylene-responsive transcription factors, WRKY and bHLH transcription factors involved in stress signaling and ABA responses; DEGs for calmodulin-binding transcription activator, cysteine-rich receptor-like protein kinase, mitogen-activated protein kinase kinase and WD repeat-containing protein involved in antioxidants and ROS modulation; DEGs for OPDA reductase and allene oxide cyclase involved in JA production, C-5 sterol desaturase involved in producing D5, 7-sterols, aquaporin involved in decreasing ion leakage and malondialdehyde, O-acyltransferase WSD1 involved in cuticular wax biosynthesis; trehalose-phosphate phosphatase, galactinol synthase 4 and sugar transporter ERD 6-like 12 involved in osmoprotectant production, up-regulation of DEGs involved in alpha-linolenic acid metabolism, and down-regulation of carbon fixation and protein synthesis might be critical for the drought tolerance in *Eruca* line PI 426649. These results might be valuable for revealing mechanism of drought tolerance in *Eruca* and for improving drought tolerance in crops.

## Materials and methods

### Materials

In this work *Eruca vesicaria subsp. sativa* PI 426649 highly tolerant and PI 426652 highly sensitive to PEG-simulated drought stress [19] were used for transcriptomic analysis.

### Methods

#### Tissue sampling, RNA extraction, library preparation and Illumina sequencing

*Eruca* seeds of PI 426649 and PI 426652 originally from the Agricultural Research Service, USDA were germinated on filter paper immersed in liquid MS medium [87] without sugar or organic components. Seven days later the seedlings were treated for 10 h with 20% PEG-6000/liquid MS and then harvested and frozen imediately in liquid nitrogen and then stored at − 80 °C. Drought-tolerant PI 426649 is denoted as ‘DT and Drought-sensitive PI 426652 is denoted as ‘DS’. Treatment with liquid MS medium is denoted as ‘MS’ while treatment with PEG is denoted as ‘PEG’. Four samples (DT-MS, DT-PEG, DS-MS, DS-PEG) each with two biological replicates were taken for the present study. Total RNA was isolated from the whole seedlings that had been stored at − 80 °C by using TRIZOL total RNA extraction reagent (TAKARA) according to the manufacturer’s protocol. RNA integrity was verified by 1.5% Agrose gel electrophoresis and confirmed using a 2100 Bioanalyzer analyzer (Agilent, CA, USA). The mRNA enrichment, RNA fragmentation, the first and second strand cDNA synthesis and purfying, sequencing adaptors ligation and PCR amplification were performed as described [14]. The libraries were applied to Illumina sequencing platform (HiSeq 2000, SanDiego, CA, USA) for high-throughput sequencing using a paired-end read protocol with 100 bp of data collected per run.

#### De novo sequence assembly, clustering and homology search

After sequencing, the raw image data was transformed into sequence data by base calling using CASAVA package provided by Illumina and saved as raw reads of fastq format and then treated with Trimmomatic(v0.30) to get clean reads. Transcriptome de novo assembly was carried out with short reads assembling program-Trinity (r2013-02-25) [88]. Unigenes from each sample’s assembly were taken into further process of sequence splicing and redundancy removing to acquire non-redundant unigenes as long as possible by TGICL [89].

#### Functional annotation

Blastx alignment (E-value < 0.00001) between unigenes and protein databases like Nr (NCBI non-redundant database), KEGG (Kyoto Encyclopedia Of Genes and Genomes, www.genome.jp/kegg/) and COG (Clusters of Orthologous Groups of proteins, http://www.ncbi.nlm.nih.gov/COG/) is performed as described [90]. After Nr annotation, Blast2GO program was used to get GO annotation for the unigenes. WEGO software was used to do GO functional classification for all unigenes and to understand the species distribution of gene functions at the macro level [91]. KEGG pathway annotation was carried out by using bidirectional best hit method (BBH) at KEGG Automatic Annotation Server (KAAS).

#### Analysis of differentially expressed genes (DEGs), GO and KEGG pathway enrichment

Reads of the drought tolerant and sensitive *Eruca* accessions with PEG/MS treatment were mapped back to our de novo assembling results using RSEM [92]. To evaluate the gene expression, the number of unique match reads was calculated and then normalized to FPKM (Fragments per Kilo base of transcript per Million mapped reads) and then used to calculate the unigene expression with restrictive conditions of | log_2_ Ratio | ≥1.0 and FDR ≤ 0.05. GO enrichment analysis of these DEGs was performed using blast2GO with *P*-value ≤1 and pathway enrichment analysis was carried out using Path finder software against the KEGG database with Q-value ≤1.

#### Validation of the differentially expressed genes by qRT-PCR

To confirm the RNA-Seq result some transcript tags were selected for quantitative RT-PCR (qRT-PCR) analysis. The qRT-PCRs were performed in triplicate according to the Bioer (Hangzhou, China) manufacturer instructions and relative gene expression levels were determined by the 2^-ΔΔCT^ method [93]. Primers listed in Additional file 9 for some DEGs were disgned and synthesized by GenScript (Nanjing) Co, Ltd. for qRT-PCR.

## Additional files

Additional file 1: **Table S1** Total GO enrichment. (DOCX 16 kb)

Additional file 2: **Table S2** KEGG Pathways and Genes Enriched. (DOCX 35 kb)

Additional file 3: **Figure S1** DEGs in the DS-MS vs DS-PEG group enriched in 14 KEGG pathways. (DOCX 137 kb)

Additional file 4: **Figure S2** DEGs in the DT-MS vs DT-PEG group enriched in 8 KEGG pathways. (DOCX 111 kb)

Additional file 5: **Table S3** KEGG pathway enrichment for DEGs specifically in DT-MS vs DT-PEG. (DOCX 19 kb)

Additional file 6: **Table S4** Differentially expressed unigenes specifically in DT-MS vs DT-PEG based on BLAST analysis. (DOCX 202 kb)

Additional file 7: **Figure S3** COG enrichment for DEGs most significantly-up regulated specifically in DT-MS vs DT-PEG group. (DOCX 40 kb)

Additional file 8: **Figure S4** KEGG pathway enrichment for DEGs most significantly-up regulated specifically in DT-MS vs DT-PEG group. (DOCX 58 kb)

Additional file 9: **Table S5** Primers for quantitative RT-PCR. (DOCX 16 kb)

## Data Availability

The datasets generated and/or analysed in this study are available from the corresponding author on reasonable request.

## Abbreviations

ABA: Abscisic acid
AQPs: Aquaporins
BBH: Bidirectional best hit method
bHLH: Basic helix-loop-helix
CAMTA: Calmodulin-binding transcription activator
COG: Clusters of Orthologous Groups of proteins
CRKs: Cysteine-rich receptor-like kinases
DEG: Differentially expressed gene
DS: Drought-sensitive
DT: Drought-tolerant
ERD: Early responsive to dehydration
ERF: Ethylene-responsive transcription factors
FDR: False Discovery Rate
FPKM: Fragments per Kilo base of transcript per Million mapped reads
GO: Gene ontology
GolS: Galactinol synthase
IL: Ion leakage
JA: Jasmonic acid
KAAS: KEGG Automatic Annotation Server
KEGG: Kyoto Encyclopedia Of Genes and Genomes
MDA: Malondialdehyde
MS: Murashige-Skoog
NGS: Next generation sequencing
Nr: NCBI non-redundant database
OPDA: 12-oxophytodienoate
PEG: Polyethylene glycol
q-RT-PCR: Quantitative RT-PCR
ROS: Reactive oxygen species
