# Supplementary material for: Transcriptomic analysis of Eruca vesicaria subs. sativa lines with contrasting tolerance to polyethylene glycol-simulated drought stress
Source: BMC Plant Biol. 2019 Oct 11;19:419. doi: 10.1186/s12870-019-1997-2 (PMC6787972; doi:10.1186/s12870-019-1997-2)
Supplement: Supplementary file 1 — Table S1 Total GO enrichment. (DOCX 16 kb) [file 12870_2019_1997_MOESM1_ESM.docx]

| GO | GO Term, | Unigene Number | Type |
| --- | --- | --- | --- |
| GO:0009055 | electron carrier activity | 6 | molecular function |
| GO:0032501 | multicellular organismal process | 5 | biological process |
| GO:0044425 | membrane part | 70 | cellular component |
| GO:0000988 | protein binding transcription factor activity | 5 | molecular function |
| GO:0032502 | developmental process | 187 | biological process |
| GO:0039679 | viral occlusion body | 1 | cellular component |
| GO:0044423 | virion part | 2 | cellular component |
| GO:0045499 | chemorepellent activity | 1 | molecular function |
| GO:0030054 | cell junction | 3 | cellular component |
| GO:0045182 | translation regulator activity | 1 | molecular function |
| GO:0032991 | macromolecular complex | 136 | cellular component |
| GO:0005198 | structural molecule activity | 8 | molecular function |
| GO:0030545 | receptor regulator activity | 1 | molecular function |
| GO:0048511 | rhythmic process | 3 | biological process |
| GO:0044217 | other organism part | 1 | cellular component |
| GO:0007610 | behavior | 1 | biological process |
| GO:0002376 | immune system process | 9 | biological process |
| GO:0042056 | chemoattractant activity | 1 | molecular function |
| GO:0044421 | extracellular region part | 2 | cellular component |
| GO:0031386 | protein tag | 2 | molecular function |
| GO:0044464 | cell part | 168 | cellular component |
| GO:0040007 | growth | 20 | biological process |
| GO:0043226 | organelle | 50 | cellular component |
| GO:0046879 | hormone secretion | 1 | biological process |
| GO:0009987 | cellular process | 451 | biological process |
| GO:0051179 | localization | 85 | biological process |
| GO:0005581 | collagen trimer | 1 | cellular component |
| GO:0065007 | biological regulation | 419 | biological process |
| GO:0022414 | reproductive process | 66 | biological process |
| GO:0005623 | cell | 1 | cellular component |
| GO:0016015 | morphogen activity | 1 | molecular function |
| GO:0045735 | nutrient reservoir activity | 2 | molecular function |
| GO:0001906 | cell killing | 2 | biological process |
| GO:0045202 | synapse | 1 | cellular component |
| GO:0030234 | enzyme regulator activity | 25 | molecular function |
| GO:0019012 | virion | 1 | cellular component |
| GO:0022610 | biological adhesion | 4 | biological process |
| GO:0031012 | extracellular matrix | 2 | cellular component |
| GO:0009295 | nucleoid | 4 | cellular component |
| GO:0044848 | biological phase | 4 | biological process |
| GO:0055044 | symplast | 1 | cellular component |
| GO:0016020 | membrane | 33 | cellular component |
| GO:0071840 | cellular component organization or biogenesis | 96 | biological process |
| GO:0050896, | response to stimulus | 242 | biological process |
| GO:0098743 | cell aggregation | 1 | biological process |
| GO:0044699 | single-organism process | 679 | biological process |
| GO:0060089 | molecular transducer activity | 23 | molecular function |
| GO:0016247 | channel regulator activity | 3 | molecular function |
| GO:0036370 | D-alanyl carrier activity | 1 | molecular function |
| GO:0008152 | metabolic process | 534 | biological process |
| GO:0031974 | membrane-enclosed lumen | 4 | cellular component |
| GO:0003824 | catalytic activity | 984 | molecular function |
| GO:0097423 | mitochondrion-associated adherens complex | 1 | cellular component |
| GO:0000003 | reproduction | 4 | biological process |
| GO:0040011 | locomotion | 4 | biological process |
| GO:0005576 | extracellular region | 3 | cellular component |
| GO:0016530 | metallochaperone activity | 3 | molecular function |
| GO:0044456 | synapse part | 1 | cellular component |
| GO:0005488 | binding | 215 | molecular function |
| GO:0098772 | molecular function regulator | 4 | molecular function |
| GO:0016209 | antioxidant activity | 11 | molecular function |
| GO:0023052 | signaling | 5 | biological process |
| GO:0044420 | extracellular matrix component | 1 | cellular component |
| GO:0005215 | transporter activity | 148 | molecular function |
| GO:0001071 | nucleic acid binding transcription factor activity | 2 | molecular function |
| GO:0051704 | multi-organism process | 37 | biological process |
| GO:0005085 | guanyl-nucleotide exchange factor activity | 4 | molecular function |
| GO:0044422 | organelle part | 155 | cellular component |
| GO:0044215 | other organism | 1 | cellular component |
