# Supplementary material for: Transcriptomic analysis of Eruca vesicaria subs. sativa lines with contrasting tolerance to polyethylene glycol-simulated drought stress
Source: BMC Plant Biol. 2019 Oct 11;19:419. doi: 10.1186/s12870-019-1997-2 (PMC6787972; doi:10.1186/s12870-019-1997-2)
Supplement: Supplementary file 2 — Table S2 KEGG Pathways and Genes Enriched. (DOCX 35 kb) [file 12870_2019_1997_MOESM2_ESM.docx]

| Pathway | Unigene Number | Pathway | Unigene Number | Pathway | Unigenes Number |
| --- | --- | --- | --- | --- | --- |
| ko00010 | 665 | ko00905 | 16 | ko04662 | 163 |
| ko00020 | 557 | ko00906 | 39 | ko04664 | 108 |
| ko00030 | 351 | ko00908 | 33 | ko04666 | 337 |
| ko00040 | 189 | ko00909 | 57 | ko04668 | 83 |
| ko00051 | 283 | ko00910 | 213 | ko04670 | 183 |
| ko00052 | 197 | ko00920 | 184 | ko04710 | 116 |
| ko00053 | 163 | ko00930 | 53 | ko04711 | 16 |
| ko00061 | 103 | ko00940 | 236 | ko04712 | 98 |
| ko00062 | 173 | ko00941 | 25 | ko04713 | 142 |
| ko00071 | 382 | ko00942 | 2 | ko04720 | 142 |
| ko00072 | 97 | ko00944 | 1 | ko04721 | 436 |
| ko00073 | 38 | ko00945 | 49 | ko04722 | 265 |
| ko00100 | 205 | ko00950 | 90 | ko04723 | 159 |
| ko00120 | 38 | ko00960 | 93 | ko04724 | 260 |
| ko00130 | 127 | ko00965 | 14 | ko04725 | 145 |
| ko00140 | 61 | ko00966 | 18 | ko04726 | 156 |
| ko00190 | 963 | ko00970 | 572 | ko04727 | 215 |
| ko00195 | 150 | ko00980 | 318 | ko04728 | 298 |
| ko00196 | 33 | ko00981 | 14 | ko04730 | 150 |
| ko00230 | 1088 | ko00982 | 307 | ko04740 | 53 |
| ko00232 | 33 | ko00983 | 222 | ko04742 | 40 |
| ko00240 | 846 | ko00984 | 2 | ko04744 | 48 |
| ko00250 | 438 | ko01040 | 228 | ko04745 | 49 |
| ko00253 | 13 | ko01051 | 33 | ko04810 | 492 |
| ko00260 | 456 | ko01053 | 10 | ko04910 | 508 |
| ko00270 | 467 | ko01054 | 7 | ko04911 | 58 |
| ko00280 | 457 | ko01055 | 11 | ko04912 | 189 |
| ko00281 | 34 | ko01200 | 1483 | ko04913 | 31 |
| ko00290 | 108 | ko01210 | 405 | ko04914 | 287 |
| ko00300 | 116 | ko01220 | 73 | ko04915 | 214 |
| ko00310 | 269 | ko01230 | 1360 | ko04916 | 145 |
| ko00311 | 7 | ko02010 | 187 | ko04917 | 91 |
| ko00330 | 528 | ko02020 | 270 | ko04918 | 141 |
| ko00340 | 135 | ko02030 | 3 | ko04920 | 173 |
| ko00350 | 234 | ko02040 | 1 | ko04930 | 96 |
| ko00360 | 281 | ko03008 | 721 | ko04932 | 549 |
| ko00361 | 20 | ko03010 | 3245 | ko04940 | 58 |
| ko00362 | 101 | ko03013 | 998 | ko04950 | 1 |
| ko00363 | 12 | ko03015 | 564 | ko04960 | 76 |
| ko00364 | 15 | ko03018 | 551 | ko04961 | 133 |
| ko00380 | 310 | ko03020 | 267 | ko04962 | 173 |
| ko00400 | 179 | ko03022 | 219 | ko04964 | 101 |
| ko00401 | 38 | ko03030 | 311 | ko04966 | 221 |
| ko00410 | 265 | ko03040 | 1116 | ko04970 | 114 |
| ko00430 | 70 | ko03050 | 609 | ko04971 | 94 |
| ko00440 | 61 | ko03060 | 250 | ko04972 | 179 |
| ko00450 | 159 | ko03070 | 60 | ko04973 | 82 |
| ko00460 | 125 | ko03320 | 276 | ko04974 | 70 |
| ko00471 | 41 | ko03410 | 240 | ko04975 | 52 |
| ko00472 | 1 | ko03420 | 369 | ko04976 | 150 |
| ko00473 | 5 | ko03430 | 225 | ko04977 | 21 |
| ko00480 | 535 | ko03440 | 206 | ko04978 | 87 |
| ko00500 | 608 | ko03450 | 64 | ko05010 | 756 |
| ko00510 | 257 | ko03460 | 181 | ko05012 | 672 |
| ko00511 | 110 | ko04010 | 348 | ko05014 | 193 |
| ko00512 | 7 | ko04011 | 133 | ko05016 | 1085 |
| ko00513 | 171 | ko04012 | 121 | ko05020 | 127 |
| ko00514 | 39 | ko04013 | 58 | ko05030 | 46 |
| ko00520 | 575 | ko04014 | 318 | ko05031 | 147 |
| ko00521 | 93 | ko04020 | 277 | ko05032 | 84 |
| ko00523 | 22 | ko04060 | 2 | ko05033 | 21 |
| ko00524 | 18 | ko04062 | 213 | ko05034 | 335 |
| ko00531 | 42 | ko04064 | 94 | ko05100 | 220 |
| ko00532 | 6 | ko04064 | 335 | ko05110 | 372 |
| ko00533 | 6 | ko04070 | 286 | ko05111 | 1 |
| ko00534 | 8 | ko04075 | 365 | ko05120 | 317 |
| ko00540 | 18 | ko04080 | 39 | ko05130 | 191 |
| ko00550 | 16 | ko04110 | 528 | ko05131 | 253 |
| ko00561 | 288 | ko04111 | 534 | ko05132 | 306 |
| ko00562 | 298 | ko04112 | 46 | ko05133 | 135 |
| ko00563 | 91 | ko04113 | 346 | ko05134 | 248 |
| ko00564 | 529 | ko04114 | 480 | ko05140 | 58 |
| ko00565 | 157 | ko04115 | 173 | ko05142 | 171 |
| ko00590 | 137 | ko04120 | 764 | ko05143 | 36 |
| ko00591 | 60 | ko04122 | 89 | ko05144 | 11 |
| ko00592 | 132 | ko04130 | 168 | ko05145 | 168 |
| ko00600 | 224 | ko04140 | 153 | ko05146 | 120 |
| ko00601 | 2 | ko04141 | 1230 | ko05150 | 3 |
| ko00603 | 55 | ko04142 | 648 | ko05152 | 486 |
| ko00604 | 38 | ko04144 | 546 | ko05160 | 212 |
| ko00620 | 561 | ko04145 | 602 | ko05161 | 224 |
| ko00621 | 4 | ko04146 | 637 | ko05162 | 221 |
| ko00622 | 4 | ko04150 | 282 | ko05164 | 333 |
| ko00623 | 25 | ko04151 | 575 | ko05166 | 624 |
| ko00624 | 42 | ko04210 | 155 | ko05168 | 443 |
| ko00625 | 101 | ko04260 | 161 | ko05169 | 443 |
| ko00626 | 42 | ko04270 | 167 | ko05200 | 419 |
| ko00627 | 138 | ko04310 | 331 | ko05202 | 154 |
| ko00630 | 423 | ko04320 | 74 | ko05203 | 649 |
| ko00633 | 11 | ko04330 | 80 | ko05204 | 301 |
| ko00640 | 352 | ko04340 | 68 | ko05205 | 358 |
| ko00642 | 6 | ko04350 | 161 | ko05206 | 271 |
| ko00643 | 52 | ko04360 | 226 | ko05210 | 159 |
| ko00650 | 275 | ko04370 | 186 | ko05211 | 206 |
| ko00660 | 36 | ko04380 | 184 | ko05212 | 141 |
| ko00670 | 148 | ko04390 | 185 | ko05213 | 114 |
| ko00680 | 343 | ko04391 | 169 | ko05214 | 152 |
| ko00710 | 386 | ko04510 | 293 | ko05215 | 202 |
| ko00720 | 344 | ko04512 | 27 | ko05216 | 62 |
| ko00730 | 54 | ko04514 | 8 | ko05217 | 22 |
| ko00740 | 92 | ko04520 | 162 | ko05218 | 107 |
| ko00750 | 72 | ko04530 | 231 | ko05219 | 85 |
| ko00760 | 171 | ko04540 | 168 | ko05220 | 123 |
| ko00770 | 145 | ko04610 | 8 | ko05221 | 88 |
| ko00780 | 64 | ko04612 | 206 | ko05222 | 103 |
| ko00785 | 15 | ko04614 | 37 | ko05223 | 114 |
| ko00790 | 107 | ko04620 | 110 | ko05310 | 4 |
| ko00791 | 1 | ko04621 | 95 | ko05321 | 1 |
| ko00830 | 123 | ko04622 | 61 | ko05322 | 199 |
| ko00860 | 209 | ko04623 | 139 | ko05323 | 277 |
| ko00900 | 227 | ko04626 | 329 | ko05340 | 27 |
| ko00901 | 12 | ko04630 | 42 | ko05410 | 48 |
| ko00902 | 12 | ko04640 | 2 | ko05412 | 20 |
| ko00903 | 84 | ko04650 | 168 | ko05414 | 31 |
| ko00904 | 19 | ko04660 | 174 | ko05416 | 86 |
