# Supplementary figures and images for: Transcriptomic analysis of Eruca vesicaria subs. sativa lines with contrasting tolerance to polyethylene glycol-simulated drought stress
Source: BMC Plant Biol. 2019 Oct 11;19:419. doi: 10.1186/s12870-019-1997-2 (PMC6787972; doi:10.1186/s12870-019-1997-2)

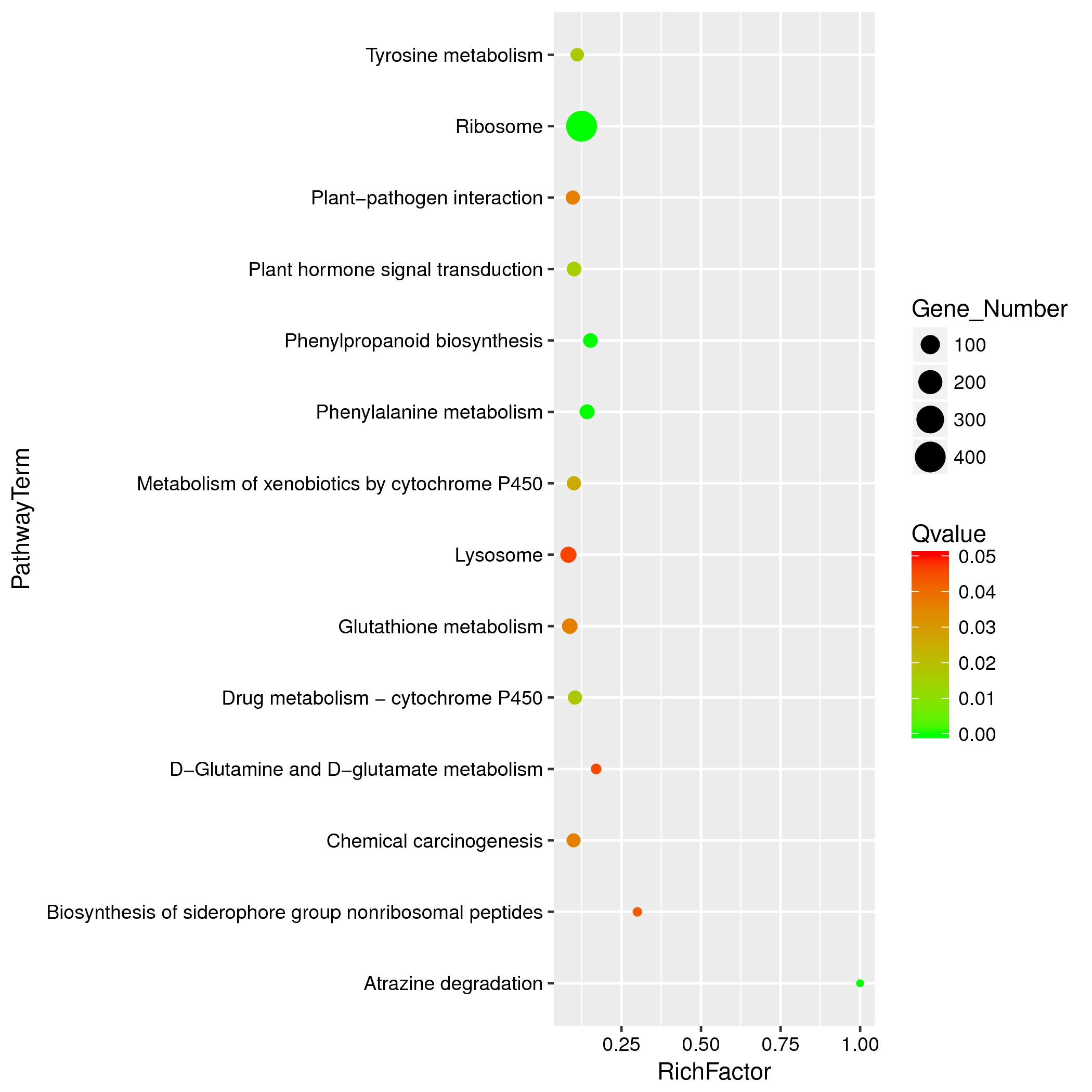

Supplement: Supplementary file 3 — Figure S1 DEGs in the DS-MS vs DS-PEG group enriched in 14 KEGG pathways. (DOCX 137 kb) [file 12870_2019_1997_MOESM3_ESM.docx]

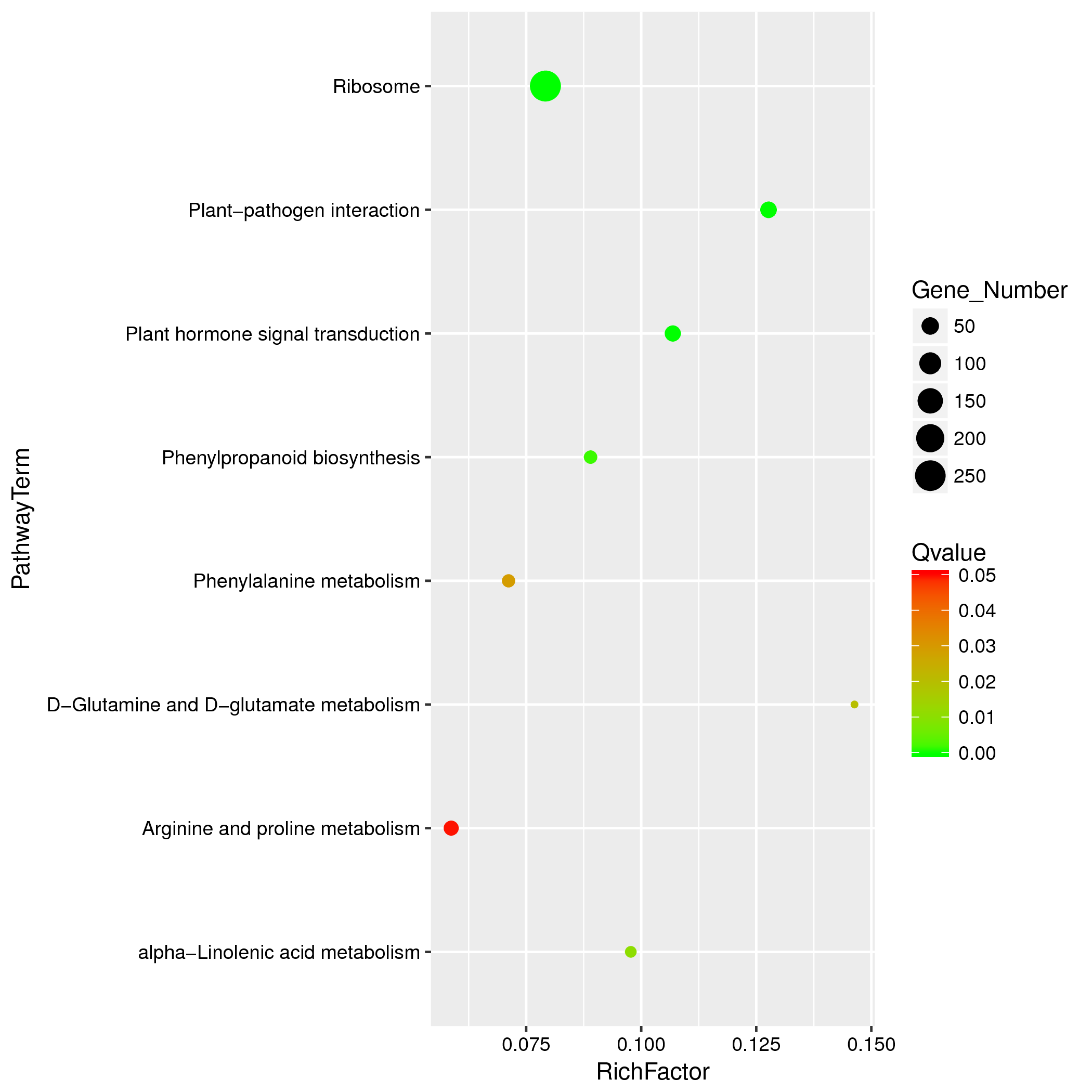

Supplement: Supplementary file 4 — Figure S2 DEGs in the DT-MS vs DT-PEG group enriched in 8 KEGG pathways. (DOCX 111 kb) [file 12870_2019_1997_MOESM4_ESM.docx]

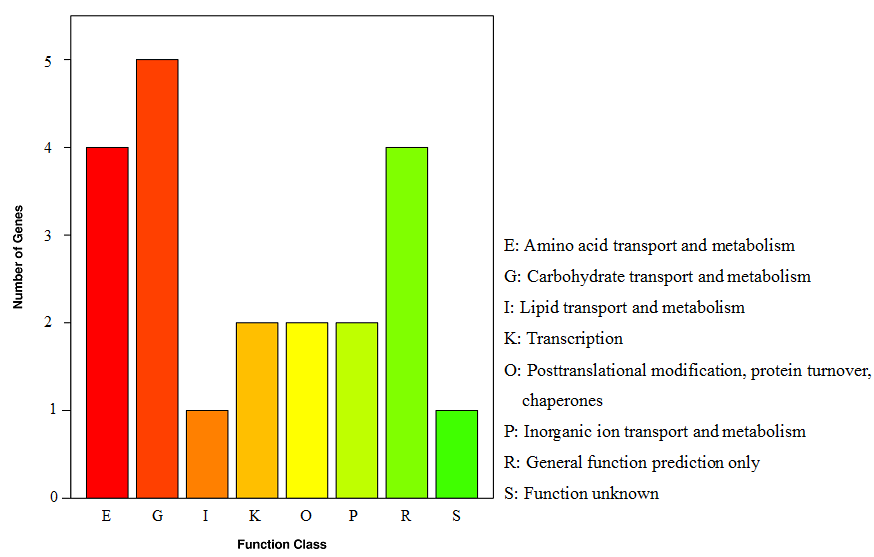

Supplement: Supplementary file 7 — Figure S3 COG enrichment for DEGs most significantly-up regulated specifically in DT-MS vs DT-PEG group. (DOCX 40 kb) [file 12870_2019_1997_MOESM7_ESM.docx]

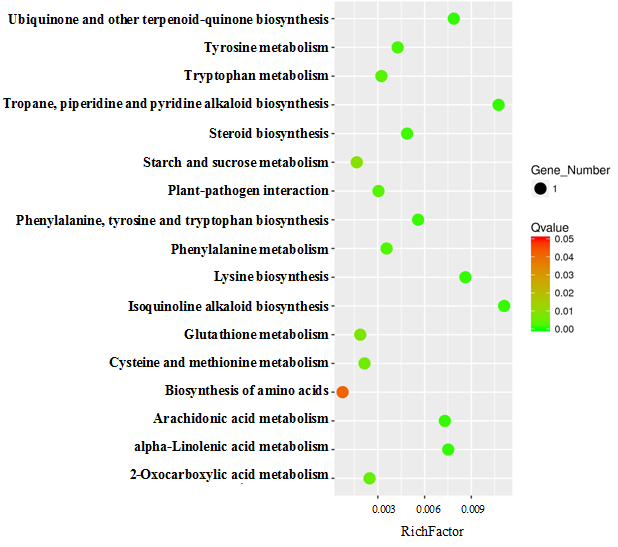

Supplement: Supplementary file 8 — Figure S4 KEGG pathway enrichment for DEGs most significantly-up regulated specifically in DT-MS vs DT-PEG group. (DOCX 58 kb) [file 12870_2019_1997_MOESM8_ESM.docx]
