# Supplementary material for: Transcriptomic analysis of Eruca vesicaria subs. sativa lines with contrasting tolerance to polyethylene glycol-simulated drought stress
Source: BMC Plant Biol. 2019 Oct 11;19:419. doi: 10.1186/s12870-019-1997-2 (PMC6787972; doi:10.1186/s12870-019-1997-2)
Supplement: Supplementary file 5 — Table S3 KEGG pathway enrichment for DEGs specifically in DT-MS vs DT-PEG. (DOCX 19 kb) [file 12870_2019_1997_MOESM5_ESM.docx]

| Pathway description | DEGs | Up-  regulated | Down-  regutated | Up/down ratio |
| --- | --- | --- | --- | --- |
| alpha-Linolenic acid metabolism | 7 | 7 | 0 | 7/0 |
| Tyrosine metabolism | 5 | 5 | 0 | 5/0 |
| Phenylalanine, tyrosine and tryptophan biosynthesis | 4 | 4 | 0 | 4/0 |
| Galactose metabolism | 3 | 3 | 0 | 3/0 |
| Isoquinoline alkaloid biosynthesis | 3 | 3 | 0 | 3/0 |
| Tropane, piperidine and pyridine alkaloid biosynthesis | 3 | 3 | 0 | 3/0 |
| Mineral absorption | 3 | 3 | 0 | 3/0 |
|  |  |  |  |  |
| Drug metabolism - cytochrome P450 | 8 | 7 | 1 | 7.00 |
| Phenylalanine metabolism | 7 | 6 | 1 | 6.00 |
| Plant-pathogen interaction | 26 | 22 | 4 | 5.50 |
| Glutathione metabolism | 12 | 10 | 2 | 5.00 |
| glycerophospholipid metabolism | 11 | 9 | 2 | 4.50 |
| Porphyrin and chlorophyll metabolism | 5 | 4 | 1 | 4.00 |
| Ubiquinone and other terpenoid-quinone biosynthesis | 4 | 3 | 1 | 3.00 |
| Arachidonic acid metabolism | 4 | 3 | 1 | 3.00 |
| plant hormone signal tranduction | 29 | 21 | 8 | 2.63 |
| Starch and sucrose metabolism | 18 | 13 | 5 | 2.60 |
| Metabolism of xenobiotics by cytochrome P450 | 10 | 7 | 3 | 2.33 |
| N-Glycan biosynthesis | 6 | 4 | 2 | 2.00 |
| Cysteine and methionine metabolism | 12 | 8 | 4 | 2.00 |
| Glycine, serine and threonine metabolism | 3 | 2 | 1 | 2.00 |
| Sulfur metabolism | 3 | 2 | 1 | 2.00 |
| Phenylpropanoid biosynthesis | 8 | 5 | 3 | 1.67 |
| Amino sugar and nucleotide sugar metabolism | 9 | 5 | 4 | 1.25 |
| Pentose and glucuronate interconversions | 6 | 3 | 3 | 1.00 |
| Tryptophan metabolism | 6 | 3 | 3 | 1.00 |
| Ascorbate and aldarate metabolism | 4 | 2 | 2 | 1.00 |
| Lysine biosynthesis | 4 | 2 | 2 | 1.00 |
| beta-Alanine metabolism | 4 | 2 | 2 | 1.00 |
| Cyanoamino acid metabolism | 4 | 2 | 2 | 1.00 |
| PPAR signaling pathway | 4 | 2 | 2 | 1.00 |
| Meiosis - yeast | 4 | 2 | 2 | 1.00 |
| Endocytosis | 4 | 2 | 2 | 1.00 |
| glycerolipid metabolism | 7 | 3 | 4 | 0.75 |
| Fc gamma R-mediated phagocytosis | 7 | 3 | 4 | 0.75 |
| Protein export | 5 | 2 | 3 | 0.67 |
| Long-term potentiation | 5 | 2 | 3 | 0.67 |
| Fatty acid degradation | 6 | 2 | 4 | 0.50 |
| Limonene and pinene degradation | 6 | 2 | 4 | 0.50 |
| 2-Oxocarboxylic acid metabolism | 6 | 2 | 4 | 0.50 |
| mRNA surveillance pathway | 6 | 2 | 4 | 0.50 |
| Histidine metabolism | 3 | 1 | 2 | 0.50 |
| Selenocompound metabolism | 3 | 1 | 2 | 0.50 |
| D-Glutamine and D-glutamate metabolism | 3 | 1 | 2 | 0.50 |
| Other glycan degradation | 3 | 1 | 2 | 0.50 |
| Various types of N-glycan biosynthesis | 3 | 1 | 2 | 0.50 |
| Sphingolipid metabolism | 3 | 1 | 2 | 0.50 |
| Butanoate metabolism | 3 | 1 | 2 | 0.50 |
| Glycolysis / Gluconeogenesis | 13 | 4 | 9 | 0.44 |
| ubiquitin-mediated proteolysis | 7 | 2 | 5 | 0.40 |
| Fructose and mannose metabolism | 7 | 2 | 5 | 0.40 |
| Cell cycle | 4 | 1 | 3 | 0.33 |
| Peroxisome | 4 | 1 | 3 | 0.33 |
| Carbon metabolism | 21 | 5 | 16 | 0.31 |
| protein processing in ER | 22 | 5 | 17 | 0.29 |
| Regulation of actin cytoskeleton | 9 | 2 | 7 | 0.29 |
| Arginine and proline metabolism | 14 | 3 | 11 | 0.27 |
| Pyruvate metabolism | 10 | 2 | 8 | 0.25 |
| Pentose phosphate pathway | 5 | 1 | 4 | 0.25 |
| Nitrogen metabolism | 5 | 1 | 4 | 0.25 |
| Spliceosome | 6 | 1 | 5 | 0.20 |
| Apoptosis | 6 | 1 | 5 | 0.20 |
| Lysine degradation | 6 | 1 | 5 | 0.20 |
| lysosome | 13 | 2 | 11 | 0.18 |
| calcium signaling pathway | 7 | 1 | 6 | 0.17 |
| MAPK signaling pathway | 7 | 1 | 6 | 0.17 |
| Propanoate metabolism | 7 | 1 | 6 | 0.17 |
| Valine, leucine and isoleucine degradation | 7 | 1 | 6 | 0.17 |
| Alanine, aspartate and glutamate metabolism | 9 | 1 | 8 | 0.13 |
| Proteasome | 12 | 1 | 11 | 0.09 |
| Purine metabolism | 12 | 1 | 11 | 0.09 |
| RNA transport | 15 | 1 | 14 | 0.07 |
| Oxidative phosphorylation | 17 | 1 | 16 | 0.06 |
| ribosome | 160 | 0 | 160 | 0 |
| ribosome biogenesis | 11 | 0 | 11 | 0 |
|  |  |  |  |  |
| Pyrimidine metabolism | 8 | 0 | 8 | 0 |
| RNA degradation | 7 | 0 | 7 | 0 |
| Glyoxylate and dicarboxylate metabolism | 7 | 0 | 7 | 0 |
| Aminoacyl-tRNA biosynthesis | 6 | 0 | 6 | 0 |
| Citrate cycle | 6 | 0 | 6 | 0 |
|  |  |  |  |  |
|  |  |  |  |  |
|  |  |  |  |  |
| Methane metabolism | 4 | 0 | 4 | 0 |
| Carbon fixation in photosynthetic organisms | 4 | 0 | 4 | 0 |
|  |  |  |  |  |
