# Supplementary material for: Transcriptomic analysis of Eruca vesicaria subs. sativa lines with contrasting tolerance to polyethylene glycol-simulated drought stress
Source: BMC Plant Biol. 2019 Oct 11;19:419. doi: 10.1186/s12870-019-1997-2 (PMC6787972; doi:10.1186/s12870-019-1997-2)
Supplement: Supplementary file 6 — Table S4 Differentially expressed unigenes specifically in DT-MS vs DT-PEG based on BLAST analysis. (DOCX 202 kb) [file 12870_2019_1997_MOESM6_ESM.docx]

| Gene ID | Annotation | log_2_FoldChange | Expression |
| --- | --- | --- | --- |
| c186817_g1_i1 | N-acetyltransferase | 1.3239 | up |
| c202539_g1_i3 | [Fe]-hydrogenase maturation protein HydG | -Inf | Down |
| c180468_g1_i1 | 1,2-dihydroxy-3-keto-5-methylthiopentene dioxygenase 4 | 1.1851 | up |
| c203976_g2_i7 | 125 kDa kinesin-related protein | 2.9575 | up |
| c204607_g1_i3 | 12-oxophytodienoate reductase 1 | 2.1192 | up |
| c193145_g1_i1 | 12-oxophytodienoate reductase 3 | 1.844 | up |
| c193145_g1_i2 | 12-oxophytodienoate reductase 3 | 2.0798 | up |
| c193145_g1_i3 | 12-oxophytodienoate reductase 3 | 3.6814 | up |
| c177549_g1_i1 | 14-3-3 family protein ArtA | -3.2851 | Down |
| c177549_g2_i1 | 14-3-3 protein | -4.5523 | Down |
| c205805_g1_i4 | 14-3-3-like protein GF14 omega | 2.0448 | up |
| c197634_g1_i1 | 14-3-3-like protein GF14 omega | 1.1596 | up |
| c164573_g1_i1 | 1-acyl-sn-glycerol-3-phosphate acyltransferase 2-like | 3.8877 | up |
| c201237_g2_i1 | 1-aminocyclopropane-1-carboxylate oxidase | 1.0593 | up |
| c192830_g1_i1 | 1-aminocyclopropane-1-carboxylate oxidase 4 | 1.5992 | up |
| c187539_g1_i2 | 1-aminocyclopropane-1-carboxylate oxidase-like | 1.8503 | up |
| c187539_g1_i4 | 1-aminocyclopropane-1-carboxylate oxidase-like | 3.0546 | up |
| c201931_g1_i3 | 24-sterol C methyltransferase | -Inf | Down |
| c191234_g1_i1 | 2-isopropylmalate synthase 1 | -1.371 | Down |
| c184983_g1_i1 | 2-oxoglutarate-dependent dioxygenase AOP1 | 1.4508 | up |
| c184983_g1_i3 | 2-oxoglutarate-dependent dioxygenase AOP1 | 1.7381 | up |
| c199018_g2_i1 | 2-oxoglutarate-dependent dioxygenase DAO-like | 1.4707 | up |
| c205828_g2_i5 | 2-oxoglutarateFe(II)-dependent dioxygenase | -3.3624 | Down |
| c261800_g1_i1 | 2-oxoisovalerate dehydrogenase beta subunit | -4.1777 | Down |
| c188518_g2_i1 | 3,9-dihydroxypterocarpan 6A-monooxygenase | -2.1393 | Down |
| c197081_g2_i7 | 3-isopropylmalate dehydratase large subunit-like | -2.383 | Down |
| c203145_g2_i2 | 3-oxoacyl-[acyl-carrier-protein] reductase FabG | 1.5841 | up |
| c3410_g2_i1 | 42464 protein | -Inf | Down |
| c205307_g1_i1 | 4-coumarate--CoA ligase-like 5 | 1.9829 | up |
| c205307_g1_i4 | 4-coumarate--CoA ligase-like 5 | 2.5287 | up |
| c189433_g1_i2 | 5-methyltetrahydrofolate-homocysteine methyltransferase | -5.5343 | Down |
| c162532_g1_i1 | 6-phosphofructo-2-kinasefructose-2,6-biphosphatase 3 | -Inf | Down |
| c194407_g1_i1 | 6-phosphogluconolactonase 3 | 3.5692 | up |
| c150541_g1_i1 | 9-cis-epoxycarotenoid dioxygenase NCED2 | 5.0349 | up |
| c203857_g1_i3 | AAA ATPase domain-containing protein | -Inf | Down |
| c203932_g2_i3 | ABC transporter A family member 7 | 3.2965 | up |
| c197494_g2_i2 | ABC transporter B family member 11 | 1.142 | up |
| c198637_g5_i1 | ABC transporter C family member 14 | 1.6579 | up |
| c198637_g1_i1 | ABC transporter C family member 14 | 1.6833 | up |
| c198637_g4_i1 | ABC transporter C family member 14 | 1.4142 | up |
| c198637_g2_i1 | ABC transporter C family member 14-like | 1.6108 | up |
| c201283_g3_i3 | ABC transporter F family member 3 | -4.0626 | Down |
| c115830_g1_i1 | ABC transporter G family member | -Inf | Down |
| c207037_g2_i1 | ABC transporter G family member 1 | -1.4389 | Down |
| c187070_g2_i1 | ABC transporter G family member 19 | -2.7478 | down |
| c203982_g1_i1 | ABC transporter G family member 22 | 1.116 | up |
| c205678_g1_i2 | ABC transporter G family member 25 | 1.4943 | up |
| c191303_g1_i1 | ABC transporter G family member 42 | -1.0216 | down |
| c199466_g1_i2 | abscisic acid receptor PYL4-like | -1.4742 | Down |
| c199466_g1_i3 | abscisic acid receptor PYL4-like | -1.2805 | Down |
| c192171_g3_i1 | abscisic acid receptor PYL5 | -1.8647 | Down |
| c192171_g3_i2 | abscisic acid receptor PYL5 | -1.5659 | Down |
| c202079_g1_i2 | abscisic acid receptor PYL6-like | -3.0902 | Down |
| c195153_g1_i2 | acetolactate synthase small subunit 1 | 2.5737 | up |
| c204270_g1_i1 | acetyl-CoA acetyltransferase | -1.678 | Down |
| c202272_g1_i2 | acetyltransferase | -4.9213 | Down |
| c202458_g1_i3 | acid beta-fructofuranosidase 3 | 1.7738 | up |
| c168194_g1_i1 | acid beta-fructofuranosidase 4 | 2.1365 | up |
| c196961_g1_i1 | acid beta-fructofuranosidase 4 | 1.1534 | up |
| c198970_g1_i7 | acid phosphatase 1 | 1.3989 | up |
| c203650_g1_i1 | ACT domain repeat 8 protein | 1.6114 | up |
| c203169_g1_i1 | ACT domain-containing protein ACR4 | -1.2646 | Down |
| c197343_g2_i2 | ACT domain-containing protein ACR7-like | 3.1171 | up |
| c197343_g2_i3 | ACT domain-containing protein ACR7-like | 3.1322 | up |
| c197343_g2_i4 | ACT domain-containing protein ACR7-like | 2.3318 | up |
| c163748_g1_i1 | actin binding protein | -6.025 | Down |
| c191807_g3_i4 | actin binding protein, | -5.2237 | Down |
| c159184_g1_i1 | actin related protein 23 complex | -4.3104 | Down |
| c156653_g1_i1 | actin related protein 23 complex subunit 2 | -5.3124 | Down |
| c29988_g1_i1 | actin related protein 23 complex, subunit 4 | -4.6675 | Down |
| c65659_g1_i1 | actin related protein 2-A-like | -Inf | Down |
| c202207_g1_i2 | actin related protein ARP3 | -3.6904 | Down |
| c107514_g1_i1 | actin-depolymerizing factor 2 | 1.5398 | up |
| c138747_g1_i1 | activator inhibitor 1 RNA-binding protein-like | -4.4733 | Down |
| c172659_g1_i1 | activin A receptor, type IB | 2.6478 | up |
| c190997_g1_i1 | acyl carrier protein | -4.011 | down |
| c189528_g1_i1 | acyl-activating enzyme 12, peroxisomal | 3.7708 | up |
| c188918_g1_i1 | acyl-activating enzyme 19 | Inf | up |
| c202970_g2_i1 | adaptor-related protein complex 3, delta 1 subunit | -Inf | down |
| c182706_g2_i2 | adenine nucleotide translocator | -3.875 | Down |
| c157455_g2_i1 | Adenylosuccinate synthetase | -Inf | Down |
| c137886_g2_i1 | adenylyl cyclaseassociated protein | -4.9703 | Down |
| c156395_g2_i1 | ADP,ATP carrier protein | -Inf | Down |
| c197366_g1_i6 | ADPATP translocase 1, | -5.1823 | Down |
| c197366_g1_i4 | ADPATP translocase 3 | -Inf | Down |
| c181784_g1_i6 | ADP-ribosylation factor | -4.9953 | Down |
| c194844_g1_i4 | ADP-ribosylation factor 2-B | 4.7694 | up |
| c115624_g1_i1 | afer afer dicer 1, ribonuclease type III | -Inf | Down |
| c202197_g1_i2 | agmatine deiminase | 1.7846 | up |
| c184365_g1_i1 | Aha1 domain-containing protein | -5.3289 | Down |
| c203785_g1_i2 | ALA-interacting subunit 1 | 1.042 | up |
| c179548_g1_i4 | alanine aminotransferase 1 | -2.4864 | Down |
| c200024_g1_i3 | alanine--glyoxylate aminotransferase 2 homolog 1 | -2.0353 | Down |
| c144906_g1_i1 | alanyl-tRNA synthetase | -5.528 | Down |
| c202775_g1_i4 | aldehyde dehydrogenase | -3.8339 | Down |
| c196326_g1_i2 | aldehyde dehydrogenase (ALD1) | -1.7879 | Down |
| c205399_g1_i3 | aldehyde dehydrogenase family 2 member B4 | 1.3049 | up |
| c196690_g1_i5 | aleurain-like protease gene | -6.6776 | Down |
| c194599_g1_i3 | alkalineneutral invertase CINV1-like | 1.4936 | up |
| c184414_g1_i1 | allene oxide cyclase 2 | Inf | up |
| c184414_g1_i2 | allene oxide cyclase 2 | 4.8947 | up |
| c184414_g2_i1 | allene oxide cyclase 2 | Inf | up |
| c199159_g1_i4 | allene oxide cyclase 3 | 3.3734 | up |
| c199159_g1_i2 | allene oxide cyclase 4 | 3.0586 | up |
| c199159_g1_i7 | allene oxide cyclase 4, chloroplastic | 4.7037 | up |
| c159138_g1_i1 | alpha-actinin | -4.0951 | Down |
| c206698_g1_i2 | alpha-actinin | -4.7045 | Down |
| c203706_g1_i2 | alphabeta hydrolase domain-containing protein 17C | 3.1275 | up |
| c205446_g1_i2 | alpha-L-fucosidase 1 | -1.5845 | down |
| c174462_g2_i1 | amino acid-rich with GYF domain-containing protein 2-like | -Inf | Down |
| c205647_g1_i3 | amino-acid permease BAT1 | Inf | up |
| c205459_g1_i1 | aminopeptidase M1 | 1.3716 | up |
| c195979_g1_i4 | amphipathic helix protein Sin3-like 5 | -Inf | down |
| c192034_g1_i12 | anaphase-promoting complex subunit 4 | 4.4794 | up |
| c199821_g3_i4 | anatolicum voucher Moga-11-1 calreticulin | -5.7519 | Down |
| c202033_g2_i3 | ankyrin repeat domain 11 | -Inf | Down |
| c205058_g2_i2 | ankyrin repeat protein SKIP35 | 2.1726 | up |
| c201331_g1_i3 | ankyrin repeat-containing protein | Inf | up |
| c184895_g2_i1 | Anu4_3 actin gene | -Inf | Down |
| c189637_g3_i2 | apo L domain-containing protein | -Inf | Down |
| c178849_g1_i1 | apoptosis-inducing factor 2 | 1.656 | up |
| c205567_g2_i5 | apoptosis-inducing factor 2 | 2.3558 | up |
| c115899_g1_i1 | aquaporin NIP3-1 | -1.175 | Down |
| c187601_g1_i1 | aquaporin PIP2-1 | 1.1068 | up |
| c194457_g2_i2 | aquaporin TIP1-1 | Inf | up |
| c188711_g1_i1 | ARFSAR family small GTPase | -6.2974 | Down |
| c200022_g1_i1 | arginine decarboxylase 2 | 1.3907 | up |
| c200022_g3_i1 | arginine decarboxylase 2 gene | 1.4106 | up |
| c200870_g1_i1 | armadillo repeat-containing protein 6 | Inf | up |
| c202460_g1_i5 | arogenate dehydrataseprephenate dehydratase 1 | 2.2794 | up |
| c195037_g1_i1 | arogenate dehydrataseprephenate dehydratase 2, chloroplastic-like | 2.6451 | up |
| c196950_g1_i2 | aromatic aminotransferase Aro8 | Inf | up |
| c202598_g1_i1 | asparagine synthetase, mRNA | -Inf | Down |
| c146189_g2_i1 | aspartic proteinase A3 | 1.6465 | up |
| c204979_g1_i1 | aspartic proteinase nepenthesin-2 | 1.9235 | up |
| c198155_g1_i1 | aspartic proteinase nepenthesin-2-like | 1.5745 | up |
| c192354_g1_i1 | aspartic proteinase PCS1 | 1.673 | up |
| c174276_g1_i1 | AT-hook motif nuclear-localized protein 24-like | -1.5898 | Down |
| c188201_g1_i1 | ATP synthase beta chain precursor | -3.7601 | Down |
| c186689_g2_i5 | ATP synthase beta subunit | -6.9594 | Down |
| c189531_g1_i1 | ATP synthase epsilon subunit | -7.1191 | Down |
| c157274_g1_i1 | ATP synthase subunit alpha | -Inf | Down |
| c60271_g1_i1 | ATP synthase subunit gamma | -3.7987 | down |
| c204293_g2_i1 | ATPase family, AAA domain containing 2 (Atad2) | -4.2288 | Down |
| c204018_g1_i2 | ATP-dependent Clp protease proteolytic subunit 3 | 1.3031 | up |
| c193978_g1_i4 | ATP-dependent DNA helicase PIF1-like | 3.3477 | up |
| c202070_g1_i2 | ATP-dependent protease | -3.8629 | Down |
| c27009_g1_i1 | ATPsyn-gamma (DyakATPsyn-gamma) | -Inf | Down |
| c204240_g1_i1 | auxin efflux carrier component 4 | -1.0152 | down |
| c187638_g1_i1 | auxin-induced protein X15-like | 2.3428 | up |
| c184997_g1_i1 | auxin-responsive protein SAUR20-like | -2.9957 | Down |
| c204617_g5_i1 | AX4 S-adenosylmethionine decarboxylase (amd1) | -4.4427 | Down |
| c191877_g1_i2 | axial regulator YABBY 1-like | -Inf | Down |
| c183189_g2_i3 | B2 protein | 1.7776 | up |
| c200079_g1_i1 | basic 7S globulin 2 | -2.0493 | Down |
| c178847_g1_i1 | basic helix-loop-helix domain-containing protein KIAA2018 | -Inf | Down |
| c189859_g1_i1 | basic leucine zipper 1 | -1.7363 | down |
| c194747_g1_i1 | basic leucine zipper 25-like | 2.2438 | up |
| c194747_g1_i3 | basic leucine zipper 25-like | 2.0526 | up |
| c194747_g4_i2 | basic leucine zipper 25-like | 1.8198 | up |
| c121006_g1_i1 | basic transcription factor 3 | -3.4185 | Down |
| c200480_g1_i1 | BEN domain containing 5 (BEND5 | -Inf | Down |
| c192178_g1_i5 | beta carbonic anhydrase 3-like | 1.7112 | up |
| c199534_g1_i1 | beta-1,3-galactosyltransferase 19 | 3.3924 | up |
| c199534_g1_i3 | beta-1,3-galactosyltransferase 19 | 4.1453 | up |
| c199534_g1_i6 | beta-1,3-galactosyltransferase 19 | 2.6097 | up |
| c201601_g1_i2 | beta-1,3-galactosyltransferase 7 | 1.0882 | up |
| c184552_g2_i1 | beta-1,4-glucuronyltransferase 1-like | -Inf | Down |
| c198949_g3_i1 | beta-1,4-mannosyl-glycoprotein 4-beta-N-acetylglucosaminyltransferase | 2.4862 | up |
| c199897_g1_i1 | beta-1,4-mannosyl-glycoprotein 4-beta-N-acetylglucosaminyltransferase | 3.0577 | up |
| c186964_g1_i1 | beta-amylase 1 | 2.151 | up |
| c199328_g1_i1 | beta-amylase 3 | 1.5566 | up |
| c204507_g1_i3 | beta-glucosidase 10 | 2.1272 | up |
| c190603_g1_i1 | beta-glucosidase 27 | 1.1106 | up |
| c200673_g1_i2 | beta-glucosidase 40 | 1.2878 | up |
| c176358_g1_i1 | beta-glucosidase 46 | -1.2363 | Down |
| c187654_g2_i1 | beta-tubulin gene | -Inf | Down |
| c204733_g1_i1 | beta-ureidopropionase | 1.0997 | up |
| c188939_g1_i1 | BI1-like protein | 3.0967 | up |
| c196210_g1_i3 | bidirectional sugar transporter SWEET15-like | 4.2722 | up |
| c203935_g1_i1 | bifunctional aspartokinasehomoserine dehydrogenase | 1.249 | up |
| c199592_g3_i2 | bifunctional dihydrofolate reductase-thymidylate synthase 1 | -4.3925 | Down |
| c182750_g2_i1 | bifunctional nuclease 2 | 1.0069 | up |
| c205993_g1_i1 | bifunctional riboflavin biosynthesis protein RIBA 1 | 1.1658 | up |
| c195126_g1_i2 | binding partner of ACD11 1-like | 1.5227 | up |
| c195126_g1_i5 | binding partner of ACD11 1-like | 2.2842 | up |
| c198434_g1_i1 | bison SEC24 family member A | -Inf | Down |
| c200391_g3_i1 | blw gene for bellwether protein | -6.1379 | down |
| c198047_g1_i5 | BOI-related E3 ubiquitin-protein ligase 2 | 2.7261 | up |
| c195681_g2_i1 | BOI-related E3 ubiquitin-protein ligase 3 | -1.2637 | Down |
| c156246_g1_i1 | brain tumor protein-like | -Inf | Down |
| c204098_g1_i6 | bromodomain adjacent to zinc finger domain protein 2B | -Inf | Down |
| c189603_g1_i5 | BTB POZ domain-containing protein | -3.2181 | Down |
| c202717_g1_i3 | BTBPOZ domain-containing protein | -2.1792 | Down |
| c188641_g2_i2 | Btub mRNA for beta-tubulin | -Inf | Down |
| c140684_g1_i2 | bZIP transcription factor 60 | 1.9708 | up |
| c195410_g3_i2 | bZIP transcription factor HacA | -Inf | down |
| c198834_g1_i2 | C2 domain-containing protein | 3.2658 | up |
| c204337_g1_i2 | C2 domain-containing protein | 1.5137 | up |
| c204337_g1_i4 | C2 domain-containing protein | 2.6472 | up |
| c180458_g1_i1 | C2H2 finger domain-containing protein | -3.5381 | Down |
| c206772_g1_i3 | cadmiumzinc-transporting ATPase HMA3 | -Inf | Down |
| c201687_g1_i2 | caffeic acid 3-O-methyltransferase | 1.254 | up |
| c201687_g2_i1 | caffeic acid 3-O-methyltransferase | 1.4846 | up |
| c195778_g1_i1 | caffeoylshikimate esterase-like | 1.9999 | up |
| c195778_g1_i4 | caffeoylshikimate esterase-like | 2.6595 | up |
| c201771_g2_i2 | calcineurin B-like protein 1 | 4.9294 | up |
| c201771_g2_i3 | calcineurin B-like protein 1 | 2.1852 | up |
| c201771_g2_i4 | calcineurin B-like protein 1 | 4.5435 | up |
| c201771_g2_i5 | calcineurin B-like protein 1 | 2.1785 | up |
| c201771_g1_i1 | calcineurin B-like protein 9 | -1.5728 | Down |
| c185256_g1_i1 | calcineurin regulatory subunit B | -4.8391 | Down |
| c197284_g1_i1 | calcium uniporter protein | -4.7431 | Down |
| c190806_g1_i1 | calcium uniporter protein 5 | 2.7443 | up |
| c205722_g1_i4 | calcium uptake protein 1 homolog | 2.0245 | up |
| c191883_g2_i4 | calcium-binding protein | -5.8754 | Down |
| c165495_g1_i1 | calcium-binding protein CML24-like | 1.1348 | up |
| c203375_g1_i2 | calcium-binding protein CML30 | 2.4312 | up |
| c203375_g1_i3 | calcium-binding protein CML30 | 2.279 | up |
| c179490_g1_i1 | calcium-binding protein CML35 | 1.1898 | up |
| c194451_g1_i1 | calcium-binding protein CML40 | 1.4669 | up |
| c193448_g2_i2 | calcium-binding protein CML42-like | -1.7056 | Down |
| c202455_g3_i2 | calcium-binding protein CML49 | 1.031 | up |
| c202455_g3_i4 | calcium-binding protein CML49 | 1.2134 | up |
| c199050_g1_i1 | calcium-binding protein PBP1-like | 1.1903 | up |
| c199050_g1_i2 | calcium-binding protein PBP1-like | 1.133 | up |
| c198400_g1_i5 | calcium-dependent protein kinase 1 | 1.461 | up |
| c205830_g2_i2 | calcium-dependent protein kinase 1 | 1.1848 | up |
| c198400_g1_i9 | calcium-dependent protein kinase 1 | 2.0072 | up |
| c198400_g1_i1 | calcium-dependent protein kinase 2 | 1.6971 | up |
| c197968_g1_i7 | calcium-dependent protein kinase 28-like | 2.5997 | up |
| c197968_g1_i8 | calcium-dependent protein kinase 28-like | 2.8248 | up |
| c205684_g1_i1 | calcium-dependent protein kinase 32 | 1.545 | up |
| c205684_g1_i2 | calcium-dependent protein kinase 32 | 1.8021 | up |
| c187630_g1_i1 | calcium-dependent protein kinase 32-like | 3.0046 | up |
| c196512_g1_i1 | calcium-dependent protein kinase 3-like | 1.3507 | up |
| c202948_g3_i1 | calcium-transporting ATPase 1, plasma membrane-type-like | -Inf | Down |
| c197828_g2_i1 | calcium-transporting ATPase 13 | 1.6004 | up |
| c197828_g3_i1 | calcium-transporting ATPase 13 | 1.9944 | up |
| c197112_g1_i4 | callose synthase 5 | -2.0947 | down |
| c185543_g1_i1 | calmodulin | 1.8688 | up |
| c200890_g2_i2 | calmodulin- binding protein 25 | 2.2727 | up |
| c165539_g1_i1 | calmodulin gene | -Inf | down |
| c195119_g2_i1 | calmodulin-5 | 1.3471 | up |
| c201526_g1_i6 | Calmodulin-binding family protein | 4.1696 | up |
| c201952_g1_i2 | calmodulin-binding protein | 1.671 | up |
| c201952_g1_i5 | calmodulin-binding protein | 1.9823 | up |
| c200890_g2_i3 | calmodulin-binding protein 25 | 1.5859 | up |
| c200890_g2_i5 | calmodulin-binding protein 25 | 1.8787 | up |
| c201365_g1_i4 | calmodulin-binding protein 60 | 2.2149 | up |
| c201365_g1_i2 | calmodulin-binding protein 60 A | 2.5572 | up |
| c198885_g1_i1 | calmodulin-binding protein 60 A-like | 2.8853 | up |
| c198885_g1_i2 | calmodulin-binding protein 60 A-like | 1.8945 | up |
| c198885_g1_i3 | calmodulin-binding protein 60 A-like | 2.5437 | up |
| c198885_g1_i4 | calmodulin-binding protein 60 A-like | 2.6819 | up |
| c203637_g1_i1 | calmodulin-binding receptor-like cytoplasmic kinase 3 | 1.3986 | up |
| c195596_g1_i2 | calmodulin-binding transcription activator 2-like | 2.0545 | up |
| c195596_g1_i7 | calmodulin-binding transcription activator 2-like | Inf | up |
| c185337_g1_i1 | calmodulin-like protein 5 | 1.3386 | up |
| c179275_g1_i2 | calpain like thiol protease | -Inf | Down |
| c355398_g1_i1 | calreticulin (CALR) | -Inf | down |
| c168926_g1_i1 | cAMP responsive element binding protein-like 2 (CREBL2) | -3.1656 | Down |
| c197028_g1_i3 | carboxylesterase 12 | 1.6209 | up |
| c185852_g2_i1 | carboxylesterase 13 | 2.1044 | up |
| c185852_g4_i5 | carboxylesterase 13 | 1.7649 | up |
| c200712_g1_i4 | carboxylesterase 6 | 1.942 | up |
| c200712_g1_i2 | carboxylesterase 6 | 2.2276 | up |
| c200712_g1_i3 | carboxylesterase 6 | 2.3568 | up |
| c200712_g1_i1 | carboxylesterase 6 | 1.8262 | up |
| c138546_g1_i1 | carboxypeptidase | -Inf | Down |
| c157569_g1_i1 | carboxypeptidase Q-like | -Inf | Down |
| c202127_g2_i1 | carboxyphosphonate phosphorylmutase | 1.365 | up |
| c202805_g1_i5 | casein kinase I | 1.224 | up |
| c193920_g5_i2 | casein kinase I epsilon | -5.1457 | Down |
| c192610_g2_i1 | casein kinase I isoform delta-like | 2.0906 | up |
| c196579_g2_i1 | casein kinase I isoform delta-like | 2.2732 | up |
| c138993_g1_i1 | caseinolytic peptidase B protein homolog | 1.0289 | up |
| c186351_g1_i1 | CASP-like protein 4D2 | -1.53 | Down |
| c190858_g1_i3 | cathepsin L1 | -3.6905 | Down |
| c190858_g1_i4 | cathepsin L1 | -Inf | Down |
| c27546_g1_i1 | cathepsin L1 | -4.0036 | Down |
| c197426_g2_i1 | cathepsin L1-like | -9.1015 | Down |
| c205256_g1_i2 | cationcalcium exchanger 2 | 2.4711 | up |
| c205256_g1_i5 | cationcalcium exchanger 2 | 2.4488 | up |
| c203902_g2_i1 | cationic amino acid transporter 1 | -1.9268 | down |
| c191481_g1_i3 | CBF1 | 2.1217 | up |
| c196943_g3_i1 | CBL-interacting serinethreonine-protein | -1.2802 | down |
| c197270_g1_i1 | CBL-interacting serinethreonine-protein kinase 11 | 2.2628 | up |
| c201990_g1_i3 | CBL-interacting serinethreonine-protein kinase 13 | -1.5795 | Down |
| c195386_g1_i1 | CBL-interacting serinethreonine-protein kinase 2 | 5.9437 | up |
| c195386_g2_i3 | CBL-interacting serinethreonine-protein kinase 2-like | 1.6457 | up |
| c203339_g2_i5 | CBL-interacting serinethreonine-protein kinase 9 | 1.3455 | up |
| c176909_g1_i2 | CBS 513.88 protein | 4.8126 | up |
| c158332_g1_i1 | CCAATenhancer binding protein zeta | -Inf | Down |
| c139778_g1_i1 | CDC42 binding protein kinase gamma | -Inf | Down |
| c200050_g1_i7 | CDPK-related kinase 3 | -Inf | up |
| c201141_g2_i6 | CDPK-related kinase 5 | 4.0438 | up |
| c199104_g1_i1 | Cell division cycle ATPase | 1.7536 | up |
| c165414_g1_i2 | cell division-associated protein bimb partial | -3.0844 | Down |
| c194416_g1_i2 | cell number regulator 8-like | 1.0442 | up |
| c187901_g3_i1 | cellulose synthase-like protein D1 | 1.2363 | up |
| c198550_g2_i2 | cellulose synthase-like protein D2 | 3.2122 | up |
| c200735_g1_i2 | cellulose synthase-like protein D3 | 1.1945 | up |
| c200735_g1_i1 | cellulose synthase-like protein D3 | 1.2623 | up |
| c151775_g5_i1 | cGMP-dependent protein kinase 1 | -Inf | Down |
| c187038_g2_i2 | chaperone BCS1 | 1.2713 | up |
| c202114_g1_i3 | chaperone BCS1-B | 1.7973 | up |
| c198388_g1_i1 | chaperone BCS1-like | 1.2297 | up |
| c101337_g1_i1 | Chaperone dnaK | -Inf | down |
| c191888_g2_i1 | chaperone protein ClpB1-like | 2.0221 | up |
| c191888_g3_i1 | chaperone protein ClpB1-like | 2.1435 | up |
| c199837_g2_i1 | chaperone protein dnaJ 10 | 2.0486 | up |
| c195773_g1_i3 | chaperone protein dnaJ 10-like | 1.0687 | up |
| c199837_g2_i2 | chaperone protein dnaJ 10-like | 2.6774 | up |
| c194869_g1_i2 | chaperone protein dnaJ 11, | -1.2107 | down |
| c194869_g1_i6 | chaperone protein dnaJ 11, | -1.6775 | down |
| c199827_g2_i5 | chaperone protein dnaJ 11, chloroplastic-like | -1.8622 | Down |
| c204359_g1_i1 | chaperonin containing TCP1 theta subunit | -5.2621 | Down |
| c200928_g1_i2 | chaperonin cpn60 | -4.7817 | down |
| c93745_g1_i1 | chaperonin epsilon subunit | -Inf | Down |
| c158889_g1_i1 | chitotriosidase-1 | -2.7293 | Down |
| c205886_g2_i2 | chlorophyll a-b binding protein 8 | 1.2727 | up |
| c200514_g1_i3 | choline kinase 1 | 1.9507 | up |
| c200514_g1_i1 | choline kinase 1 | 1.8652 | up |
| c200514_g1_i2 | choline kinase 1 | 2.0188 | up |
| c198795_g4_i3 | choline kinase 3 | 1.85 | up |
| c198795_g4_i4 | choline kinase 3 | 2.4565 | up |
| c204181_g1_i2 | cholineethanolaminephosphotransferase 2 | 1.2308 | up |
| c164755_g2_i1 | cingulin-like protein 1 | -Inf | Down |
| c200807_g1_i2 | cinnamoyl-CoA reductase 1 | 1.9038 | up |
| c187098_g1_i2 | cinnamoyl-CoA reductase 2-2 | 2.3446 | up |
| c165171_g1_i2 | cinnamyl alcohol dehydrogenase 1 | -Inf | Down |
| c205267_g1_i2 | citrate synthetase | -6.3649 | down |
| c205267_g1_i3 | citrate synthetase | -5.7523 | Down |
| c184228_g1_i4 | class V heat shock protein-like | -2.9697 | Down |
| c200171_g1_i4 | classical arabinogalactan protein 2 | 1.9992 | up |
| c201291_g1_i4 | clathrin assembly protein | 1.7631 | up |
| c205605_g1_i1 | clathrin assembly protein | 1.5413 | up |
| c201291_g1_i2 | clathrin assembly protein | 1.9803 | up |
| c201300_g1_i1 | clathrin assembly protein | 2.6697 | up |
| c201558_g1_i3 | CLK4-associating serinearginine rich protein | 3.3847 | up |
| c196892_g1_i2 | clustered mitochondria protein-like | 3.3336 | up |
| c190941_g1_i3 | coatomer protein gamma 2-subunit | -5.2974 | Down |
| c193646_g1_i1 | coatomer protein gamma 2-subunit | -Inf | Down |
| c185243_g1_i2 | cofilin | -5.6188 | down |
| c197193_g1_i3 | coiled-coil domain containing 172 | -1.9276 | Down |
| c195161_g1_i1 | coiled-coil domain-containing protein 94 homolog | 1.4693 | up |
| c204140_g2_i3 | conserved oligomeric Golgi complex subunit 5 | 1.1145 | up |
| c203805_g1_i4 | contactin-associated protein-like 3B | -5.5303 | Down |
| c189246_g1_i1 | copper-transporting ATPase HMA5 | 1.7186 | up |
| c202618_g1_i3 | copper-transporting ATPase HMA5 | 3.1676 | up |
| c205240_g1_i2 | coronin | -Inf | Down |
| c161861_g1_i1 | corporis ciliary dynein heavy chain | -Inf | Down |
| c205465_g1_i4 | cotton fiber expressed protein 2 | 1.715 | up |
| c182539_g1_i1 | CRM domain-containing protein | -3.4263 | Down |
| c203405_g2_i1 | CSC1-like protein | 2.675 | up |
| c202344_g1_i6 | CSC1-like protein | 5.5983 | up |
| c204158_g1_i2 | CSC1-like protein | 1.7306 | up |
| c180567_g2_i1 | cutinase G-box binding protein (gbp) | -3.1468 | Down |
| c204818_g1_i1 | cyclic nucleotide-gated ion channel 13 | 1.0557 | up |
| c200386_g1_i1 | cyclic nucleotide-gated ion channel 1-like | 2.176 | up |
| c63885_g1_i2 | cyclic nucleotide-gated ion channel 20 | 3.483 | up |
| c197843_g1_i3 | cyclin-dependent kinase 12 (CDK12), | -4.2218 | Down |
| c205519_g1_i3 | cyclin-dependent kinase G1 | 1.5198 | up |
| c205519_g1_i2 | cyclin-dependent kinase G1 | 1.3656 | up |
| c199635_g2_i4 | cyclin-dependent kinase inhibitor 4 | 1.3874 | up |
| c197932_g1_i3 | Cys Met metabolism PLP-dependent enzyme | 3.7556 | up |
| c180822_g1_i1 | cysteine-rich and transmembrane domain-containing protein A | 1.4414 | up |
| c180822_g1_i2 | cysteine-rich and transmembrane domain-containing protein A | 1.7431 | up |
| c184078_g1_i1 | cysteine-rich and transmembrane domain-containing protein A-like | 2.2389 | up |
| c150985_g1_i1 | cysteine-rich receptor-like protein kinase 10 | 3.013 | up |
| c185085_g1_i2 | cysteine-rich receptor-like protein kinase 10 | 3.5906 | up |
| c197132_g3_i3 | cysteine-rich receptor-like protein kinase 10 | 3.2613 | up |
| c204410_g1_i1 | cysteine-rich receptor-like protein kinase 10 | 2.5636 | up |
| c190760_g9_i2 | cysteine-rich receptor-like protein kinase 11 | 2.1571 | up |
| c191736_g1_i6 | cysteine-rich receptor-like protein kinase 17 | 2.1943 | up |
| c196393_g1_i1 | cysteine-rich receptor-like protein kinase 17 | 2.2027 | up |
| c196393_g1_i2 | cysteine-rich receptor-like protein kinase 17 | Inf | up |
| c196393_g1_i5 | cysteine-rich receptor-like protein kinase 17 | 2.3017 | up |
| c195426_g2_i1 | cysteine-rich receptor-like protein kinase 29 | -1.4671 | Down |
| c198991_g1_i5 | cysteine-rich receptor-like protein kinase 35 | -1.0364 | Down |
| c194297_g1_i3 | cysteine-rich receptor-like protein kinase 36 | 1.504 | up |
| c181565_g1_i1 | cysteine-rich repeat secretory protein 38-like | -1.8219 | down |
| c197146_g1_i4 | cysteine-rich repeat secretory protein 55-like | -1.6891 | Down |
| c185365_g1_i1 | cystine lyase CORI3-like | 1.4194 | up |
| c160981_g1_i1 | cythera ribosomal protein S18 (RpS18) | -Inf | Down |
| c162580_g1_i1 | cytidinedeoxycytidylate deaminase family protein | -Inf | Down |
| c204439_g1_i6 | cytoadherence linked asexual protein | -4.9612 | Down |
| c197018_g1_i1 | cytochrome b (CYTB) gene | -5.9298 | Down |
| c201522_g1_i1 | cytochrome b5 | -Inf | Down |
| c201522_g1_i2 | cytochrome B5 isoform D-like | -Inf | Down |
| c187159_g2_i2 | cytochrome c oxidase subunit 5C-1 | 1.0256 | up |
| c198615_g1_i1 | cytochrome oxidase subunit 2 | -5.7015 | Down |
| c173166_g1_i1 | cytochrome oxidase subunit I (COI) gene | -Inf | Down |
| c196087_g1_i4 | cytochrome P450 71B11-like | -2.025 | Down |
| c196087_g1_i3 | cytochrome P450 71B13-like | -1.6153 | Down |
| c181497_g2_i1 | cytochrome P450 71B23-like | 2.4373 | up |
| c196165_g1_i1 | cytochrome P450 71B2-like | 6.3403 | up |
| c196165_g3_i3 | cytochrome P450 71B2-like | 3.4679 | up |
| c196165_g5_i1 | cytochrome P450 71B2-like | 3.5776 | up |
| c197152_g1_i3 | cytochrome P450 71B3-like | 1.8761 | up |
| c204846_g4_i1 | cytochrome P450 72A13 | 1.8187 | up |
| c204846_g3_i2 | cytochrome P450 72A15 | 1.2025 | up |
| c204846_g3_i5 | cytochrome P450 72A15 | 1.8347 | up |
| c202919_g1_i3 | cytochrome P450 734A1 | 1.2652 | up |
| c202919_g1_i4 | cytochrome P450 734A1 | 1.3037 | up |
| c204963_g1_i1 | cytochrome P450 89A2 | 2.0564 | up |
| c187777_g1_i1 | cytochrome P450 94C1-like | 3.0163 | up |
| c201512_g1_i1 | cytokinin dehydrogenase 5 | 1.6378 | up |
| c192451_g1_i6 | cytokinin dehydrogenase 7 | -3.5796 | Down |
| c197407_g1_i7 | cytokinin riboside 5'-monophosphate phosphoribohydrolase | 1.8431 | up |
| c197407_g1_i6 | cytokinin riboside 5'-monophosphate phosphoribohydrolase LOG7-like | 3.114 | up |
| c201638_g1_i5 | DEAD-box ATP-dependent RNA helicase 56 | -5.2772 | Down |
| c201638_g1_i2 | DEAD-box ATP-dependent RNA helicase 56-like | -6.3691 | Down |
| c185255_g1_i2 | dead-box helicase protein | -Inf | Down |
| c196771_g1_i1 | DEHYDRATION-INDUCED 19 homolog 2-like | 1.5333 | up |
| c200767_g1_i4 | DEHYDRATION-INDUCED 19 homolog 4 | 1.1384 | up |
| c179430_g1_i1 | dehydration-responsive element-binding protein 1E | 4.0133 | up |
| c195568_g1_i4 | dehydration-responsive element-binding protein 1F | 5.5441 | up |
| c195568_g1_i3 | dehydration-responsive element-binding protein 1F | 5.1599 | up |
| c195568_g1_i7 | dehydration-responsive element-binding protein 1F-like | 3.8354 | up |
| c200934_g1_i1 | dehydration-responsive element-binding protein 2E | 2.4084 | up |
| c175180_g1_i1 | dehydration-responsive protein RD22-like | -1.0484 | Down |
| c194017_g1_i1 | dehydrin ERD14 | 1.9917 | up |
| c140891_g1_i1 | dehydrin ERD14-like | 2.2057 | up |
| c119975_g1_i1 | delta(4)-desaturase, sphingolipid 2 | -4.7967 | Down |
| c191957_g3_i2 | delta-1-pyrroline-5-carboxylate synthase A | 3.9621 | up |
| c192265_g1_i1 | delta-1-pyrroline-5-carboxylate synthase A-like | 4.2904 | up |
| c192265_g2_i1 | delta-1-pyrroline-5-carboxylate synthase A-like | 2.7735 | up |
| c205458_g1_i4 | Delta9 fatty acid desaturase family protein | -5.1457 | Down |
| c110377_g1_i1 | DENNMADD domain containing 4C (Dennd4c), | -5.2569 | Down |
| c194622_g1_i6 | dentin sialophosphoprotein-like | 1.8851 | up |
| c194744_g1_i2 | deoxyhypusine hydroxylase-like protein | -Inf | Down |
| c177111_g1_i1 | desiccation-related protein LEA14 | 2.8479 | up |
| c177111_g1_i2 | desiccation-related protein LEA14 | 3.1771 | up |
| c199071_g1_i1 | deSI-like protein | 1.881 | up |
| c199071_g1_i2 | deSI-like protein | 2.0813 | up |
| c206836_g1_i1 | diacylglycerol kinase 2 | 1.631 | up |
| c203449_g1_i5 | diacylglycerol kinase 5 | 2.3751 | up |
| c185206_g1_i1 | diacylglycerol kinase 5-like | 2.2816 | up |
| c198307_g1_i1 | diphosphomevalonate decarboxylase-like | -4.9748 | Down |
| c198457_g1_i3 | dirigent protein 25 | -Inf | Down |
| c199791_g1_i11 | disease resistance | 4.3217 | up |
| c185047_g1_i1 | disease resistance protein | 2.342 | up |
| c198409_g2_i1 | disease resistance protein | 1.9556 | up |
| c198461_g1_i4 | disease resistance protein | -Inf | Down |
| c142883_g1_i3 | disease resistance protein | -Inf | down |
| c187580_g2_i1 | disease resistance protein | 2.1316 | up |
| c192485_g1_i11 | disease resistance protein | Inf | up |
| c195289_g1_i1 | disease resistance protein | 1.7123 | up |
| c207311_g1_i3 | disease resistance protein | 2.5073 | up |
| c195020_g3_i6 | disease resistance protein | 1.9272 | up |
| c197225_g3_i1 | disease resistance protein | 1.7041 | up |
| c201339_g1_i7 | disease resistance protein | 1.8347 | up |
| c204445_g1_i1 | disease resistance protein | 1.8724 | up |
| c205853_g1_i2 | disease resistance protein | 1.8415 | up |
| c206472_g1_i1 | disease resistance protein | 2.4112 | up |
| c179348_g1_i1 | disease resistance protein | 1.6238 | up |
| c188396_g4_i4 | disease resistance protein | -Inf | Down |
| c200823_g1_i1 | disease resistance protein | 1.0186 | up |
| c61184_g1_i1 | disease resistance protein | -1.963 | Down |
| c199216_g1_i4 | disease resistance protein (RPS5) | 1.1182 | up |
| c205853_g1_i3 | disease resistance protein RML1A | 1.7593 | up |
| c205853_g1_i4 | disease resistance protein RML1A | 1.1833 | up |
| c191552_g1_i2 | disease resistance protein RML1A-like | 2.7674 | up |
| c193342_g1_i1 | disease resistance protein RML1A-like | 1.731 | up |
| c196219_g3_i1 | disease resistance protein RML1A-like | 1.9968 | up |
| c186726_g1_i2 | disease resistance protein RML1A-like | 1.7672 | up |
| c176289_g1_i1 | disease resistance protein RML1B-like | 4.3525 | up |
| c196107_g1_i5 | disease resistance protein RPP5-like | 1.467 | up |
| c197418_g3_i2 | disease resistance protein RPS6-like | -Inf | Down |
| c189558_g1_i1 | disease resistance protein RRS1-like | 1.6659 | up |
| c200930_g2_i4 | disease resistance protein TAO1 | 1.6871 | up |
| c204127_g2_i3 | disease resistance protein TAO1 | 1.1254 | up |
| c195020_g1_i1 | disease resistance protein TAO1-like | 2.1004 | up |
| c207395_g1_i3 | disease resistance RPP-13 | 1.9006 | up |
| c173932_g3_i1 | disease resistance RPP13-like protein 3 | 4.1734 | up |
| c173932_g1_i2 | disease resistance RPP13-like protein 3 | 3.2987 | up |
| c173932_g2_i1 | disease resistance RPP13-like protein 3 | 6.4151 | up |
| c189562_g3_i2 | disease resistance-like protein | 2.9054 | up |
| c204461_g1_i2 | Disease resistance-like protein CSA1 | 2.6951 | up |
| c187207_g1_i1 | disulfide isomerase | -Inf | Down |
| c194472_g2_i2 | DNA ligase 1-like | 2.1462 | up |
| c191699_g3_i3 | DNA repair protein RAD4-like | 4.5902 | up |
| c196515_g1_i3 | DNA-directed RNA polymerase II subunit RPB1-like | 4.0443 | up |
| c178192_g1_i2 | DnaJ (Hsp40) homolog, subfamily C, member 13 (dnajc13) | -Inf | Down |
| c202258_g1_i1 | dolichyl-diphosphooligosaccharide-protein glycosyltransferase | -Inf | Down |
| c198801_g1_i1 | dolichyl-diphosphooligosaccharide--protein glycosyltransferase subunit 1-like | -5.9238 | Down |
| c115989_g1_i1 | domesticus neurotrypsin-like | -3.8176 | Down |
| c165716_g1_i1 | double-stranded RNA-specific editase Adar | -Inf | Down |
| c325432_g1_i1 | DSM 1558 protein | -3.1334 | Down |
| c199001_g1_i2 | dual specificity phosphatase 8 (DUSP8) | -3.9422 | Down |
| c202757_g1_i8 | DUF21 domain-containing protein At4g14240 | 1.4808 | up |
| c165738_g1_i1 | dynamin central region family protein partial | -Inf | Down |
| c201012_g1_i1 | dynamin-related protein 4C | -Inf | down |
| c262480_g1_i1 | dynein light chain 1 | -Inf | Down |
| c191458_g1_i1 | E3 ubiquitin-protein ligase ATL31-like | 1.1667 | up |
| c184533_g1_i1 | E3 ubiquitin-protein ligase BOI-like | -1.4329 | Down |
| c198478_g1_i5 | E3 ubiquitin-protein ligase DTX3 | 2.4505 | up |
| c198478_g1_i7 | E3 ubiquitin-protein ligase DTX3 | 2.7963 | up |
| c174608_g1_i1 | E3 ubiquitin-protein ligase HUWE1 | -2.8283 | Down |
| c200039_g1_i1 | E3 ubiquitin-protein ligase LUL1 | 2.53 | up |
| c200039_g1_i2 | E3 ubiquitin-protein ligase LUL1 | 1.9338 | up |
| c201821_g2_i5 | E3 ubiquitin-protein ligase MARCH8 | 2.3637 | up |
| c182768_g2_i1 | E3 ubiquitin-protein ligase RHA1B-like | 1.9298 | up |
| c197939_g1_i2 | E3 ubiquitin-protein ligase RHF2A-like | 1.2435 | up |
| c196899_g1_i3 | E3 ubiquitin-protein ligase RING1 | 2.4601 | up |
| c190636_g1_i1 | E3 ubiquitin-protein ligase RING1-like | 1.484 | up |
| c195448_g2_i3 | E3 ubiquitin-protein ligase RNF181 | 2.7494 | up |
| c195522_g1_i2 | E3 ubiquitin-protein ligase RNF181 | 1.5138 | up |
| c197405_g1_i1 | E3 ubiquitin-protein ligase XBAT34 | 1.4124 | up |
| c197405_g1_i2 | E3 ubiquitin-protein ligase XBAT34 | 1.4306 | up |
| c200550_g2_i1 | EARLY FLOWERING 3 | Inf | up |
| c191181_g1_i1 | early nodulin-75 | -2.5581 | Down |
| c192448_g1_i1 | early nodulin-like protein 2 | 2.8766 | up |
| c192448_g1_i3 | early nodulin-like protein 2 | 5.1404 | up |
| c192448_g1_i4 | early nodulin-like protein 2 | 2.946 | up |
| c192448_g1_i5 | early nodulin-like protein 2 | 1.449 | up |
| c202552_g1_i2 | ectonucleotide pyrophosphatasephosphodiesterase family member | 1.0136 | up |
| c182202_g1_i2 | EF-hand domain-containing protein | -Inf | Down |
| c200720_g1_i1 | EGF domain-specific O-linked N-acetylglucosamine transferase-like | 1.7801 | up |
| c194983_g1_i3 | electron transfer flavoprotein subunit alpha | -Inf | Down |
| c194983_g1_i2 | electron transfer flavoprotein subunit alpha | -Inf | up |
| c194983_g1_i5 | electron transfer flavoprotein subunit alpha | -Inf | up |
| c198223_g1_i2 | electron transfer flavoprotein-ubiquinone oxidoreductase | -5.6849 | Down |
| c175355_g1_i1 | elicting plant response protein (Epl1) gene | -2.0234 | Down |
| c199642_g1_i1 | elongation of fatty acids protein 3-like | 2.6186 | up |
| c175161_g1_i1 | elongation of very long chain fatty acids protein 4-like | -5.2748 | Down |
| c190486_g1_i1 | embryogenesis-associated protein EMB8 | -Inf | up |
| c201482_g1_i5 | endochitinase PR4 | 1.5407 | up |
| c166247_g1_i1 | endoglucanase 25 | -3.2393 | Down |
| c192930_g1_i1 | endonuclease 1 | 1.8528 | up |
| c188576_g1_i2 | endonuclease 2-like | 1.3183 | up |
| c192279_g1_i2 | endonuclease MutS2 | 1.9456 | up |
| c204603_g1_i1 | endoplasmic reticulum oxidoreductin-1 | 1.2232 | up |
| c199958_g1_i1 | enhanced disease susceptibility 5 | 2.0239 | up |
| c202914_g3_i2 | enolase gene | -Inf | Down |
| c207584_g1_i1 | enoyl-CoA delta isomerase 2 (ECI2) | -Inf | Down |
| c200249_g1_i1 | ent-kaurene oxidase | 1.1876 | up |
| c205107_g2_i2 | epidermis-specific secreted glycoprotein EP1 | -1.0874 | Down |
| c201437_g1_i1 | epithiospecifier modifier | 1.262 | up |
| c182404_g2_i2 | equilibrative nucleotide transporter 4-like | -3.0468 | Down |
| c197604_g1_i1 | ervatamin-B | 2.7616 | up |
| c197604_g1_i4 | ervatamin-B | 2.8166 | up |
| c198700_g1_i1 | ES1 protein homolog, mitochondrial-like | -Inf | Down |
| c205039_g1_i2 | estrogen receptor 2 (ER beta) (ESR2) | -4.7727 | Down |
| c204931_g2_i1 | ethylene response sensor 2 | 2.7186 | up |
| c197216_g2_i1 | ethylene-responsive transcription factor (ERF) | 1.3052 | up |
| c192431_g1_i3 | ethylene-responsive transcription factor 11-like | 1.7231 | up |
| c192825_g1_i1 | ethylene-responsive transcription factor 13 | 7.001 | up |
| c197216_g1_i1 | ethylene-responsive transcription factor 1A-like | 1.77 | up |
| c197216_g1_i11 | ethylene-responsive transcription factor 1A-like | 1.7377 | up |
| c155349_g2_i1 | ethylene-responsive transcription factor 1B-like | 1.9735 | up |
| c155349_g1_i1 | ethylene-responsive transcription factor 1B-like | 1.9784 | up |
| c178779_g1_i1 | ethylene-responsive transcription factor 1-like | 3.2341 | up |
| c197216_g1_i4 | ethylene-responsive transcription factor 2 | 1.8449 | up |
| c197216_g1_i9 | ethylene-responsive transcription factor 2-like | 1.7361 | up |
| c197216_g1_i2 | ethylene-responsive transcription factor 2-like | 1.3361 | up |
| c189274_g1_i1 | ethylene-responsive transcription factor 4 | 2.8949 | up |
| c199826_g1_i5 | ethylene-responsive transcription factor 5-like | 1.3977 | up |
| c163592_g1_i1 | ethylene-responsive transcription factor ERF056 | Inf | up |
| c163592_g2_i1 | ethylene-responsive transcription factor ERF056-like | Inf | up |
| c192174_g1_i8 | ethylene-responsive transcription factor ERF060 | -1.6003 | Down |
| c194494_g4_i3 | ethylene-responsive transcription factor ERF104 | 1.2428 | up |
| c194287_g1_i1 | ethylene-responsive transcription factor ERF112-like | 1.7634 | up |
| c188105_g1_i1 | eukaryotic initiation factor 4E member 2-like protein | -5.2717 | Down |
| c194237_g3_i2 | eukaryotic translation initiation factor 1A | 2.2793 | up |
| c116878_g1_i1 | eukaryotic translation initiation factor 3 | -3.907 | Down |
| c205502_g2_i1 | eukaryotic translation initiation factor 3 | -4.4355 | Down |
| c205593_g1_i2 | eukaryotic translation initiation factor 3 | -4.7669 | Down |
| c202066_g1_i2 | eukaryotic translation initiation factor 3 subunit B-like | -4.475 | Down |
| c183228_g1_i2 | eukaryotic translation initiation factor 4E | -Inf | Down |
| c204816_g1_i1 | exocyst complex component EXO70B1 | 1.3925 | up |
| c205756_g2_i1 | exocyst complex component EXO70B1 | 1.5858 | up |
| c195674_g3_i1 | exocyst complex component EXO70B1-like | 3.8382 | up |
| c193407_g1_i1 | extensin-2-like | 2.5495 | up |
| c204211_g2_i1 | extra-large guanine nucleotide-binding protein 2 | 2.512 | up |
| c205368_g1_i1 | extra-large guanine nucleotide-binding protein 2 | 2.0582 | up |
| c205368_g3_i1 | extra-large guanine nucleotide-binding protein 2 | 2.5455 | up |
| c205368_g3_i3 | extra-large guanine nucleotide-binding protein 2 | 2.1151 | up |
| c190558_g1_i1 | extra-large guanine nucleotide-binding protein 2 | 2.1878 | up |
| c186350_g7_i2 | extra-large guanine nucleotide-binding protein 2-like | 2.0388 | up |
| c122694_g1_i1 | Factin-capping protein subunit beta | -Inf | Down |
| c157400_g1_i1 | FAM10 family protein | -Inf | Down |
| c190298_g1_i3 | fasciclin-like arabinogalactan protein 11 | 3.8256 | up |
| c166937_g1_i1 | fatty acid desaturase 1 (Fads1) | -4.3773 | Down |
| c200568_g1_i13 | FBD-associated F-box protein | 2.2653 | up |
| c190133_g1_i1 | F-box protein | 3.5009 | up |
| c191630_g1_i2 | F-box protein | 2.4701 | up |
| c200358_g1_i1 | F-box protein | 1.4818 | up |
| c200358_g1_i3 | F-box protein | 2.959 | up |
| c205770_g2_i13 | F-box protein | 3.2695 | up |
| c205770_g2_i3 | F-box protein | 3.0255 | up |
| c188469_g2_i3 | F-box protein PP2-B11 | 2.5404 | up |
| c188469_g2_i1 | F-box protein PP2-B11 | 3.2617 | up |
| c190650_g1_i2 | F-box protein PP2-B2 | 3.3954 | up |
| c190650_g1_i4 | F-box protein PP2-B2 | 3.4918 | up |
| c190650_g1_i5 | F-box protein PP2-B2 | 1.2679 | up |
| c200624_g1_i2 | F-box protein SKIP2-like | 1.0322 | up |
| c199088_g1_i1 | F-boxkelch-repeat protein | 1.1317 | up |
| c178858_g1_i1 | F-boxkelch-repeat protein | -1.3824 | Down |
| c203098_g1_i6 | F-boxkelch-repeat protein | -1.4106 | Down |
| c200203_g2_i6 | F-boxLRR-repeat protein | 2.6177 | up |
| c194762_g2_i2 | F-boxLRR-repeat protein 19 | 2.0041 | up |
| c194762_g2_i4 | F-boxLRR-repeat protein 19 | 2.247 | up |
| c180150_g1_i1 | ferritin 1 | 1.4112 | up |
| c180150_g2_i1 | ferritin-1 | 1.2319 | up |
| c194336_g1_i1 | ferritin-3 | 1.0677 | up |
| c194336_g1_i3 | ferritin-3 | 1.3934 | up |
| c194336_g1_i5 | ferritin-3 | 1.602 | up |
| c176909_g1_i3 | FGSC A4 | 4.9422 | up |
| c191131_g2_i2 | fibrillarin | -Inf | Down |
| c121791_g1_i1 | fibroblast growth factor 2 | -Inf | Down |
| c197875_g2_i4 | fibronectin type-like protein | -4.7507 | Down |
| c164497_g3_i1 | filamin-A | -4.4763 | Down |
| c202324_g3_i1 | fimbrin-1 | 1.2015 | up |
| c196515_g1_i6 | flowering-promoting factor 1-like protein 2 | 3.6418 | up |
| c192098_g7_i1 | FMN-linked oxidoreductase | 2.6085 | up |
| c204828_g3_i4 | folate-biopterin transporter 2 | Inf | up |
| c197388_g1_i2 | formin-like protein 4 | 1.606 | up |
| c203631_g1_i3 | fructose-bisphosphate aldolase cytoplasmic isozyme | -Inf | Down |
| c202228_g1_i2 | furo ATP-binding cassette, sub-family A | -Inf | Down |
| c174319_g1_i1 | GA-binding protein subunit beta-1 | -Inf | Down |
| c194151_g2_i3 | galactinol synthase 4 | Inf | up |
| c199280_g1_i3 | galacturonosyltransferase-like 10 | 1.5446 | up |
| c200726_g1_i1 | gamma complex associated protein 5 | -3.4855 | Down |
| c195890_g1_i1 | gamma-aminobutyric acid (GABA) A receptor | 3.119 | up |
| c196301_g1_i1 | ganglioside-induced differentiation-associated protein 2-like | 1.7246 | up |
| c194201_g1_i4 | GATA transcription factor 22 | -1.8793 | Down |
| c196309_g1_i1 | G-box-binding factor 3-like | 1.6879 | up |
| c196309_g2_i2 | G-box-binding factor 3-like | 1.8056 | up |
| c199486_g1_i1 | G-box-binding factor 4 | 1.4955 | up |
| c199486_g1_i2 | G-box-binding factor 4 | 1.8284 | up |
| c151654_g1_i1 | GDP dissociation inhibitor 1 | -4.7068 | down |
| c205773_g1_i2 | GDP dissociation inhibitor alpha | -5.3022 | down |
| c174076_g1_i2 | GDP-L-galactose phosphorylase 2 | 3.791 | up |
| c198453_g1_i4 | GDP-L-galactose phosphorylase 2 | 1.8157 | up |
| c204004_g1_i1 | GDP-ManMan(3)GlcNAc(2)-PP-Dol alpha-1,2-mannosyltransferase | 1.0838 | up |
| c201047_g1_i2 | GDP-mannose 4,6 dehydratase | -Inf | Down |
| c197399_g1_i1 | GDSL esterase lipase | 2.1354 | up |
| c197399_g2_i1 | GDSL esterase lipase | 1.7162 | up |
| c197399_g2_i2 | GDSL esterase lipase | 2.8013 | up |
| c197399_g2_i3 | GDSL esterase lipase | 2.4265 | up |
| c201437_g1_i3 | GDSL esterase lipase ESM1 | 1.4346 | up |
| c201437_g1_i4 | GDSL esterase lipase ESM1 | 1.1471 | up |
| c197176_g1_i2 | GEM-like protein 5 | 2.4262 | up |
| c192520_g1_i3 | geraniol 8-hydroxylase-like | 1.2022 | up |
| c188069_g1_i1 | germin-like protein subfamily 2 member 2 | 2.194 | up |
| c198071_g1_i2 | germin-like protein subfamily T member 2 | 1.6357 | up |
| c184523_g2_i2 | glucan endo-1,3-beta-glucosidase | 1.4549 | up |
| c197598_g1_i2 | glucan endo-1,3-beta-glucosidase 13-like | -Inf | Down |
| c197598_g1_i4 | glucan endo-1,3-beta-glucosidase 13-like | 1.4641 | up |
| c198649_g2_i1 | glucan endo-1,3-beta-glucosidase 14-like | 1.4872 | up |
| c198649_g2_i2 | glucan endo-1,3-beta-glucosidase 14-like | 1.2008 | up |
| c205975_g2_i6 | glucan endo-1,3-beta-glucosidase 7 | 2.2673 | up |
| c194753_g1_i1 | glucose-6-phosphate 1-epimerase | -1.0006 | Down |
| c190930_g1_i2 | glucose-6-phosphatephosphate translocator 2 | 3.1117 | up |
| c190930_g1_i3 | glucose-6-phosphatephosphate translocator 2 | 2.5913 | up |
| c201519_g1_i1 | glucuronosyltransferase PGSIP8 | 2.5303 | up |
| c201519_g1_i4 | glucuronosyltransferase PGSIP8 | 2.9091 | up |
| c177760_g1_i1 | glucuronoxylan 4-O-methyltransferase 3 | 2.2943 | up |
| c196511_g1_i1 | glutamate dehydrogenase | -4.9247 | Down |
| c202025_g2_i1 | glutamate dehydrogenase | -Inf | Down |
| c202025_g2_i5 | glutamate dehydrogenase 1 | -Inf | Down |
| c203129_g2_i2 | glutamate dehydrogenase, mitochondrial | -Inf | Down |
| c200753_g2_i2 | glutamate-rich protein 2 | -4.9622 | Down |
| c205713_g2_i1 | glutamic acid-rich protein | 1.8881 | up |
| c205713_g2_i5 | glutamic acid-rich protein | 1.4057 | up |
| c205713_g2_i7 | glutamic acid-rich protein | 1.6967 | up |
| c172402_g1_i1 | glutamine amidotransferase YLR126C | 1.2671 | up |
| c203540_g1_i1 | glutamyl-tRNA reductase 2 | 3.0404 | up |
| c197037_g2_i2 | glutamyl-tRNA reductase-binding protein | -1.7914 | Down |
| c138658_g1_i1 | glutaredoxin-C9-like | 4.9637 | up |
| c197754_g2_i2 | glutathione gamma-glutamylcysteinyltransferase 1-like | -1.1986 | Down |
| c181046_g1_i1 | glutathione S-transferase | 1.0503 | up |
| c184280_g1_i2 | glutathione S-transferase | 1.7589 | up |
| c196923_g1_i4 | glutathione S-transferase | 1.9393 | up |
| c182356_g2_i1 | glutathione S-transferase family protein | -Inf | Down |
| c171531_g1_i5 | glutathione S-transferase T3-like | 1.8879 | up |
| c171531_g1_i6 | glutathione S-transferase T3-like | 1.8839 | up |
| c195109_g1_i1 | glutathione S-transferase U25-like | 1.505 | up |
| c195109_g1_i2 | glutathione S-transferase U25-like | 1.7557 | up |
| c193028_g1_i1 | glutathione S-transferase U4-like | 2.1727 | up |
| c193028_g1_i3 | glutathione S-transferase U4-like | 1.7219 | up |
| c177135_g3_i1 | glutenin-like protein (Glu-3) | -Inf | Down |
| c177823_g1_i1 | glycerol-3-phosphate dehydrogenase 1 | -Inf | Down |
| c173598_g1_i3 | glycerophosphodiester phosphodiesterase GDPD2 | 1.6157 | up |
| c200238_g2_i1 | glycerophosphodiester phosphodiesterase GDPD5-like | -1.0418 | Down |
| c175613_g1_i1 | glycerophosphoryl diester phosphodiesterase mRNA | 3.547 | up |
| c177180_g1_i1 | glycine dehydrogenase [decarboxylating] protein | -Inf | Down |
| c201563_g3_i2 | glycine-rich cell wall structural protein 1.8 | 1.3493 | up |
| c201563_g3_i3 | glycine-rich cell wall structural protein 1.8 | 2.3764 | up |
| c201563_g3_i5 | glycine-rich cell wall structural protein 1.8 | 1.9601 | up |
| c201529_g1_i2 | glycine-rich protein | Inf | up |
| c170346_g1_i1 | glycine-rich protein A3 | 5.0376 | up |
| c170346_g1_i2 | glycine-rich protein A3 | 3.5012 | up |
| c180991_g1_i1 | glycine-rich protein A3-like | 1.5578 | up |
| c180991_g1_i2 | glycine-rich protein A3-like | 1.5888 | up |
| c191937_g1_i1 | glycine-rich protein A3-like | 2.275 | up |
| c191937_g1_i4 | glycine-rich protein A3-like | 2.4773 | up |
| c137012_g1_i1 | glycomidase (PNG2) gene | -Inf | Down |
| c115235_g2_i1 | golgin subfamily A member 4 | -Inf | Down |
| c199312_g1_i4 | golgin subfamily A member 6-like protein 2 | 5.3991 | up |
| c205236_g1_i1 | GPI-anchored adhesin-like protein PGA55 | 2.3014 | up |
| c196617_g2_i3 | GPI-anchored protein LORELEI-like | 1.6377 | up |
| c191110_g1_i1 | GPN-loop GTPase 3-like | 3.6956 | up |
| c191110_g1_i2 | GPN-loop GTPase 3-like | 2.4719 | up |
| c191110_g1_i5 | GPN-loop GTPase 3-like | 2.4693 | up |
| c162666_g1_i1 | GTP binding protein 4 (GTPBP4) | -Inf | Down |
| c204015_g1_i2 | GTP diphosphokinase RSH2, chloroplastic | 1.3331 | up |
| c151549_g3_i1 | GTPase activating protein | -Inf | Down |
| c203113_g2_i1 | GTP-binding elongation factor Tu | -Inf | Down |
| c144512_g2_i1 | GTP-binding protein YchF | -Inf | Down |
| c199720_g1_i1 | G-type lectin S-receptor-like serine threonine-protein kinase | 1.3397 | up |
| c200483_g1_i4 | G-type lectin S-receptor-like serinethreonine-protein kinase SD1-29 | 1.5318 | up |
| c167995_g1_i1 | guanine nucleotide binding protein (G protein), | -Inf | Down |
| c166726_g1_i1 | guanine nucleotide exchange factor (GEF) 2 | -3.4735 | Down |
| c179393_g1_i7 | guanine nucleotide-binding protein alpha-1 subunit | 1.474 | up |
| c200630_g3_i5 | guanine nucleotide-binding protein subunit beta-like protein | -2.1947 | Down |
| c300789_g1_i1 | Gv29-8 protein | -3.2649 | Down |
| c298265_g1_i1 | Gv29-8 protein | -Inf | Down |
| c201837_g2_i2 | haloacid dehalogenase-like hydrolase domain-containing protein | 1.4 | up |
| c200038_g1_i1 | haloacid dehalogenase-like hydrolase domain-containing protein 3 | 2.1201 | up |
| c141737_g2_i1 | heat shock protein 70 | -Inf | Down |
| c196704_g2_i2 | heat shock protein 70 | -Inf | Down |
| c133347_g1_i1 | heat shock protein 70-related protein | -Inf | Down |
| c183347_g5_i1 | heat shock protein 90 | -6.1243 | down |
| c179863_g2_i1 | Heat shock protein 90 | -Inf | down |
| c203483_g3_i3 | heat stress transcription factor A-4a | 1.366 | up |
| c198094_g1_i3 | heat stress transcription factor C-1-like | 2.173 | up |
| c198094_g1_i6 | heat stress transcription factor C-1-like | 3.513 | up |
| c107682_g1_i1 | heavy metal-associated isoprenylated plant protein 26 | -Inf | Down |
| c195854_g1_i5 | heme-binding-like protein | 1.014 | up |
| c194657_g1_i2 | HET-C domain protein HetC | 2.3907 | up |
| c187447_g1_i1 | hevein-like preproprotein | 1.3164 | up |
| c200763_g1_i5 | hexokinase-3 (LOC103871346), mRNA | 1.3866 | up |
| c204170_g1_i2 | high mobility group B protein 6 | 1.942 | up |
| c178336_g1_i1 | high-affinity nitrate transporter 3.1 | -1.1086 | Down |
| c205218_g1_i1 | HIPL2 protein | -1.7167 | Down |
| c203605_g1_i7 | histidine protein methyltransferase 1 homolog | Inf | up |
| c189008_g1_i1 | histidine-containing phosphotransfer protein 1 like | 1.1275 | UP |
| c201148_g2_i2 | histidine--tRNA ligase, cytoplasmic | -Inf | Down |
| c195395_g1_i1 | histone acetyltransferase | -4.0934 | Down |
| c195205_g1_i2 | histone acetyltransferase of the MYST family 1-like | -Inf | Down |
| c184478_g1_i3 | histone deacetylase complex subunit Sin3b | -5.013 | Down |
| c165052_g1_i1 | histone deacetylase HDT1 | -Inf | Down |
| c192505_g1_i2 | histone H2A | -Inf | Down |
| c183483_g1_i1 | histone H2A | -6.5912 | Down |
| c194613_g1_i5 | homeobox protein knotted-1-like 3 | Inf | up |
| c173658_g1_i5 | homeobox-leucine zipper protein HAT5-like | 2.6532 | up |
| c191972_g3_i3 | homeobox-leucine zipper protein HDG12 | -Inf | Down |
| c194701_g1_i1 | homologous-pairing protein 2 homolog | 2.7 | up |
| c194701_g1_i6 | homologous-pairing protein 2 homolog | 2.0477 | up |
| c194701_g1_i7 | homologous-pairing protein 2 homolog | 2.0622 | up |
| c203263_g3_i1 | Hsp90 binding co-chaperone | -2.7206 | Down |
| c193796_g2_i1 | hydrophobic protein RCI2A-like | 3.9449 | up |
| c201823_g1_i1 | hydroxyacyl-CoA dehydrogenase3-ketoacyl-CoA thiolaseenoyl-CoA hydratase | -3.7785 | Down |
| c204827_g4_i1 | hypersensitive-induced response protein 2 | 2.0565 | up |
| c204876_g2_i1 | IAA-amino acid hydrolase ILR1-like 6 | 1.4539 | up |
| c182301_g1_i2 | IMI 206040 protein mRNA | -5.0446 | Down |
| c182071_g1_i1 | inactive leucine-rich repeat receptor-like protein kinase | 2.5181 | up |
| c202966_g1_i8 | inactive poly [ADP-ribose] polymerase | 1.9011 | up |
| c191853_g1_i2 | inactive purple acid phosphatase 29 | 4.5352 | up |
| c205811_g1_i3 | inactive receptor kinase | 1.2492 | up |
| c194367_g1_i7 | inactive serinethreonine-protein kinase | 1.6847 | up |
| c194270_g1_i1 | inorganic phosphate transporter 1-3 | -1.1857 | Down |
| c194231_g1_i2 | inorganic phosphate transporter 1-4-like | 1.9501 | up |
| c124272_g2_i1 | inorganic phosphate transporter 1-5 | -Inf | Down |
| c204293_g1_i1 | inositol monophosphatase 1 | Inf | up |
| c191617_g1_i2 | inositol-3-phosphate synthase-like | 2.8655 | up |
| c189614_g1_i2 | inositolphosphorylceramide-B C-26 hydroxylase (gsl-5) gene | 3.0483 | up |
| c155262_g1_i2 | interacting protein 2 | -Inf | Down |
| c203317_g1_i1 | interferon-related developmental regulator 1 | 1.0639 | up |
| c193847_g1_i4 | IQ domain-containing protein IQM1-like | 3.008 | up |
| c189628_g1_i1 | ironascorbate oxidoreductase DDB_G0283291 | 1.5517 | up |
| c173086_g2_i6 | isochorismate synthase 1 | -2.6118 | Down |
| c194801_g2_i2 | isocitrate dehydrogenase | -5.0738 | Down |
| c199258_g1_i2 | isocitrate dehydrogenase [NAD] subunit gamma | -Inf | Down |
| c205389_g2_i3 | isoleucyl-tRNA synthetase (IARS), mRNA | -Inf | Down |
| c190186_g2_i1 | isopentenyl diphosphate isomerase | -Inf | Down |
| c179362_g3_i6 | isovaleryl-CoA dehydrogenase | -2.4628 | Down |
| c194701_g1_i3 | IST1-like protein | 3.1243 | up |
| c186418_g1_i3 | jacalin-related lectin 34-like | 2.0227 | up |
| c193728_g1_i1 | jacalin-related lectin 35-like | 1.5183 | up |
| c265669_g1_i1 | JAM81 protein | -3.4892 | Down |
| c165506_g1_i1 | jasmonate O-methyltransferase-like | 3.4754 | up |
| c188646_g1_i1 | keratin, type I cytoskeletal 10-like | 2.37 | up |
| c205076_g2_i2 | ketohexokinase | -2.8438 | Down |
| c177529_g1_i1 | kinase domain protein | -Inf | Down |
| c206705_g1_i1 | kinesin-4 | 1.5471 | up |
| c204466_g2_i3 | kinesin-like protein NACK1 | 2.5675 | up |
| c196994_g1_i2 | kinesin-related protein 4-like | -Inf | Down |
| c178900_g1_i1 | kininogen-1 | -Inf | Down |
| c147764_g1_i1 | KNP414 | -Inf | Down |
| c113763_g1_i1 | kunitz-type serine protease inhibitor DrTI-like | 1.9923 | up |
| c188761_g2_i3 | kunitz-type serine protease inhibitor DrTI-like | 3.4823 | up |
| c95881_g1_i1 | la ribonucleoprotein | -4.1067 | Down |
| c175478_g2_i1 | laccase (LAC) gene | -4.3815 | Down |
| c205810_g1_i1 | laccase-17 | 2.0105 | up |
| c189189_g1_i1 | laccase-1-like | 3.5154 | up |
| c147985_g1_i1 | large subunit ribosomal protein 23 | -5.4194 | Down |
| c326488_g1_i1 | large subunit ribosomal protein 23 (rpl-23) | -2.8204 | Down |
| c183365_g1_i3 | large subunit ribosomal protein L14 | -6.2578 | Down |
| c293483_g1_i1 | large subunit ribosomal RNA gene | -2.2596 | Down |
| c202783_g1_i5 | L-ascorbate oxidase homolog | 1.1536 | up |
| c202715_g1_i2 | leucine-rich repeat extensin-like protein 5 | 1.4153 | up |
| c203772_g1_i1 | leucine-rich repeat receptor-like protein kinase | -2.0705 | Down |
| c203772_g1_i2 | leucine-rich repeat receptor-like protein kinase | -Inf | Down |
| c203217_g6_i1 | leucine-rich repeat receptor-like protein kinase | -3.8067 | Down |
| c175061_g1_i1 | leucine-rich repeat receptor-like protein kinase PEPR2 | 3.5315 | up |
| c181012_g2_i1 | leucine-rich repeat receptor-like serinethreoninetyrosine-protein kinase SOBIR1 | 2.4071 | up |
| c194931_g2_i1 | leucine-rich repeat receptor-like serinethreoninetyrosine-protein kinase SOBIR1 | 2.5323 | up |
| c167968_g1_i1 | leucine-rich repeat-containing protein | -Inf | Down |
| c184742_g1_i1 | leucyl-tRNA synthetase | -Inf | down |
| c200839_g2_i1 | L-gulonolactone oxidase 2 (LOC106339293), mRNA | 2.4167 | up |
| c197121_g1_i7 | light-inducible protein CPRF2-like | 1.8567 | up |
| c199669_g2_i3 | LIM and SH3 domain protein 1-like | -Inf | Down |
| c200368_g1_i2 | LIM domain and actin-binding protein 1 | -6.2926 | Down |
| c203245_g2_i1 | lipase | 3.0959 | up |
| c203245_g2_i5 | lipase | 2.5949 | up |
| c185493_g1_i1 | lipase ROG1 | Inf | up |
| c205750_g1_i1 | lipase-like PAD4 | 2.2467 | up |
| c200895_g1_i2 | lipid phosphate phosphatase 1 | 2.0645 | up |
| c154485_g1_i1 | lipid-binding protein AIR1 | -2.7733 | Down |
| c182845_g1_i4 | lipid-transfer protein DIR1 | 2.1211 | up |
| c204007_g1_i1 | lipoxygenase 2, chloroplastic | 3.1408 | up |
| c193494_g1_i3 | lipoxygenase homology domain-containing protein 1-like | 1.8018 | up |
| c202939_g1_i3 | LOB domain-containing protein 41 | 2.1253 | up |
| c202939_g1_i1 | LOB domain-containing protein 41 | 1.0827 | up |
| c200216_g1_i1 | long chain acyl-CoA synthetase 8 | 1.0033 | uP |
| c186016_g1_i1 | loricrin-like | 1.5582 | up |
| c183129_g1_i2 | low-temperature-induced 65 kDa protein-like | Inf | up |
| c183129_g3_i4 | low-temperature-induced 65 kDa protein-like | Inf | up |
| c186720_g1_i1 | low-temperature-induced 65 kDa protein-like | 1.4355 | up |
| c7794_g2_i1 | LRR receptor-like protein kinase | -3.7224 | Down |
| c164988_g1_i1 | LRR receptor-like serinethreonine-protein kinase | -2.1571 | Down |
| c164988_g2_i1 | LRR receptor-like serinethreonine-protein kinase | -1.8275 | Down |
| c193476_g1_i1 | LRR receptor-like serinethreonine-protein kinase | -Inf | up |
| c192940_g1_i2 | LRR receptor-like serinethreonine-protein kinase | -Inf | down |
| c177272_g1_i2 | LRR receptor-like serinethreonine-protein kinase | -2.2746 | Down |
| c196067_g4_i2 | LRR receptor-like serinethreonine-protein kinase | -2.9245 | Down |
| c205225_g1_i2 | LRR receptor-like serinethreonine-protein kinase FLS2 | -1.2828 | Down |
| c203908_g1_i1 | L-type lectin-domain containing receptor kinase I.9 | 1.1835 | up |
| c201274_g1_i3 | L-type lectin-domain containing receptor kinase IV.2 | 1.2828 | up |
| c173494_g1_i1 | L-type lectin-domain containing receptor kinase IV.4-like | 6.4667 | up |
| c173494_g3_i1 | L-type lectin-domain containing receptor kinase IV.4-like | 4.7284 | up |
| c193136_g1_i2 | L-type lectin-domain containing receptor kinase V.2 | 3.4638 | up |
| c198259_g1_i1 | L-type lectin-domain containing receptor kinase V.2 | 3.3573 | up |
| c202606_g1_i1 | L-type lectin-domain containing receptor kinase V.5 | 1.2337 | up |
| c202606_g1_i3 | L-type lectin-domain containing receptor kinase V.5 | 1.0646 | up |
| c193136_g2_i4 | L-type lectin-domain containing receptor kinase V.9-like | 1.8714 | up |
| c197467_g1_i1 | lysine (K)-specific methyltransferase 2E | -Inf | Down |
| c195183_g1_i3 | lysine histidine transporter 1 | -1.5657 | Down |
| c204652_g2_i5 | lysine histidine transporter-like 7 | 2.8478 | up |
| c205080_g1_i2 | lysine-specific demethylase | 3.88 | up |
| c200882_g1_i3 | lysine-specific demethylase 5D | 1.8292 | up |
| c205080_g1_i3 | lysine-specific demethylase 5D | 2.2545 | up |
| c198261_g2_i4 | lysine-specific demethylase 5D-like | 2.692 | up |
| c203830_g1_i11 | lysine--tRNA ligase | -Inf | Down |
| c189575_g2_i1 | lysine--tRNA ligase-like | -Inf | Down |
| c205662_g1_i1 | MACPF domain-containing protein CAD1 | 1.4443 | up |
| c202753_g1_i2 | MACPF domain-containing protein NSL1 | 1.4443 | up |
| c197404_g2_i2 | MADS-box transcription factor 18-like | 3.7603 | up |
| c186426_g1_i1 | magnesium transporter NIPA2 | 2.4659 | up |
| c200013_g1_i1 | magnesium transporter NIPA6 | 2.0458 | up |
| c200013_g1_i3 | magnesium transporter NIPA6 | 2.4477 | up |
| c192386_g4_i1 | major centromere autoantigen B-like | 1.4858 | up |
| c182669_g2_i1 | major vault protein | -5.183 | Down |
| c196430_g1_i1 | mannan endo-1,4-beta-mannosidase 1 | 2.4742 | up |
| c196430_g1_i2 | mannan endo-1,4-beta-mannosidase 1 | 3.6177 | up |
| c173462_g1_i1 | mannitol dehydrogenase | -2.6693 | Down |
| c175268_g2_i2 | MAP kinase 5 | -Inf | up |
| c176716_g1_i2 | marmota lemur tyrosine kinase 2 | -4.8391 | Down |
| c175922_g1_i1 | MATE efflux family protein 5 | -4.1937 | Down |
| c198939_g1_i2 | MATE efflux family protein 5-like | -2.841 | Down |
| c200043_g1_i4 | mechanosensitive ion channel protein 10 | Inf | up |
| c205725_g1_i5 | mechanosensitive ion channel protein 6 | 1.425 | up |
| c173563_g2_i4 | mediator of RNA polymerase II transcription subunit 15a-like | 2.5052 | up |
| c199355_g1_i6 | mediator of RNA polymerase II transcription subunit 19a-like | -3.9914 | Down |
| c203734_g2_i4 | mediator of RNA polymerase II transcription subunit 26b | 1.0371 | up |
| c203734_g2_i5 | mediator of RNA polymerase II transcription subunit 26b | 1.9285 | up |
| c202410_g1_i1 | mediator of RNA polymerase II transcription subunit 37b | 2.2888 | up |
| c191586_g1_i2 | mediator-associated protein 1-like | 2.0393 | up |
| c148859_g1_i1 | Mel28 protein | 4.0061 | up |
| c149161_g1_i1 | Mel28 protein | 2.2703 | up |
| c175920_g1_i1 | Mel28 protein | 2.7334 | up |
| c182406_g1_i1 | Mel28 protein | 2.8812 | up |
| c174421_g1_i3 | membrane protein | 1.9288 | up |
| c181775_g2_i2 | membrane-anchored ubiquitin-fold protein 4-like | 4.1057 | up |
| c202192_g1_i3 | metacaspase-9 | 4.1382 | up |
| c195402_g3_i1 | metal ion binding | 2.4789 | up |
| c202862_g1_i1 | metalloendoproteinase | 1.5829 | up |
| c194651_g1_i1 | metalloendoproteinase 3-MMP | 1.4428 | up |
| c164064_g1_i1 | metalloprotease | -Inf | Down |
| c201323_g1_i1 | metastasis associated in colon cancer 1 | -1.1774 | Down |
| c203141_g4_i1 | methionine aminopeptidase | -3.3363 | Down |
| c193541_g2_i4 | methionine sulfoxide reductase MsrB-like | 3.6175 | up |
| c187797_g1_i1 | methyl-accepting chemotaxis protein | -5.2209 | Down |
| c201963_g1_i4 | methyl-CpG-binding domain-containing protein 10 | 1.0257 | up |
| c202456_g1_i2 | methylthioalkylmalate synthase 2 | 5.1487 | up |
| c192865_g4_i1 | methyltransferase DDB_G0268948 | 2.3498 | up |
| c200665_g1_i6 | methyltransferase DDB_G0268948 | 1.4715 | up |
| c200665_g1_i1 | methyltransferase DDB_G0268948 | 1.0992 | up |
| c167331_g2_i2 | methyltransferase PMT15 | -Inf | Down |
| c195549_g1_i2 | microtubule associated tumor suppressor candidate 2 | -5.4319 | Down |
| c203806_g1_i2 | microtubule-actin crosslinking factor 1 | -4.7926 | Down |
| c202954_g1_i2 | microtubule-associated protein 70 | 1.2801 | up |
| c202964_g1_i4 | microtubule-associated protein 70 | 1.5789 | up |
| c202964_g1_i8 | microtubule-associated protein 70 | 1.4871 | up |
| c170264_g1_i1 | microtubule-associated protein, RPEB family, | -5.529 | Down |
| c175565_g2_i1 | midkine (Mdk) | -Inf | Down |
| c167864_g1_i3 | mitochondrial amidoxime reducing component 2 | -1.1948 | Down |
| c200391_g1_i1 | mitochondrial ATP synthase subunit alpha precursor | -7.1892 | Down |
| c198186_g5_i1 | mitochondrial elongation factor 2 (EF2-1) mRNA | -Inf | Down |
| c200082_g1_i5 | mitochondrial substrate carrier family protein B | 3.2722 | up |
| c356238_g1_i1 | mitochondrial-processing peptidase subunit beta | -3.5822 | Down |
| c204123_g1_i4 | mitogen-activated protein kinase 18 | 1.516 | up |
| c198487_g1_i1 | mitogen-activated protein kinase kinase 4 | Inf | up |
| c194892_g3_i1 | mitogen-activated protein kinase kinase kinase 2-like | -4.6626 | Down |
| c203455_g1_i1 | mitogen-activated protein kinase kinase kinase NPK1 | 5.7268 | up |
| c194698_g1_i1 | MIZU-KUSSEI 1-like | -1.9796 | Down |
| c194698_g1_i2 | MIZU-KUSSEI 1-like | -Inf | Down |
| c193855_g1_i2 | MLO-like protein 2 | 1.8654 | up |
| c193855_g1_i6 | MLO-like protein 2 | 1.2711 | up |
| c200158_g1_i2 | MLO-like protein 8 | 1.4574 | up |
| c205019_g2_i1 | MLO-like protein2 | 1.139 | up |
| c190879_g1_i1 | MLP-like protein | -1.6475 | Down |
| c202078_g1_i3 | MND1-interacting protein 1 | -Inf | Down |
| c200510_g1_i1 | molybdenum cofactor sulfurase-like | 1.6175 | up |
| c202660_g2_i2 | mucin-5AC | 1.276 | up |
| c203798_g1_i1 | multiple C2 and transmembrane domain-containing protein 2 | 1.0334 | up |
| c204185_g1_i1 | MYA-3404 centromeremicrotubule-binding protein CBF5 | -5.3272 | Down |
| c201097_g1_i1 | myb-related protein 306-like | -1.4512 | Down |
| c197763_g1_i8 | myb-related protein 308-like | 3.609 | up |
| c197763_g1_i7 | myb-related protein Zm38-like | 4.0695 | up |
| c171031_g1_i2 | myeloid zinc finger 1 | -Inf | Down |
| c147846_g1_i1 | myosin, heavy chain | -4.8996 | Down |
| c147846_g1_i2 | myosin, heavy chain 6 | -4.9748 | Down |
| c200733_g1_i1 | myosin-binding protein 7-like | 1.7197 | up |
| c195416_g2_i1 | myrosinase-binding protein 2-like | 2.4589 | up |
| c196497_g1_i1 | myrosinase-binding protein-like | 1.4177 | up |
| c187622_g1_i2 | NAC domain-containing protein 102-like | 1.472 | up |
| c195944_g1_i8 | NAC domain-containing protein 17-like | 1.1754 | up |
| c201331_g2_i2 | NAC domain-containing protein 2 | 2.2139 | up |
| c202827_g1_i1 | NAC domain-containing protein 62 | 1.4201 | up |
| c192716_g1_i3 | NAC domain-containing protein 62-like | 1.9206 | up |
| c198795_g5_i2 | NAC domain-containing protein 62-like | 2.4794 | up |
| c198795_g6_i2 | NAC domain-containing protein 62-like | 2.4506 | up |
| c202827_g1_i3 | NAC domain-containing protein 62-like | 1.2355 | up |
| c196462_g2_i1 | NAC domain-containing protein 69-like | 2.5582 | up |
| c198089_g4_i1 | NAC domain-containing protein 69-like | 1.9404 | up |
| c194323_g4_i2 | NAC domain-containing protein 69-like | 3.525 | up |
| c203378_g1_i2 | NAC domain-containing protein 92 | 2.5312 | up |
| c189459_g1_i5 | NAC transcription factor 29-like | 2.2126 | up |
| c189459_g1_i8 | NAC transcription factor 29-like | 1.8605 | up |
| c198795_g4_i5 | NAC transcription factor 62-1.1 | 1.4962 | up |
| c182850_g2_i2 | NAC-domain protein 5-11 | 1.7557 | up |
| c180317_g2_i1 | nad dependent epimerase dehydratase family protein | -4.1922 | Down |
| c199759_g1_i3 | NAD(H) kinase 1-like | 3.6712 | up |
| c179220_g1_i2 | NAD-dependent epimerasedehydratase family protein | -5.0598 | Down |
| c176782_g2_i1 | NAD-dependent glutamate dehydrogenase | -5.6778 | down |
| c192102_g1_i2 | NADH dehydrogenase [ubiquinone] 1 alpha subcomplex subunit 2 | 1.583 | up |
| c155832_g2_i2 | NADHflavin oxidoreductase 1 | 3.2287 | up |
| c185884_g1_i2 | NADH-ubiquinone oxidoreductase | -Inf | Down |
| c205475_g1_i1 | NADP-dependent alkenal double bond reductase P1 | 1.0766 | up |
| c205475_g1_i2 | NADP-dependent alkenal double bond reductase P1 | 1.6089 | up |
| c202350_g1_i2 | NADP-dependent glyceraldehyde-3-phosphate dehydrogenase | 1.2718 | up |
| c192811_g1_i1 | nascent polypeptide-associated complex alpha subunit | -4.8081 | Down |
| c180191_g1_i1 | nectarin-1-like | -4.2164 | Down |
| c203535_g1_i1 | nematode resistance protein-like HSPRO2 | 2.1657 | up |
| c195406_g3_i2 | NEP1-interacting protein 1 | 1.8689 | up |
| c204523_g2_i1 | Niemann-Pick C1 protein | -Inf | Down |
| c199905_g1_i4 | NIH2624 amino-acid permease inda1 (ATEG_ | 1.4664 | up |
| c165729_g1_i1 | NIH2624 polyadenylate-binding protein | -3.2087 | Down |
| c176868_g3_i1 | NIH2624 protein | Inf | up |
| c189116_g3_i1 | ninja-family protein AFP1-like | 4.6371 | up |
| c200175_g1_i2 | ninja-family protein AFP3 | 3.5684 | up |
| c200175_g3_i1 | ninja-family protein AFP3 | 2.1174 | up |
| c202968_g1_i1 | ninja-family protein AFP4-like | -1.5302 | Down |
| c200571_g1_i1 | nitrile-specifier protein 2-like | 2.3748 | up |
| c204171_g1_i4 | non-lysosomal glucosylceramidase | 1.2884 | up |
| c189721_g1_i9 | non-lysosomal glucosylceramidase | -Inf | down |
| c200854_g1_i7 | non-lysosomal glucosylceramidase-like | 1.626 | up |
| c202767_g2_i1 | nonsense-mediated mRNA decay protein 2 | 1.4181 | up |
| c177021_g1_i1 | non-specific lipid-transfer protein 3 | 1.7263 | up |
| c187895_g1_i1 | non-specific lipid-transfer protein-like protein | 1.2805 | up |
| c155581_g1_i1 | NRRL 8126 protein | 3.2321 | up |
| c182959_g4_i1 | NRRL 8126 protein (THITE_2123173) | -2.9102 | Down |
| c202742_g1_i3 | NRRL3357 heat shock protein | Inf | up |
| c193030_g1_i3 | NRT1 PTR FAMILY 2.7 | -3.6047 | down |
| c197713_g1_i1 | NRT1 PTR FAMILY 4.6 | -1.0045 | down |
| c205398_g1_i2 | NRT1 PTR FAMILY 5.1 | -Inf | up |
| c205398_g1_i5 | NRT1 PTR FAMILY 5.1 | -Inf | down |
| c198694_g1_i1 | NRT1 PTR FAMILY 6.4 | 1.1179 | up |
| c197513_g1_i1 | nuclear export mediator factor (NEMF) | -4.4343 | Down |
| c191690_g1_i1 | nuclear lim interactor-interacting | -Inf | down |
| c117525_g1_i1 | nuclear phosphoprotein 32 family | -Inf | Down |
| c178192_g1_i1 | nuclear receptor subfamily 2, group C, member 1 (NR2C1), | -Inf | Down |
| c185566_g1_i2 | nuclear transport factor 2 | -4.5894 | Down |
| c201314_g2_i1 | nuclease HARBI1 | -1.52 | Down |
| c201399_g1_i6 | nucleobase-ascorbate transporter 6 | 1.0584 | up |
| c185156_g2_i1 | nucleoporin 210 | -Inf | Down |
| c183490_g1_i1 | nucleoside diphosphate kinase | -7.2508 | Down |
| c183838_g1_i4 | nucleoside diphosphate kinase | -Inf | Down |
| c135531_g1_i1 | nucleoside diphosphate kinase 1 | -Inf | down |
| c199854_g1_i1 | nucleostemin-like protein | -5.4343 | Down |
| c202597_g1_i3 | nudix hydrolase 15, mitochondrial | 1.5395 | up |
| c201656_g2_i2 | nudix hydrolase 3-like | -5.0091 | Down |
| c199826_g2_i1 | nudix hydrolase 8 | 1.7393 | up |
| c175262_g2_i1 | O-acyltransferase WSD1-like | Inf | up |
| c96077_g1_i1 | Ocm1 mRNA for tubular mastigoneme protein | -Inf | Down |
| c198085_g1_i2 | ocs element-binding factor 1-like | 3.4873 | up |
| c193810_g2_i1 | omega-3 fatty acid desaturase | 2.0156 | up |
| c158915_g1_i1 | omega-hydroxypalmitate O-feruloyl transferase | -3.335 | down |
| c206261_g1_i1 | organic cationcarnitine transporter 6 | 3.1556 | up |
| c196457_g1_i3 | ornithine aminotransferase, mitochondrial-like | -5.0729 | Down |
| c190858_g6_i1 | oryzain alpha chain-like | -Inf | Down |
| c205555_g1_i8 | Outer arm dynein light chain 1 protein, putative isoform 2 | 1.1509 | up |
| c204412_g1_i2 | oxysterol-binding protein-related protein | -Inf | Down |
| c204822_g1_i1 | palmitoyl-acyl carrier protein thioesterase | 1.397 | up |
| c191890_g1_i1 | palmitoyl-acyl carrier protein thioesterase | 1.6531 | up |
| c203827_g1_i1 | PAN domain-containing protein At5g03700 | -1.0772 | Down |
| c95634_g1_i1 | papain family cysteine protease subfamily protein | -Inf | Down |
| c193950_g1_i1 | patatin-like protein 1 | 3.7166 | up |
| c193494_g1_i1 | pathogenesis-related protein 5-like | 3.2459 | up |
| c193533_g1_i2 | pathogenesis-related protein 5-like | 1.5286 | up |
| c180185_g1_i1 | pathogenesis-related protein PR-4- | 1.5984 | up |
| c178883_g1_i1 | PB1_UP2 domain-containing protein | -1.623 | Down |
| c159621_g1_i2 | pectate lyase 14 | 3.1011 | up |
| c192502_g1_i1 | pectinesterase 31 | 1.2688 | up |
| c156607_g2_i3 | pectinesterasepectinesterase inhibitor 33 | Inf | up |
| c156607_g2_i6 | pectinesterasepectinesterase inhibitor 33 | 2.8713 | up |
| c200293_g1_i1 | pectinesterasepectinesterase inhibitor 35 | -1.4717 | Down |
| c170694_g3_i1 | pentatricopeptide repeat protein | -2.5259 | Down |
| c188248_g1_i1 | pentatricopeptide repeat-containing protein | 2.3251 | up |
| c188248_g1_i2 | pentatricopeptide repeat-containing protein | 1.5797 | up |
| c189958_g1_i2 | pentatricopeptide repeat-containing protein | 2.1538 | up |
| c204165_g3_i1 | pentatricopeptide repeat-containing protein | 1.2549 | up |
| c206709_g1_i11 | pentatricopeptide repeat-containing protein | -4.2018 | Down |
| c182575_g2_i1 | pentatricopeptide repeat-containing protein At1g31790-like | 1.023 | up |
| c168528_g1_i3 | peptide chain release factor 1 | -Inf | down |
| c203604_g1_i1 | peptidoglycan-binding LysM domain-containing protein | 2.3576 | up |
| c188856_g2_i2 | peptidyl-prolyl cis-trans isomerase | -Inf | down |
| c197192_g1_i1 | peptidyl-prolyl cis-trans isomerase CYP40-like | -6.2452 | Down |
| c192481_g1_i2 | Peroxidase | -Inf | Down |
| c192481_g1_i3 | peroxidase | -Inf | Down |
| c195689_g1_i3 | peroxidase 58 | 1.129 | up |
| c189849_g1_i1 | peroxidase 66-like | 2.1163 | up |
| c198978_g1_i1 | peroxidase A2 | 3.5001 | up |
| c198978_g1_i2 | peroxidase A2 | 3.086 | up |
| c198978_g1_i3 | peroxidase A2-like | 2.446 | up |
| c1141_g1_i1 | peroxiredoxin | -Inf | Down |
| c188103_g1_i2 | peroxisomal (S)-2-hydroxy-acid oxidase GLO1-like | 1.1316 | up |
| c201776_g1_i1 | peroxisomal adenine nucleotide carrier 2 | 2.6687 | up |
| c201776_g1_i3 | peroxisomal adenine nucleotide carrier 2 | 2.0083 | up |
| c185486_g2_i1 | peroxygenase 4 | 1.3197 | up |
| c176000_g1_i3 | PHD finger protein 6-like | -Inf | Down |
| c190346_g1_i3 | phosphatase SPAC5H10.03 | 2.4871 | up |
| c208101_g1_i1 | phosphate transporter | -6.3216 | Down |
| c201117_g1_i8 | phosphate transporter PHO1 homolog 5 | 2.9708 | up |
| c194176_g1_i2 | phosphatidylcholine transfer protein | 1.3105 | up |
| c205827_g1_i3 | phosphatidylinositol 4-kinase gamma 4 | 1.1907 | up |
| c201901_g1_i1 | phosphatidylinositol 4-phosphate 5-kinase 5-like | -1.5885 | Down |
| c202352_g3_i1 | phosphatidylinositolceramide inositolphosphotransferase 1 | 4.0905 | up |
| c202352_g4_i1 | phosphatidylinositolceramide inositolphosphotransferase 1 | 4.5619 | up |
| c202352_g4_i6 | phosphatidylinositolceramide inositolphosphotransferase 1 | 3.5619 | up |
| c202352_g4_i5 | phosphatidylinositolceramide inositolphosphotransferase 1 | 4.0555 | up |
| c196993_g1_i2 | phosphatidylinositolceramide inositolphosphotransferase 2-like | 2.0155 | up |
| c196993_g1_i3 | phosphatidylinositolceramide inositolphosphotransferase 2-like | 2.2615 | up |
| c196993_g1_i5 | phosphatidylinositolceramide inositolphosphotransferase 2-like | 1.8114 | up |
| c203653_g1_i2 | phosphoenolpyruvate carboxykinase | -Inf | Down |
| c190346_g1_i4 | phosphoglycerate mutase-like protein 2 | Inf | up |
| c194444_g1_i2 | phospholipase A I | 2.2226 | up |
| c192618_g1_i1 | phospholipase A1-Igamma1 | 1.4452 | up |
| c195141_g2_i1 | phospholipid hydroperoxide glutathione peroxidase 6 | 1.3206 | up |
| c195141_g2_i3 | phospholipid hydroperoxide glutathione peroxidase 6 | Inf | up |
| c199800_g1_i1 | phospholipid-transporting ATPase 10 | 1.5178 | up |
| c194044_g1_i1 | phospholipid-transporting ATPase 11 | 1.4187 | up |
| c185971_g1_i2 | phospholipid-transporting ATPase 5 | 1.9591 | up |
| c195448_g2_i2 | phospholipid-transporting ATPase 5 | 2.9546 | up |
| c196053_g2_i3 | phospholipid-transporting ATPase 5 | 2.9607 | up |
| c198369_g1_i1 | phosphomethylpyrimidine synthase | 1.0122 | up |
| c185495_g1_i1 | phytosulfokines 2-like | 1.4453 | up |
| c183162_g4_i1 | pirin-like protein | 1.8371 | up |
| c260658_g1_i1 | plasma membrane ATPase-like | -Inf | Down |
| c162626_g3_i1 | plasma membrane proteolipid 3 partial mRNA | 2.1659 | up |
| c200421_g1_i7 | PLASMODESMATA CALLOSE-BINDING PROTEIN 3-like | -2.8791 | Down |
| c146071_g1_i1 | pleiotropic drug resistance protein | -Inf | Down |
| c201150_g1_i1 | pollen-specific protein SF21 | 2.4829 | up |
| c162133_g1_i1 | poly(U)-binding-splicing factor half pint | -Inf | Down |
| c206585_g1_i1 | polyadenylate binding protein | -Inf | Down |
| c142048_g1_i1 | polyadenylate protein with binding domain | -3.9054 | Down |
| c202480_g1_i2 | polyamine oxidase 4 | -1.4059 | Down |
| c200920_g1_i3 | polygalacturonase inhibitor 1-like | -2.3462 | Down |
| c200951_g1_i4 | polymerase (RNA) III (DNA directed) polypeptide | -Inf | Down |
| c203066_g1_i1 | polyol transporter 6 | -1.4758 | Down |
| c195757_g1_i3 | polypyrimidine tract-binding protein 2-like | 2.1326 | up |
| c156760_g1_i1 | polyubiquitin-like | 2.4956 | up |
| c197830_g2_i3 | potassium transporter 9-like | 3.3233 | up |
| c197830_g3_i1 | potassium transporter 9-like | Inf | up |
| c197830_g3_i2 | potassium transporter 9-like | 4.15 | up |
| c197830_g4_i3 | potassium transporter 9-like | 3.5458 | up |
| c197830_g4_i4 | potassium transporter 9-like | 2.9244 | up |
| c199533_g1_i2 | potassium voltage-gated channel, shaker-related subfamily, beta member 2 | -3.4609 | Down |
| c198724_g2_i3 | PRA1 family protein A3 | 2.5136 | up |
| c198724_g2_i4 | PRA1 family protein A3 | 3.2085 | up |
| c190232_g1_i2 | PRA1 family protein B5 | 2.9271 | up |
| c196988_g1_i5 | PRA1 family protein F3 | 2.7312 | up |
| c195306_g2_i4 | PRA1 family protein F3-like | 1.6289 | up |
| c203903_g1_i1 | prefoldin subunit 2 | -5.1457 | Down |
| c161655_g2_i1 | pre-mRNA-splicing factor CLF1-like | Inf | up |
| c198628_g1_i1 | pre-rRNA processing protein FTSJ3-like | -4.8577 | Down |
| c156392_g1_i1 | presenilin 2 | -5.7021 | Down |
| c186587_g1_i3 | profilin | -Inf | Down |
| c186587_g1_i2 | profilin | -5.2561 | Down |
| c155060_g1_i1 | proliferating cell nuclear antigen | -Inf | Down |
| c29069_g2_i1 | proliferation-associated protein 2G4 | -Inf | Down |
| c201592_g1_i1 | proline dehydrogenase 1 | -1.207 | down |
| c177304_g2_i1 | proline dehydrogenase 2 | -1.5676 | Down |
| c177304_g1_i1 | proline dehydrogenase 2 | -1.1964 | down |
| c186746_g1_i1 | proline-rich protein 1 | -5.1361 | Down |
| c194943_g3_i3 | proline-rich receptor-like protein kinase PERK1 | 1.4481 | up |
| c184542_g1_i1 | proline-rich SH3 domain protein | -Inf | Down |
| c194937_g1_i1 | prolyl endopeptidase-like | 1.5814 | up |
| c175312_g1_i1 | protease inhibitor HPI-like | -1.1376 | Down |
| c182509_g1_i2 | protease regulatory subunit 6B | -Inf | Down |
| c194120_g1_i2 | Proteasome | -Inf | Down |
| c204320_g1_i1 | proteasome non-ATPase regulatory subunit | -Inf | Down |
| c197831_g3_i2 | proteasome non-ATPase regulatory subunit 14-like | -4.1961 | Down |
| c205023_g1_i1 | proteasome non-ATPase regulatory subunit 8 homolog A | 1.1487 | up |
| c188524_g1_i1 | proteasome non-ATPase regulatory subunit 9-like | 1.9544 | up |
| c193509_g1_i3 | proteasome non-ATPase regulatory subunit Nin1 | -Inf | down |
| c195815_g1_i5 | proteasome regulatory particle subunit | -Inf | Down |
| c202404_g1_i1 | proteasome regulatory subunit RPN7 | -Inf | down |
| c207110_g1_i4 | proteasome regulatory subunit S2, mRNA | -5.2587 | down |
| c194106_g1_i1 | proteasome subunit alpha type 5 | -4.8055 | Down |
| c196004_g1_i1 | proteasome subunit alpha type-2-like | -5.3046 | Down |
| c196649_g1_i1 | proteasome subunit alpha type-7 | -Inf | Down |
| c194029_g1_i2 | proteasome, A and B subunits | -3.1655 | Down |
| c52103_g1_i1 | protein | -4.2393 | Down |
| c202879_g3_i1 | protein ABSCISIC ACID-INSENSITIVE 5 | 1.8904 | up |
| c201808_g1_i3 | protein arginine N-methyltransferase 1-like | -6.5905 | Down |
| c204885_g1_i1 | protein ATAF2 | 1.6777 | up |
| c204885_g1_i7 | protein ATAF2 (NAC3) | 1.5625 | up |
| c192716_g1_i1 | protein ATAF2-like | 2.1422 | up |
| c205058_g2_i1 | protein AUXIN-REGULATED GENE INVOLVED IN ORGAN SIZE | 2.1523 | up |
| c194288_g1_i1 | protein BREAST CANCER SUSCEPTIBILITY 1 homolog | 1.2266 | up |
| c192769_g4_i5 | protein C2-DOMAIN ABA-RELATED 1 | -1.4292 | Down |
| c192901_g1_i2 | protein C2-DOMAIN ABA-RELATED 7 | 1.1138 | up |
| c196174_g1_i4 | protein C2orf69-like | 3.4817 | up |
| c196174_g1_i7 | protein C2orf69-like | 5.0828 | up |
| c133814_g1_i2 | protein CASC3 | -3.5393 | Down |
| c198742_g1_i3 | protein CDI | -Inf | up |
| c164161_g1_i1 | protein CHLORORESPIRATORY REDUCTION 6 | -2.4727 | Down |
| c175083_g1_i2 | protein CHU_1773-like | -2.4274 | Down |
| c203274_g1_i2 | protein DA1-related 6 | 2.5474 | up |
| c203274_g3_i1 | protein DA1-related 6 | 2.0015 | up |
| c180532_g1_i2 | protein disulfide-isomerase | -Inf | Down |
| c122453_g2_i1 | protein disulfide-isomerase domain partial | -Inf | Down |
| c200550_g2_i2 | protein EARLY FLOWERING 3-like | -2.9587 | Down |
| c200550_g1_i5 | protein EARLY FLOWERING 3-like | -4.7033 | Down |
| c197127_g1_i3 | protein EARLY RESPONSIVE TO DEHYDRATION 15-like | 1.7794 | up |
| c179081_g1_i1 | protein ECU03_1610-like | 1.6669 | up |
| c104045_g1_i1 | protein FAM49B-like | -4.9274 | Down |
| c197329_g1_i2 | protein FIZZY-RELATED 2-like | -Inf | Down |
| c176692_g1_i4 | protein FIZZY-RELATED 3 | -Inf | Down |
| c201706_g1_i2 | protein GPR107 | 2.5734 | up |
| c189409_g1_i4 | protein HAIKU1-like | 1.2451 | up |
| c189409_g1_i5 | protein HAIKU1-like | 1.4438 | up |
| c193757_g2_i1 | protein HOTHEAD-like | -1.645 | Down |
| c190923_g2_i2 | protein kinase | 3.0478 | up |
| c171454_g1_i1 | protein kinase C, alpha (PRKCA) gene | -2.4206 | Down |
| c203689_g1_i3 | protein kinase PINOID | 2.544 | up |
| c176484_g1_i1 | protein LIKE COV 1 | -Inf | Down |
| c203888_g1_i1 | protein LONGIFOLIA 2 | -1.2685 | Down |
| c195686_g2_i2 | protein LSD1 | 1.7472 | up |
| c192014_g1_i1 | protein LURP-one-related 10 | 3.0507 | up |
| c192454_g1_i1 | protein LURP-one-related 12-like | 1.2274 | up |
| c188497_g2_i1 | protein LURP-one-related 5 | 3.1713 | up |
| c188497_g1_i1 | protein LURP-one-related 5-like | 3.6741 | up |
| c132610_g1_i2 | protein LURP-one-related 8 | -1.0202 | Down |
| c201106_g2_i8 | protein MEMO1-like | -3.5139 | Down |
| c201786_g1_i | protein MKS1 | 3.6318 | up |
| c201786_g1_i2 | protein MKS1 | 3.6318 | up |
| c116647_g1_i1 | protein MON2 homolog | -Inf | Down |
| c199521_g1_i2 | protein NETWORKED 4B-like | 1.6907 | up |
| c199521_g1_i3 | protein NETWORKED 4B-like | 1.7946 | up |
| c199521_g1_i5 | protein NETWORKED 4B-like | 2.9174 | up |
| c199521_g1_i6 | protein NETWORKED 4B-like | 2.2091 | up |
| c193030_g1_i2 | protein NRT1 PTR FAMILY 2.7-like | -3.6047 | Down |
| c193030_g1_i1 | protein NRT1 PTR FAMILY 2.7-like | -2.5898 | Down |
| c202497_g1_i1 | protein NRT1 PTR FAMILY 2.10 | 1.4551 | up |
| c193474_g1_i1 | protein NRT1 PTR FAMILY 2.7 | 1.5934 | up |
| c202497_g2_i2 | protein NRT1 PTR FAMILY 2.9 | 1.4129 | up |
| c204489_g1_i1 | protein NtpR | -1.233 | Down |
| c196266_g2_i3 | protein OBERON 3-like | 1.0762 | up |
| c202059_g1_i1 | protein phosphatase 2C | 2.7175 | up |
| c203162_g1_i3 | protein phosphatase 2C | 2.6727 | up |
| c203261_g1_i1 | protein phosphatase 2C 14 | 1.003 | up |
| c201161_g1_i3 | protein phosphatase 2C 37 | 1.0528 | up |
| c198233_g1_i5 | protein phosphatase 2C 38 | -3.0688 | Down |
| c206318_g1_i5 | protein phosphatase 2C 60 | -1.4249 | Down |
| c201208_g1_i1 | protein phosphatase 2C 61 | -1.9745 | Down |
| c197775_g1_i1 | protein phosphatase 2C 63 | 2.3722 | up |
| c204899_g1_i1 | protein phosphatase 2C 66 | -1.5934 | Down |
| c199055_g1_i1 | protein phosphatase 2C 73 | 2.2189 | up |
| c204380_g2_i2 | protein phosphatase 2C 77 | 2.2995 | up |
| c204380_g2_i3 | protein phosphatase 2C 77 | 2.9329 | up |
| c196867_g1_i1 | protein phosphatase 2C 78 | 2.5473 | up |
| c194405_g1_i1 | protein phosphatase 2c 9 | 1.1819 | up |
| c194405_g1_i6 | protein phosphatase 2C 9 | 1.4905 | up |
| c188755_g4_i1 | protein phosphatase inhibitor 2-like | 2.4294 | up |
| c188755_g4_i2 | protein phosphatase inhibitor 2-like | 2.0645 | up |
| c156096_g1_i1 | protein pygopus-like | -Inf | Down |
| c146079_g1_i1 | protein RALF-like 32 | 1.2735 | up |
| c203087_g1_i1 | protein REVEILLE 2-like | -Inf | Down |
| c197792_g1_i1 | protein starmaker | 3.5199 | up |
| c197792_g1_i2 | protein starmaker-like | 2.3478 | up |
| c199826_g1_i1 | protein starmaker-like | 1.7521 | up |
| c205404_g1_i1 | protein STRUBBELIG-RECEPTOR FAMILY 7 | 1.2967 | up |
| c198525_g3_i7 | protein SUPPRESSOR OF npr1-1, CONSTITUTIVE 1-like | 1.7315 | up |
| c190742_g3_i1 | protein TIFY 10B | 1.396 | up |
| c191109_g2_i1 | protein TIFY 11A-like | 1.825 | up |
| c196566_g2_i3 | protein TIFY 11B-like | 1.7266 | up |
| c203147_g4_i1 | protein TIFY 5A | 3.3757 | up |
| c198169_g1_i5 | protein TIFY 6B | 1.7731 | up |
| c196817_g1_i4 | protein TIFY 7 | 1.4632 | up |
| c200360_g2_i1 | protein TPRXL | -1.1936 | Down |
| c203581_g2_i1 | protein translation factor SUI1 homolog | 1.2362 | up |
| c204248_g1_i1 | protein TRANSPARENT TESTA 1 | 2.0469 | up |
| c204490_g1_i2 | protein transport protein SEC23 | -Inf | Down |
| c164015_g1_i1 | protein transport protein Sec61 subunit alpha-like 1 | -Inf | down |
| c204849_g2_i1 | protein TRM32 | 2.2013 | up |
| c162233_g1_i2 | protein tyrosine phosphatase, receptor type | -1.9426 | Down |
| c175143_g1_i4 | protein VASCULAR ASSOCIATED DEATH 1 | Inf | up |
| c199446_g1_i3 | protein WALLS ARE THIN 1-like | 1.5743 | up |
| c173034_g1_i3 | protein ycf36-like | 1.0969 | up |
| c193230_g1_i1 | protein YLS9 | 1.6057 | up |
| c193230_g1_i3 | protein YLS9 | 3.1105 | up |
| c193343_g1_i3 | protein YLS9 | 1.6745 | up |
| c196384_g2_i2 | protein YLS9-like | -Inf | Down |
| c196997_g1_i1 | protein YLS9-like | 1.7541 | up |
| c176137_g1_i2 | proteinase R | -Inf | Down |
| c191915_g1_i1 | PTI1-like tyrosine-protein kinase 3 | 2.2149 | up |
| c191915_g3_i1 | PTI1-like tyrosine-protein kinase 3 | 2.3315 | up |
| c188388_g1_i1 | p-type calcium transporter | 2.515 | up |
| c205676_g1_i3 | P-type II calcium ATPase | -4.4222 | Down |
| c116647_g2_i1 | pumilio domain-containing protein KIAA0020 homolog | -Inf | Down |
| c204544_g2_i1 | purine permease 10 | 1.0335 | up |
| c202849_g1_i3 | purine permease 14 | 2.1265 | up |
| c202849_g1_i5 | purine permease 14 | 2.027 | up |
| c189099_g1_i1 | purine permease 18 | 1.2301 | up |
| c189099_g1_i2 | purine permease 18 | 1.1739 | up |
| c188489_g1_i1 | purine phosphorylase family protein | -Inf | Down |
| c195791_g1_i1 | purple acid phosphatase 10-like | -1.3819 | Down |
| c189341_g2_i1 | PYK10-binding protein 2-like | 1.4601 | up |
| c183026_g1_i1 | pyrophosphate-energized vacuolar membrane proton pump | -1.6653 | down |
| c128204_g1_i1 | pyrophosphate-energized vacuolar membrane proton pump 1 | -Inf | Down |
| c189894_g2_i2 | pyrophosphate-energized vacuolar membrane proton pump 1-like | -1.6095 | Down |
| c183026_g3_i1 | pyrophosphate-energized vacuolar membrane proton pump 1-like (AVP1-2) | -1.7812 | Down |
| c277898_g1_i1 | pyrophosphate-energized vacuolar membrane proton pump-like | -Inf | Down |
| c195447_g1_i2 | pyrroline-5-carboxylate reductase-like | 1.2186 | up |
| c204506_g1_i1 | pyruvate kinase, cytosolic isozyme | 1.2922 | up |
| c204506_g1_i2 | pyruvate kinase, cytosolic isozyme | 1.3898 | up |
| c190583_g2_i1 | pyruvate kinase, cytosolic isozyme-like | 2.1645 | up |
| c183938_g3_i2 | quinone oxidoreductase-like protein | 1.2699 | up |
| c186773_g3_i1 | rab family small GTPase | -4.3445 | Down |
| c187485_g1_i1 | rab family small GTPase | -Inf | Down |
| c204172_g1_i7 | rab3 GTPase-activating protein catalytic subunit | -2.3676 | Down |
| c201901_g1_i4 | radial spoke head 10 homolog B-like | -1.3599 | Down |
| c201901_g1_i3 | radial spoke head 10 homolog B-like | -1.371 | Down |
| c189502_g1_i1 | ran family small GTPase | -5.5049 | Down |
| c199136_g1_i2 | random slug protein 5-like | 2.6918 | up |
| c195091_g1_i3 | Ras association (RalGDSAF-6) and pleckstrin homology domains 1 | 3.4406 | up |
| c195091_g1_i7 | Ras association (RalGDSAF-6) and pleckstrin homology domains 1 | 3.429 | up |
| c195766_g1_i1 | Ras-like C3 botulinum toxin substrate 1 | -2.6552 | Down |
| c189634_g2_i2 | ras-related C3 botulinum toxin substrate 2 | -4.4513 | Down |
| c177361_g1_i1 | ras-related protein Rab-11A | -Inf | Down |
| c190175_g1_i5 | ras-related protein RABA1c | 2.8947 | up |
| c194905_g1_i1 | ras-related protein RABA2c | 2.7828 | up |
| c194905_g2_i1 | ras-related protein RABA2c | 1.8727 | up |
| c181673_g3_i1 | ras-related protein RABA2d (LOC103851600), mRNA | -1.654 | Down |
| c181673_g2_i3 | ras-related protein RABA2d-like | -2.1248 | Down |
| c180279_g1_i1 | ras-related protein RABH1e-like | 2.4579 | up |
| c178767_g1_i1 | receptor coactivator 1 (NCOA1) | -3.4503 | Down |
| c204107_g1_i1 | receptor-like protein 12 | 1.9895 | up |
| c204107_g1_i2 | receptor-like protein 12 | 1.0707 | up |
| c191455_g5_i1 | receptor-like protein 12 | 2.4427 | up |
| c194523_g1_i2 | receptor-like protein 12 | -1.457 | Down |
| c196949_g4_i4 | receptor-like protein 12 | 2.2254 | up |
| c197538_g4_i1 | receptor-like protein 12 | 1.6753 | up |
| c204585_g1_i1 | receptor-like protein 12 | 1.6741 | up |
| c204759_g1_i1 | receptor-like protein 12 | 1.4262 | up |
| c205249_g1_i3 | receptor-like protein 12 | 1.272 | up |
| c187991_g1_i2 | receptor-like protein 12 | 1.2449 | up |
| c201766_g1_i1 | receptor-like protein kinase | 2.051 | up |
| c198638_g1_i1 | receptor-like protein kinase | 5.0365 | up |
| c198747_g1_i1 | receptor-like protein kinase | 2.9287 | up |
| c199714_g1_i2 | receptor-like protein kinase | 2.0544 | up |
| c202666_g2_i1 | receptor-like protein kinase | 2.3002 | up |
| c201766_g1_i3 | receptor-like protein kinase | 1.1382 | up |
| c201766_g1_i4 | receptor-like protein kinase | 1.5263 | up |
| c189326_g3_i1 | receptor-like protein kinase | 3.305 | up |
| c206303_g1_i1 | receptor-like protein kinase | 1.1395 | up |
| c195810_g3_i1 | receptor-like protein kinase | 1.8065 | up |
| c196386_g1_i5 | receptor-like protein kinase | 2.2547 | up |
| c202666_g1_i2 | receptor-like protein kinase 5 | 1.8823 | up |
| c202666_g1_i1 | receptor-like protein kinase 5 | 1.4773 | up |
| c185371_g3_i1 | receptor-like protein kinase FERONIA | 1.1452 | up |
| c203474_g1_i1 | receptor-like protein kinase FERONIA | 1.3902 | up |
| c179611_g1_i1 | receptor-like protein kinase HAIKU2 | 1.1051 | up |
| c203225_g1_i2 | receptor-like serinethreonine-protein kinase | -1.005 | down |
| c205646_g1_i1 | receptor-like serinethreonine-protein kinase | 1.382 | up |
| c194931_g1_i2 | receptor-like serinethreoninetyrosine-protein kinase | 1.9834 | up |
| c202969_g2_i1 | receptor-like serinethreoninetyrosine-protein kinase | 2.5955 | up |
| c200321_g1_i1 | red chlorophyll catabolite reductase | 1.0789 | up |
| c196328_g1_i3 | REFSRPP-like protein | 1.0318 | up |
| c200526_g1_i4 | REFSRPP-like protein | 2.4172 | up |
| c200526_g1_i5 | REFSRPP-like protein | 2.151 | up |
| c200526_g1_i3 | REFSRPP-like protein | 3.1663 | up |
| c200159_g1_i1 | regulatory protein NPR2 | 1.5891 | up |
| c200159_g1_i2 | regulatory protein NPR2 | 1.6063 | up |
| c200159_g1_i3 | regulatory protein NPR2 | 1.6942 | up |
| c200159_g1_i4 | regulatory protein NPR2 | 1.8803 | up |
| c205439_g1_i3 | regulatory protein NPR3 | 1.1172 | up |
| c199559_g1_i1 | Remorin | 1.5694 | up |
| c188075_g1_i4 | replication protein A 70 kDa DNA-binding subunit A-like | -1.3107 | Down |
| c178998_g1_i1 | respiratory burst oxidase homolog protein D-like | 1.556 | up |
| c179053_g1_i2 | respiratory burst oxidase homolog protein D-like | 2.0842 | up |
| c189501_g3_i1 | respiratory burst oxidase homolog protein D-like | 1.6041 | up |
| c189501_g4_i1 | respiratory burst oxidase homolog protein D-like | 1.6657 | up |
| c203290_g1_i1 | reticuline oxidase | -2.0778 | Down |
| c181149_g1_i1 | reticuline oxidase-like protein | -1.2697 | Down |
| c194685_g2_i1 | reticulon-like protein B1 | 2.6568 | up |
| c194685_g2_i2 | reticulon-like protein B1 | 1.4295 | up |
| c188071_g1_i1 | retinal dehydrogenase 1-like | -Inf | Down |
| c189634_g4_i2 | rho family small GTPase | -3.7886 | Down |
| c191898_g2_i2 | rho family small GTPase | -5.1457 | Down |
| c197597_g1_i1 | RHOMBOID-like protein 2 | 1.1846 | up |
| c176744_g1_i1 | rhophilin-2 | -Inf | Down |
| c190040_g4_i1 | ribonuclease 1 | 2.1148 | up |
| c190040_g4_i3 | ribonuclease 1-like | 2.5813 | up |
| c96661_g1_i1 | ribonuclease H protein | -5.5809 | Down |
| c199669_g2_i2 | ribonuclease P protein subunit p25-like protein | -5.3948 | Down |
| c189877_g1_i1 | ribonucleoprotein | -2.6038 | Down |
| c174895_g2_i1 | ribonucleoprotein complex subunit 4-like | -5.0729 | Down |
| c184602_g3_i1 | ribosomal DNA intergenic spacer pESIGS3 | -1.1363 | Down |
| c204259_g1_i2 | ribosomal export protein | -3.3618 | Down |
| c110397_g1_i1 | ribosomal protein | -2.674 | Down |
| c129399_g1_i1 | ribosomal protein | -3.4724 | Down |
| c143237_g1_i1 | ribosomal protein | -4.0262 | Down |
| c143453_g1_i1 | ribosomal protein | -3.2532 | Down |
| c144359_g2_i1 | ribosomal protein | -Inf | Down |
| c148370_g1_i1 | ribosomal protein | -5.7772 | Down |
| c150472_g1_i1 | ribosomal protein | -3.4542 | Down |
| c150561_g1_i1 | ribosomal protein | -Inf | Down |
| c192452_g1_i2 | ribosomal protein | -2.3151 | Down |
| c193560_g1_i1 | ribosomal protein | -3.6408 | Down |
| c182600_g2_i1 | ribosomal protein | -4.7056 | Down |
| c141876_g2_i1 | ribosomal protein | -5.3926 | down |
| c81778_g1_i1 | ribosomal protein | -5.5022 | down |
| c266439_g1_i1 | ribosomal protein | -5.8742 | Down |
| c60537_g2_i1 | ribosomal protein | -3.6141 | down |
| c161259_g1_i1 | ribosomal protein – like | -2.2832 | Down |
| c60866_g1_i1 | ribosomal protein 27 (rps-27) | -3.9941 | Down |
| c163030_g1_i1 | ribosomal protein L1 | -3.8626 | Down |
| c166443_g1_i1 | ribosomal protein L1 | -Inf | Down |
| c154500_g1_i1 | ribosomal protein L10 | -Inf | Down |
| c189100_g2_i4 | ribosomal protein L10 | -3.3086 | down |
| c229886_g1_i1 | ribosomal protein L10 | -5.9328 | down |
| c160020_g1_i1 | ribosomal protein L10a | -5.72 | Down |
| c160020_g2_i1 | ribosomal protein L10a-3 | -Inf | Down |
| c187757_g4_i1 | ribosomal protein L12 | -Inf | Down |
| c185205_g4_i1 | ribosomal protein L15 | -Inf | Down |
| c295312_g1_i1 | ribosomal protein L16 | -3.5671 | Down |
| c360831_g1_i1 | ribosomal protein L18 | -6.3061 | Down |
| c116689_g1_i1 | ribosomal protein L18-1 | -Inf | Down |
| c200358_g2_i2 | ribosomal protein L19-1 | 1.392 | up |
| c24772_g1_i1 | ribosomal protein L2 | -6.6885 | Down |
| c197072_g1_i2 | ribosomal protein L2 (rpl2) | Inf | up |
| c182334_g1_i1 | ribosomal protein L21-1 | -3.3867 | Down |
| c157489_g1_i1 | ribosomal protein L22 | -4.1635 | Down |
| c190730_g2_i1 | ribosomal protein L22 | -3.2719 | Down |
| c153349_g1_i1 | ribosomal protein L23a | -Inf | Down |
| c56714_g2_i1 | ribosomal protein L23a pseudogene | -Inf | Down |
| c356988_g1_i1 | ribosomal protein L24 | -3.6146 | down |
| c134556_g1_i1 | ribosomal protein L26 | -2.8011 | Down |
| c140608_g1_i1 | ribosomal protein L26-1-like | -6.57 | down |
| c115297_g1_i1 | ribosomal protein L27 | -Inf | Down |
| c240745_g1_i1 | ribosomal protein L27 | -3.4381 | Down |
| c228729_g1_i1 | ribosomal protein L27 (rpl27) | -6.1562 | Down |
| c151097_g1_i1 | ribosomal protein L3 | -5.1723 | Down |
| c151097_g2_i2 | ribosomal protein L3 | -Inf | Down |
| c330767_g1_i1 | ribosomal protein L31 | -2.8963 | down |
| c106247_g1_i1 | ribosomal protein L32 | -Inf | Down |
| c176138_g1_i1 | ribosomal protein L32 | -3.5009 | Down |
| c163198_g2_i1 | ribosomal protein L34 | -5.6536 | Down |
| c260246_g1_i1 | ribosomal protein L34 | -3.1866 | Down |
| c176368_g1_i1 | ribosomal protein L34e | -4.7126 | Down |
| c231788_g1_i1 | ribosomal protein L37 | -Inf | Down |
| c176147_g1_i1 | ribosomal protein L37A | -Inf | Down |
| c176464_g1_i1 | ribosomal protein L37a protein partial | -Inf | Down |
| c126394_g1_i1 | ribosomal protein L38 | -3.4271 | Down |
| c183271_g1_i1 | ribosomal protein L3-like | -6.4626 | Down |
| c196239_g2_i1 | ribosomal protein L4 | -3.556 | Down |
| c168234_g1_i1 | ribosomal protein L4 (RPL4) | -5.3639 | Down |
| c151226_g1_i1 | ribosomal protein L4-1 | -5.4541 | Down |
| c176147_g1_i4 | ribosomal protein L43 | -Inf | Down |
| c160528_g1_i1 | ribosomal protein L5e | -Inf | Down |
| c22301_g1_i1 | ribosomal protein L6 | -3.8236 | Down |
| c164035_g2_i1 | ribosomal protein L7 | -Inf | Down |
| c30003_g1_i1 | ribosomal protein L7 (rpl7 gene) | -4.168 | Down |
| c116977_g1_i1 | ribosomal protein L7Ae | -4.2824 | Down |
| c164295_g2_i1 | ribosomal protein L8 | -Inf | Down |
| c188196_g1_i1 | ribosomal protein L9 | -Inf | Down |
| c182301_g3_i2 | ribosomal protein L9 | -Inf | Down |
| c140592_g1_i1 | ribosomal protein LP0 | -Inf | Down |
| c164371_g3_i1 | ribosomal protein P1 | -4.1352 | Down |
| c196322_g3_i1 | ribosomal protein P1 | -3.3783 | Down |
| c164371_g2_i1 | ribosomal protein P2 | -Inf | Down |
| c202045_g1_i6 | ribosomal protein RPL10A partial | -4.1321 | Down |
| c159037_g1_i1 | ribosomal protein rps3a | -Inf | Down |
| c199530_g1_i5 | ribosomal protein S0 | -Inf | Down |
| c162152_g2_i1 | ribosomal protein S10 | -2.8217 | Down |
| c182124_g1_i1 | ribosomal protein S11 (rps11) | -Inf | Down |
| c122043_g1_i1 | ribosomal protein S12 | -3.4578 | Down |
| c190811_g1_i1 | ribosomal protein S13 | -Inf | Down |
| c229072_g1_i1 | ribosomal protein S13 | -3.4102 | down |
| c47319_g1_i1 | ribosomal protein S13 | -Inf | Down |
| c183904_g1_i1 | ribosomal protein S14 | -3.2828 | Down |
| c185143_g1_i1 | ribosomal protein S14 | -Inf | Down |
| c266534_g1_i1 | ribosomal protein S15 | -5.2148 | Down |
| c146088_g1_i1 | ribosomal protein S15a | -Inf | Down |
| c205236_g1_i2 | ribosomal protein S15ab | 1.8067 | up |
| c260883_g1_i1 | ribosomal protein S18 | -3.3942 | Down |
| c98972_g1_i1 | ribosomal protein S20-1 | -5.6334 | Down |
| c106013_g1_i1 | ribosomal protein S21 (CRP7) | -3.4031 | Down |
| c163396_g1_i1 | ribosomal protein S23 | -6.0005 | Down |
| c159047_g1_i2 | ribosomal protein S24 | -Inf | Down |
| c99794_g1_i1 | ribosomal protein S24-1-like | -3.1729 | Down |
| c158359_g1_i2 | ribosomal protein S25 | -Inf | Down |
| c171600_g1_i1 | ribosomal protein S25 | -3.6679 | Down |
| c155741_g2_i1 | ribosomal protein S26 | -3.1058 | Down |
| c133235_g1_i1 | ribosomal protein S27 | -Inf | Down |
| c361255_g1_i1 | ribosomal protein S28e | -3.6678 | Down |
| c230516_g1_i1 | ribosomal protein S3 | -6.3015 | Down |
| c179696_g1_i4 | ribosomal protein S30 | -2.7585 | Down |
| c133582_g1_i1 | ribosomal protein S3-3 | -4.3338 | Down |
| c177215_g1_i2 | ribosomal protein S3a-2 | -Inf | Down |
| c169893_g3_i1 | ribosomal protein S3Ae | -3.5346 | Down |
| c157725_g1_i1 | ribosomal protein S4 | -6.5026 | Down |
| c8330_g1_i1 | ribosomal protein S4 | -3.7124 | Down |
| c165704_g1_i2 | ribosomal protein S4 (RpS4) | -Inf | Down |
| c194927_g5_i3 | ribosomal protein S5 | -4.1047 | Down |
| c184072_g4_i1 | ribosomal protein S5 | -3.2237 | Down |
| c126706_g1_i2 | ribosomal protein S6 | -6.5846 | Down |
| c195050_g1_i3 | ribosomal protein S7 | -3.8805 | Down |
| c177166_g1_i1 | ribosomal protein S7 | -3.2755 | Down |
| c171504_g1_i1 | ribosomal protein S8 | -2.8323 | Down |
| c78326_g1_i1 | ribosomal protein S8 | -Inf | Down |
| c357223_g1_i1 | ribosomal protein S9 | -4.48 | Down |
| c81452_g1_i1 | Ribosomal protein S9 | -5.6724 | Down |
| c98443_g1_i1 | ribosomal protein SA | -4.2047 | Down |
| c12157_g1_i1 | ribosomal RNA gene | -2.7594 | Down |
| c146839_g1_i1 | ribosomal RNA gene | -3.2546 | Down |
| c174872_g2_i1 | ribosomal RNA gene | -3.0611 | Down |
| c178682_g1_i2 | ribosomal RNA gene | -Inf | Down |
| c184459_g8_i1 | ribosomal RNA gene | -Inf | Down |
| c204202_g1_i1 | ribosomal RNA small subunit methyltransferase | -1.785 | Down |
| c196686_g1_i5 | RING-H2 finger protein ATL20 | -1.1792 | Down |
| c200892_g2_i1 | RING-H2 finger protein ATL2-like | 1.0668 | up |
| c192969_g1_i1 | RING-H2 finger protein ATL40-like | -3.7469 | Down |
| c179436_g1_i1 | RING-H2 finger protein ATL60 | 2.9304 | up |
| c188467_g1_i1 | RING-H2 finger protein ATL79 | -1.5265 | Down |
| c183382_g1_i3 | RING-H2 finger protein ATL80-like | 2.3866 | up |
| c161313_g1_i2 | rotein | 1.3728 | up |
| c194591_g2_i1 | RPM1-interacting protein 4 | 3.2836 | up |
| c194591_g2_i2 | RPM1-interacting protein 4 | 3.5464 | up |
| c190618_g1_i1 | RPM1-interacting protein 4-like | Inf | up |
| c195171_g3_i1 | rRNA (uridine-2'-O-)-methyltransferase | -4.3324 | Down |
| c202432_g1_i3 | saccharopine dehydrogenase | -5.9737 | Down |
| c359044_g1_i1 | S-adenosylmethionine synthase 1 | -6.4628 | Down |
| c198471_g3_i1 | S-adenosyl-methionine synthetase gene | -Inf | down |
| c194923_g1_i1 | S-adenosylmethionine-dependent methyltransferase | 2.1316 | up |
| c174147_g2_i1 | SATB homeobox 1 (satb1) | -Inf | Down |
| c201007_g1_i1 | scarecrow-like protein | 1.5419 | up |
| c204702_g1_i5 | scarecrow-like protein | 1.1158 | up |
| c204455_g1_i4 | scarecrow-like protein 1 | 1.2614 | up |
| c199664_g2_i1 | scarecrow-like protein 11 | 1.7715 | up |
| c197439_g1_i1 | scarecrow-like protein 3 | -1.5753 | Down |
| c204702_g1_i2 | scarecrow-like protein 30 | 2.1805 | up |
| c204702_g1_i3 | scarecrow-like protein 30 | 1.4408 | up |
| c204702_g1_i1 | scarecrow-like protein 30 | 1.3426 | up |
| c202435_g2_i4 | scarecrow-like protein 31 | 1.1431 | up |
| c205820_g2_i1 | scarecrow-like protein 34 | 1.4416 | up |
| c194121_g1_i1 | scarecrow-like protein 8 | 1.0028 | up |
| c205644_g1_i1 | scarecrow-like transcription factor PAT1 | 2.5302 | up |
| c198983_g1_i1 | SEC12-like protein 1 | -1.4462 | down |
| c205127_g1_i4 | SEC14 cytosolic factor | 2.5878 | up |
| c205127_g1_i5 | SEC14 cytosolic factor-like | -1.4816 | Down |
| c204489_g1_i3 | Sec23 homolog | -2.2 | Down |
| c202216_g1_i1 | senescence related protein | 2.3065 | up |
| c202216_g1_i2 | senescence related protein | 3.2755 | up |
| c202216_g1_i3 | senescence related protein | 2.354 | up |
| c202270_g5_i1 | senescence related protein | 3.3343 | up |
| c202270_g5_i4 | senescence related protein | 3.4488 | up |
| c205614_g1_i3 | senescence-associated carboxylesterase 101 | 2.1739 | up |
| c191638_g3_i16 | senescence-associated carboxylesterase 101-like | 4.6644 | up |
| c191638_g3_i8 | senescence-associated carboxylesterase 101-like | Inf | up |
| c163541_g1_i1 | sequestosome-1 | -3.6215 | Down |
| c200083_g1_i1 | serine acetyltransferase 1 | 1.1354 | up |
| c197595_g1_i1 | serine carboxypeptidase-like 29 | 1.1497 | up |
| c198139_g1_i1 | serine carboxypeptidase-like 34 | -Inf | Down |
| c202654_g1_i3 | serine carboxypeptidase-like 35 | 1.2277 | up |
| c197053_g2_i1 | serine dehydratase-like (SDSL) | -3.3493 | Down |
| c201565_g2_i1 | serine hydroxymethyltransferase | -5.4343 | Down |
| c197361_g1_i2 | serine threonine-protein kinase | 1.271 | up |
| c197361_g1_i3 | serine threonine-protein kinase | 1.5969 | up |
| c199197_g1_i2 | serine threonine-protein kinase | 2.294 | up |
| c199259_g2_i3 | serine threonine-protein kinase NAK | 1.0867 | up |
| c199197_g1_i1 | Serine/threonine-protein kinase | 1.4248 | up |
| c200645_g1_i3 | serine/threonine-protein kinase | 1.5949 | up |
| c200128_g1_i3 | Serine/threonine-protein kinase | 1.1762 | up |
| c175896_g1_i1 | serinearginine repetitive matrix protein 2-like | -3.4655 | Down |
| c196844_g1_i5 | serinearginine-rich-splicing factor SR34 | -Inf | Down |
| c206065_g1_i1 | serine-rich adhesin for platelets | 1.6335 | up |
| c203792_g1_i3 | serinethreonine protein kinase | 2.6641 | up |
| c203792_g1_i6 | serinethreonine protein kinase | 3.5814 | up |
| c196934_g1_i9 | serinethreonine protein kinase | 1.8254 | up |
| c196934_g1_i10 | serinethreonine protein kinase | 1.7838 | up |
| c190815_g1_i2 | serinethreonine-protein kinase | 3.5314 | up |
| c203200_g1_i2 | serinethreonine-protein kinase | 2.9491 | up |
| c203364_g1_i7 | serinethreonine-protein kinase | 1.6803 | up |
| c203659_g1_i1 | serinethreonine-protein kinase | 2.2058 | up |
| c202673_g2_i3 | serinethreonine-protein kinase | 3.4184 | up |
| c179129_g4_i1 | serinethreonine-protein kinase | -Inf | Down |
| c205819_g2_i1 | serinethreonine-protein kinase | 1.7599 | up |
| c200645_g1_i1 | serinethreonine-protein kinase | 1.5669 | up |
| c205819_g2_i7 | serinethreonine-protein kinase | 3.1804 | up |
| c200802_g1_i2 | serinethreonine-protein kinase AtPK1AtPK6-like | -Inf | Down |
| c203856_g1_i4 | serinethreonine-protein kinase Cx32 | 1.2434 | up |
| c177376_g1_i1 | serinethreonine-protein kinase minibrain | -Inf | Down |
| c202633_g1_i2 | serinethreonine-protein kinase NAK | 1.5811 | up |
| c202633_g1_i4 | serinethreonine-protein kinase NAK | 1.6029 | up |
| c196596_g1_i1 | serinethreonine-protein kinase rio2-like | 1.3922 | up |
| c200732_g2_i3 | serinethreonine-protein kinase WNK11 | 2.0249 | up |
| c200898_g1_i1 | serinethreonine-protein kinase WNK8 | -1.2858 | Down |
| c205141_g1_i2 | serinethreonine-protein kinase-like protein CCR1 | -1.2898 | Down |
| c205156_g1_i1 | serinethreonine-protein kinase-like protein CCR3 | 1.866 | up |
| c203058_g3_i4 | serinethreonine-protein phosphatase PP1 isozyme 9 | 1.6479 | up |
| c203404_g2_i1 | serine--tRNA ligase | -4.5586 | Down |
| c193469_g1_i1 | serpin-ZX | 1.6752 | up |
| c204277_g1_i3 | SH2-domain binding protein 1 (shcbp1) | -4.1266 | Down |
| c199084_g1_i2 | shaggy-related protein kinase epsilon- | 1.0249 | up |
| c196142_g7_i1 | Shattering4 gene locus, complete sequence | -5.0693 | Down |
| c203651_g1_i2 | shikimate O-hydroxycinnamoyltransferase | 1.4012 | up |
| c166269_g1_i1 | sigma factor binding protein 1 | 3.5523 | up |
| c203236_g2_i3 | signal peptide peptidase-like 4 | -2.7828 | Down |
| c204370_g1_i1 | signal recognition particle receptor subunit alpha | 1.4253 | up |
| c154159_g1_i2 | silicon transporter (SIT2) gene | -Inf | Down |
| c175782_g2_i1 | small nuclear ribonucleoprotein-associated protein B-like | 3.3815 | up |
| c205211_g1_i6 | smr (Small MutS Related) domain-containing protein | 1.3136 | up |
| c55358_g1_i1 | SN15 protein | -Inf | Down |
| c204660_g1_i5 | SNAP25 homologous protein SNAP33 | 1.5693 | up |
| c204660_g1_i9 | SNAP25 homologous protein SNAP33 | 1.1028 | up |
| c206526_g2_i1 | SNc domain-containing protein, mRNA | -Inf | Down |
| c204430_g2_i1 | SNF-related serinethreonine-protein kinase | -4.1067 | Down |
| c205408_g1_i1 | Sodiumcalcium exchanger family protein calcium-binding EF hand family protein | 1.9841 | up |
| c196850_g1_i2 | soluble inorganic pyrophosphatase-like | -4.8123 | Down |
| c165082_g1_i1 | solute carrier family 11 member 1 protein | -3.6869 | Down |
| c188730_g4_i1 | solute carrier family 25 | -5.7387 | Down |
| c195264_g1_i8 | somatic embryogenesis receptor kinase 1-like | 1.8122 | up |
| c195264_g1_i7 | somatic embryogenesis receptor kinase 2-like | 4.9975 | up |
| c177294_g1_i2 | sorbitol dehydrogenase protein | -3.409 | Down |
| c150068_g2_i1 | spectrin, beta, erythrocytic | -4.9286 | Down |
| c206205_g1_i2 | spermatogenesis-associated protein 20 | -2.1019 | Down |
| c190706_g1_i3 | S-phase kinase-associated protein | -Inf | Down |
| c200606_g2_i1 | sphinganine C(4)-monooxygenase 1 | 1.5165 | up |
| c181932_g2_i1 | splicing factor 3A subunit 2-like | 1.3692 | up |
| c136265_g1_i1 | sprouty-related, EVH1 domain containing 2 | -3.2243 | Down |
| c172200_g2_i1 | sprouty-related, EVH1 domain-containing protein 1 | -1.398 | Down |
| c168922_g1_i2 | SRSF protein kinase 3 | -Inf | Down |
| c150765_g1_i1 | STE like transcription facto | -Inf | Down |
| c185807_g1_i1 | stem-specific protein TSJT1-like | 1.0465 | up |
| c169049_g1_i1 | sterile alpha motif domain containing 9 (Samd9) | -Inf | Down |
| c196963_g1_i4 | steroid 5-alpha-reductase DET2 | 1.7744 | up |
| c187556_g1_i1 | steroid membrane binding protein | -4.9233 | Down |
| c201142_g1_i2 | sterol desaturase | Inf | up |
| c178363_g1_i1 | stomatin family protein | -2.4038 | Down |
| c200265_g2_i3 | stress response protein NST1-like | -2.0193 | Down |
| c200265_g2_i5 | stress response protein NST1-like | -1.3778 | Down |
| c178235_g1_i1 | stress-induced protein KIN2 | 4.2386 | up |
| c200082_g1_i2 | substrate carrier family protein B | 2.7455 | up |
| c200082_g1_i4 | substrate carrier family protein B | 3.185 | up |
| c206119_g1_i2 | succinate dehydrogenase, mRNA | -5.2956 | Down |
| c206119_g1_i4 | succinate dehydrogenase, mRNA | -Inf | Down |
| c193894_g1_i3 | sufE-like protein 2 | 1.5365 | up |
| c198359_g1_i1 | sugar phosphate phosphate translocator | 2.2216 | up |
| c198859_g1_i1 | sugar transport protein 4 | -1.2204 | down |
| c197650_g1_i3 | sugar transporter ERD6-like 12 | Inf | up |
| c197650_g1_i5 | sugar transporter ERD6-like 12 | Inf | up |
| c195219_g1_i13 | sugar transporter ERD6-like 15 | 1.4751 | up |
| c195823_g1_i1 | sugar transporter ERD6-like 6 | -1.264 | Down |
| c202502_g1_i1 | superoxide-generating NADPH oxidase flavocytochrome | -Inf | Down |
| c177499_g1_i1 | surface glycoprotein gene | -Inf | Down |
| c195644_g2_i7 | surfeit locus protein 2 | -Inf | Down |
| c197651_g1_i2 | TATA element modulatory factor 1 (TMF1), | -Inf | Down |
| c205487_g1_i2 | TBC1 domain family member 15 | 1.0475 | up |
| c205487_g1_i5 | TBC1 domain family member 15 | 1.0412 | up |
| c205487_g1_i6 | TBC1 domain family member 15 | 1.8604 | up |
| c95676_g2_i1 | T-complex protein 1 | -5.3654 | Down |
| c203831_g1_i3 | T-complex protein 1 subunit epsilon | -5.6578 | Down |
| c186949_g4_i1 | T-complex protein 1 subunit eta-like | -Inf | Down |
| c203745_g1_i2 | T-complex protein 1 subunit gamma | -4.9835 | Down |
| c203745_g1_i1 | T-complex protein 1 subunit gamma | -5.477 | Down |
| c202308_g1_i4 | telomere repeat-binding protein 1 | 1.1942 | up |
| c17135_g1_i1 | tenascin-like | -4.5317 | Down |
| c189802_g1_i1 | tetraspanin-3 | 1.2076 | up |
| c183358_g1_i1 | tetraspanin-8 | 2.1201 | up |
| c195892_g1_i4 | tetratricopeptide repeat domain 37 | -4.463 | Down |
| c204374_g1_i5 | tetratricopeptide repeat protein 38 | -1.0373 | Down |
| c195534_g1_i6 | thaumatin-like protein 1b | 2.2362 | up |
| c199757_g1_i4 | thaumatin-like protein 1b | 2.7677 | up |
| c181221_g1_i1 | thioredoxin | -3.9363 | Down |
| c203459_g1_i1 | thioredoxin reductase | -4.3188 | Down |
| c202335_g2_i1 | thioredoxin-like 2-2 | 1.4897 | up |
| c187602_g1_i1 | thylakoidal processing peptidase 1 | 1.1621 | up |
| c193170_g1_i1 | thylakoidal processing peptidase 2 | 1.1269 | up |
| c193170_g2_i2 | thylakoidal processing peptidase 2 | 1.195 | up |
| c110187_g1_i1 | thyroid hormone receptor interactor 12 | -Inf | Down |
| c192091_g1_i2 | TIFY5B | 3.8254 | up |
| c180960_g1_i1 | TIFY5B | 5.5046 | up |
| c180960_g1_i2 | TIFY5B | 5.7918 | up |
| c194467_g2_i1 | TMV resistance protein N-like | 4.5082 | up |
| c202225_g1_i9 | Tol A like protein | -5.9239 | Down |
| c194467_g1_i1 | Toll-Interleukin-Resistance domain-containing protein | 2.7826 | up |
| c206544_g2_i3 | TRAF3-interacting protein 1 | -Inf | Down |
| c201300_g1_i2 | transcription factor ABA-INDUCIBLE bHLH-TYPE | 1.9267 | up |
| c201300_g1_i3 | transcription factor ABA-INDUCIBLE bHLH-TYPE | 2.6687 | up |
| c198607_g1_i1 | transcription factor bHLH129-like | -1.5957 | Down |
| c198607_g1_i2 | transcription factor bHLH129-like | -2.069 | Down |
| c198607_g1_i4 | transcription factor bHLH129-like | -2.7715 | Down |
| c197125_g1_i3 | transcription factor bHLH130-like | -1.265 | Down |
| c196938_g1_i1 | transcription factor bHLH19-like | 3.2069 | up |
| c196938_g3_i6 | transcription factor bHLH19-like | Inf | up |
| c201750_g1_i1 | transcription factor bHLH27 | 2.5244 | up |
| c198300_g1_i4 | transcription factor bHLH35 | 1.963 | up |
| c202669_g1_i3 | transcription factor bHLH78 | -1.027 | Down |
| c195186_g1_i1 | transcription factor bHLH92 | 4.7306 | up |
| c195186_g1_i3 | transcription factor bHLH92 | 5.1794 | up |
| c179885_g1_i1 | transcription factor binding to IGHM enhancer 3 (TFE3) | -Inf | Down |
| c195377_g1_i2 | transcription factor HBI1-like | -2.4531 | Down |
| c195377_g1_i3 | transcription factor HBI1-like | -1.9526 | Down |
| c195377_g1_i5 | transcription factor HBI1-like | -1.8805 | Down |
| c204685_g2_i1 | transcription factor HBP-1b | -5.2092 | Down |
| c199761_g1_i1 | transcription factor MYB108 | 2.4225 | up |
| c199761_g1_i7 | transcription factor MYB108 | 1.7865 | up |
| c199761_g1_i4 | transcription factor MYB108 | 2.1121 | up |
| c199761_g1_i2 | transcription factor MYB108-like | 1.9827 | up |
| c199761_g1_i5 | transcription factor MYB108-like | 2.2493 | up |
| c186481_g4_i1 | transcription factor MYB24 | 2.7856 | up |
| c199317_g1_i3 | transcription factor MYB3-like | -1.5394 | Down |
| c187573_g1_i2 | transcription factor MYB44-like | 1.5169 | up |
| c186481_g2_i2 | transcription factor MYB57 | 2.585 | up |
| c194244_g1_i1 | transcription factor MYB59 | -1.1102 | down |
| c187590_g3_i3 | transcription factor MYC3-like | Inf | up |
| c198082_g1_i8 | transcription factor PIF3-like | 1.5046 | up |
| c194257_g1_i1 | transcription factor PosF21 | 1.4822 | up |
| c203998_g2_i2 | transcription factor PosF21 | 2.0588 | up |
| c194754_g2_i4 | transcription factor RAX2-like | -1.7092 | Down |
| c156302_g1_i1 | transcription factor SOX-14 | -Inf | Down |
| c200916_g1_i12 | transcription initiation factor IIB | 2.6367 | up |
| c200916_g1_i4 | transcription initiation factor IIB-1 | Inf | up |
| c199647_g2_i2 | transcription initiation factor TFIID subunit 3 | 1.4448 | up |
| c197550_g2_i2 | transcription repressor MYB4 | -3.4693 | Down |
| c197550_g1_i1 | transcription repressor MYB4-like | -2.0017 | Down |
| c202386_g1_i1 | transcription repressor MYB6 | -1.2528 | down |
| c199761_g3_i2 | transcriptional corepressor SEUSS | -3.2271 | Down |
| c187563_g10_i2 | translation elongation factor 1 alpha (TEF1) gene | -Inf | Down |
| c172459_g1_i1 | translation elongation factor 2 Eft2 | -Inf | Down |
| c193382_g4_i1 | translation elongation factor IF5A | -4.4925 | down |
| c203109_g2_i1 | translation initiation factor | -5.2515 | down |
| c36638_g1_i1 | translation initiation factor 1A | -3.8552 | down |
| c181670_g1_i2 | translation initiation factor eIF-5A family protein | -4.4381 | Down |
| c160205_g1_i1 | translation initiation factor IF-3 | -Inf | Down |
| c163267_g1_i2 | translocation associated membrane protein 1 (TRAM1) | -Inf | Down |
| c203664_g1_i2 | transmembrane protein 184 homolog DDB_ | 1.0303 | up |
| c204706_g1_i1 | transmembrane protein 194A-like | 2.1106 | up |
| c204706_g1_i2 | transmembrane protein 194A-like | 2.1604 | up |
| c204706_g1_i5 | transmembrane protein 194A-like | 2.2598 | up |
| c200500_g1_i1 | transmembrane protein 53 | 2.5005 | up |
| c200500_g1_i6 | transmembrane protein 53 | 3.5078 | up |
| c200500_g1_i2 | transmembrane protein 53 | 2.5505 | up |
| c200500_g1_i3 | transmembrane protein 53 | 3.2732 | up |
| c204714_g1_i2 | transporter YBR287W | 1.0486 | up |
| c204669_g1_i1 | trans-resveratrol di-O-methyltransferase | 3.4021 | up |
| c204669_g1_i2 | trans-resveratrol di-O-methyltransferase | 3.563 | up |
| c203945_g1_i5 | trehalase | -1.5571 | Down |
| c189109_g3_i4 | trehalose-phosphate phosphatase | Inf | up |
| c190065_g1_i3 | trehalose-phosphate phosphatase A-like | 3.0556 | up |
| c190065_g1_i4 | trehalose-phosphate phosphatase A-like | Inf | up |
| c190065_g1_i7 | trehalose-phosphate phosphatase A-like | 2.0663 | up |
| c189109_g1_i1 | trehalose-phosphate phosphatase I | 5.1469 | up |
| c189109_g3_i1 | trehalose-phosphate phosphatase I | Inf | up |
| c203166_g3_i2 | tricalbin-3 | Inf | up |
| c204925_g1_i2 | trichome birefringence | 1.7387 | up |
| c203760_g1_i7 | trihelix transcription factor GT-2 | -1.2642 | Down |
| c196446_g1_i4 | trihelix transcription factor GTL1-like | -1.107 | Down |
| c108477_g1_i1 | tripeptidyl peptidase | -Inf | Down |
| c165176_g1_i1 | tripeptidyl peptidase 1 precursor protein | -5.1077 | Down |
| c203084_g3_i3 | tRNA (uracil-5-)-methyltransferase homolog A | 1.6978 | up |
| c198673_g5_i1 | trnH-psbA intergenic spacer | -3.6593 | Down |
| c183390_g1_i1 | tropinone reductase homolog | 1.5742 | up |
| c192306_g1_i1 | trypsin inhibitor BvTI-like | 1.0953 | up |
| c201013_g3_i1 | tryptophan--tRNA ligase | -4.7967 | Down |
| c204424_g1_i1 | tubby-like F-box protein 1 | 1.9002 | up |
| c140559_g1_i1 | tubulin polyglutamylase TTLL7 | -Inf | Down |
| c199762_g1_i2 | two-component response regulator ARR5 | 1.6362 | up |
| c199762_g1_i1 | two-component response regulator ARR5 | 2.1302 | up |
| c199762_g1_i3 | two-component response regulator ARR5 | 2.3339 | up |
| c199762_g1_i4 | two-component response regulator ARR5 | 2.6823 | up |
| c198177_g3_i1 | two-component response regulator ARR7 | 1.53 | up |
| c198177_g2_i1 | two-component response regulator ARR7-like | 1.6238 | up |
| c198177_g2_i2 | two-component response regulator ARR7-like | 1.9799 | up |
| c190691_g1_i4 | two-component response regulator ARR8-like | -2.5506 | Down |
| c204316_g3_i3 | two-component response regulator ARR9 | -1.9424 | Down |
| c201833_g2_i1 | two-component response regulator-like APRR2 | 1.1888 | up |
| c195114_g1_i1 | tyrosine aminotransferase-like | 1.2037 | up |
| c202514_g1_i1 | tyrosine-protein kinase transmembrane receptor Ror2 | -2.2645 | Down |
| c202603_g2_i1 | tyrosyl-tRNA synthetase, mRNA | -4.7868 | Down |
| c204957_g2_i1 | U3 snoRNP protein | -4.3274 | Down |
| c194515_g1_i1 | ubiquinol oxidase 1a, mitochondrial-like | 1.508 | up |
| c159987_g2_i2 | ubiquinol oxidase 3 | 2.1865 | up |
| c159987_g2_i1 | ubiquinol oxidase3 | 2.1234 | up |
| c202912_g1_i4 | ubiquinol-cytochrome c oxidoreductase complex subunit | -3.8629 | Down |
| c201648_g4_i2 | ubiquitin carboxyl-terminal hydrolase 27 | Inf | up |
| c203062_g1_i1 | ubiquitin carboxyl-terminal peptidase | -Inf | Down |
| c199549_g1_i3 | ubiquitin domain-containing protein DSK2b-like | -Inf | Down |
| c119507_g1_i1 | ubiquitin-40S ribosomal protein | -4.0946 | Down |
| c197351_g1_i16 | ubiquitin-40S ribosomal protein S27a-2 | -3.2042 | Down |
| c193931_g1_i1 | ubiquitin-conjugating enzyme | -Inf | Down |
| c200078_g2_i1 | ubiquitin-conjugating enzyme E2 30 | 1.1123 | up |
| c171546_g2_i1 | ubiquitin-like modifier activating enzyme 1 (UBA1) | -5.3324 | Down |
| c188027_g1_i3 | ubiquitin-like-specific protease 1B | -Inf | Down |
| c201981_g2_i1 | U-box domain-containing protein 17 | 1.2755 | up |
| c204732_g1_i1 | U-box domain-containing protein 17 | 1.8673 | up |
| c205781_g1_i1 | U-box domain-containing protein 2 | 1.7098 | up |
| c205781_g1_i3 | U-box domain-containing protein 2 | 1.8952 | up |
| c196795_g1_i1 | U-box domain-containing protein 21-like | 1.3712 | up |
| c201539_g1_i5 | U-box domain-containing protein 25 | 2.0163 | up |
| c197800_g1_i1 | U-box domain-containing protein 31 | 1.2845 | up |
| c189467_g1_i2 | U-box domain-containing protein 39-like | 1.6947 | up |
| c202114_g1_i6 | U-box domain-containing protein 5 | 2.0125 | up |
| c198065_g1_i7 | U-box domain-containing protein 6-like | 3.2227 | up |
| c205863_g1_i2 | U-box domain-containing protein 9 | 1.6173 | up |
| c205863_g1_i3 | U-box domain-containing protein 9 | 1.5083 | up |
| c202818_g2_i1 | UDP-arabinose 4-epimerase 1 | 1.346 | up |
| c199783_g1_i4 | UDP-galactose transporter 2 | 1.8993 | up |
| c199783_g1_i5 | UDP-galactose transporter 2 | 1.8642 | up |
| c199611_g1_i2 | UDP-galactose transporter 2-like | -Inf | Down |
| c198306_g1_i5 | UDP-galactose UDP glucose transporter 2-like | 1.2573 | up |
| c202561_g1_i2 | UDP-glucuronate 4-epimerase 1 | 1.6776 | up |
| c200189_g3_i1 | UDP-glucuronate 4-epimerase 3-like | -Inf | Down |
| c195378_g1_i3 | UDP-glycosyltransferase 72E2 | -2.0095 | down |
| c195907_g1_i1 | UDP-glycosyltransferase 72E2 | -2.2103 | down |
| c195593_g1_i1 | UDP-glycosyltransferase 73B3-like | 1.1131 | up |
| c203440_g1_i1 | UDP-glycosyltransferase 73D1 | 1.2721 | up |
| c195714_g2_i2 | UDP-glycosyltransferase 74C1-like | 1.3372 | up |
| c196720_g1_i1 | UDP-glycosyltransferase 74F2-like | 1.2869 | up |
| c201731_g2_i1 | UDP-glycosyltransferase 89B1 | 3.2365 | up |
| c201822_g3_i2 | UDP-glycosyltransferase 91B1-like | -Inf | Down |
| c182999_g1_i1 | UDP-glycosyltransferase 91C1-like | -1.1939 | Down |
| c198879_g1_i1 | universal stress protein A-like protein | 1.0597 | up |
| c203015_g2_i7 | UPF0187 protein | Inf | up |
| c202436_g1_i1 | UPF0481 protein | 1.4432 | up |
| c194756_g1_i5 | UPF0496 protein | 3.6103 | up |
| c203939_g1_i1 | UPF0496 protein | 3.8653 | up |
| c204293_g1_i2 | UV radiation resistance associated protein | -3.6289 | Down |
| c203571_g1_i2 | vacuolar amino acid transporter 1 | 1.7774 | up |
| c157267_g1_i1 | vacuolar ATP synthase 16 kDa proteolipid subunit | -4.6122 | down |
| c200946_g4_i1 | vacuolar ATP synthase 16 kDa proteolipid subunit | -3.2703 | down |
| c203764_g1_i1 | vacuolar cationproton exchanger 1 | 1.5402 | up |
| c183144_g1_i1 | vacuolar cationproton exchanger 2 | -2.5296 | down |
| c188545_g1_i3 | Vacuolar H^+^ATPase subunit C | -4.9968 | down |
| c190414_g1_i1 | vacuolar protein sorting-associated protein 2 homolog 3 | 1.1938 | up |
| c184946_g3_i1 | vacuolar-processing enzyme alpha-isozyme | 2.5072 | up |
| c198782_g1_i1 | vacuolar-processing enzyme beta-isozyme- | 1.8098 | up |
| c181306_g1_i3 | vacuolar-type H+-ATPase subunit A | -4.182 | down |
| c180498_g1_i2 | valosin containing protein (VCP) | -6.7282 | Down |
| c207463_g1_i2 | VARIATION IN COMPOUND TRIGGERED ROOT growth response | 1.6215 | up |
| c104978_g1_i1 | V-ATPase A subunit | -Inf | Down |
| c196963_g1_i2 | very-long-chain enoyl-CoA reductase-like | 1.916 | up |
| c197377_g1_i3 | vesicle transport protein USE1-like | -Inf | Down |
| c196076_g1_i2 | voltage-gated potassium channel subunit beta | -Inf | Down |
| c180325_g1_i2 | VQ motif-containing protein 10 | 1.8322 | up |
| c181306_g3_i1 | V-type proton ATPase catalytic subunit A | -Inf | down |
| c177234_g1_i7 | V-type proton ATPase subunit B3 | -Inf | Down |
| c203355_g1_i2 | wall associated kinase | -1.5886 | Down |
| c203916_g1_i2 | wall-associated receptor kinase | 2.4649 | up |
| c203916_g1_i4 | wall-associated receptor kinase | 1.4276 | up |
| c200396_g1_i3 | wall-associated receptor kinase | 2.7992 | up |
| c178201_g1_i1 | wall-associated receptor kinase-like 11 | 4.3094 | up |
| c190923_g3_i2 | wall-associated receptor kinase-like 2 | 2.9983 | up |
| c190923_g4_i2 | wall-associated receptor kinase-like 2 | 2.9841 | up |
| c192115_g5_i1 | WAT1-related protein | 1.8749 | up |
| c198945_g1_i1 | WAT1-related protein | -1.4536 | Down |
| c151168_g1_i1 | WD repeat-containing protein 1 | -6.0861 | Down |
| c206238_g1_i1 | WD repeat-containing protein 44 | 1.2405 | up |
| c198319_g1_i2 | WD repeat-containing protein 53 | Inf | up |
| c202835_g1_i3 | WD40 repeat domain-containing protein | -Inf | Down |
| c195379_g1_i4 | WEB family protein | 5.7808 | up |
| c196786_g1_i1 | willebrand factor type A (vWA) domain protein | -Inf | Down |
| c198132_g1_i1 | WPP domain-interacting protein 2 | 2.0209 | up |
| c205432_g1_i2 | WRKY transcription factor 18 | 1.6242 | up |
| c205432_g1_i3 | WRKY transcription factor 18 | 1.7374 | up |
| c205432_g1_i5 | WRKY transcription factor 18 | 1.6178 | up |
| c205432_g1_i7 | WRKY transcription factor 18 | 1.8246 | up |
| c178987_g1_i1 | WRKY transcription factor 18-like | 2.1996 | up |
| c178987_g1_i4 | WRKY transcription factor 18-like | 1.5981 | up |
| c191006_g1_i1 | WRKY transcription factor 18-like | 1.9762 | up |
| c174581_g1_i2 | WRKY transcription factor 20 | -Inf | Down |
| c194055_g2_i4 | WRKY transcription factor 26 | 1.2294 | up |
| c194692_g2_i1 | WRKY transcription factor 29 | -1.9217 | Down |
| c194692_g2_i3 | WRKY transcription factor 29 | -1.6104 | Down |
| c196038_g2_i2 | WRKY transcription factor 30 | 3.1461 | up |
| c196038_g2_i4 | WRKY transcription factor 30 | 3.609 | up |
| c182982_g1_i1 | WRKY transcription factor 33 | 1.6431 | up |
| c190819_g2_i1 | WRKY transcription factor 33 | 2.6684 | up |
| c190819_g2_i2 | WRKY transcription factor 33 | 2.3015 | up |
| c159092_g1_i1 | WRKY transcription factor 40 | 3.234 | up |
| c184558_g1_i1 | WRKY transcription factor 41 | 2.8173 | up |
| c203704_g2_i2 | WRKY transcription factor 46 | 1.8206 | up |
| c201621_g1_i1 | WRKY transcription factor 48 | 1.4327 | up |
| c195662_g1_i2 | WRKY transcription factor 55-like | Inf | up |
| c197362_g1_i3 | WRKY transcription factor 58 | -1.3811 | Down |
| c198323_g1_i1 | WRKY transcription factor 61 | -1.7965 | Down |
| c198323_g2_i1 | WRKY transcription factor 61 | -1.6134 | Down |
| c193885_g1_i1 | WRKY transcription factor 64 | 5.1897 | up |
| c196445_g1_i1 | WRKY transcription factor 65 | -1.3443 | Down |
| c169856_g2_i1 | WW domain containing E3 ubiquitin protein ligase 2 | -3.195 | Down |
| c189205_g2_i2 | Xenobiotic-transporting ATPase | 2.5129 | up |
| c179030_g1_i1 | Xenopus (Silurana) tropicalis ERO1 | -Inf | Down |
| c204433_g1_i2 | xyloglucan endotransglucosylasehydrolase protein 30 | 1.5566 | up |
| c195900_g4_i1 | xyloglucan galactosyltransferase KATAMARI1 homolog | 1.4861 | up |
| c200567_g2_i3 | xylosyltransferase 2 | 1.24 | up |
| c198269_g1_i2 | xylosyltransferase 2-like | 1.4194 | up |
| c198072_g1_i7 | zinc finger A20 and AN1 domain-containing stress-associated protein 6-like | 1.5752 | up |
| c198072_g1_i1 | zinc finger A20 and AN1 domain-containing stress-associated protein 6-like | 1.6026 | up |
| c198072_g1_i2 | zinc finger A20 and AN1 domain-containing stress-associated protein 6-like | 1.3233 | up |
| c198072_g1_i8 | zinc finger A20 and AN1 domain-containing stress-associated protein 6-like | 1.2085 | up |
| c189488_g3_i3 | zinc finger A20 and AN1 domain-containing stress-associated protein 9 | 1.2088 | up |
| c198633_g3_i2 | zinc finger BED domain-containing protein DAYSLEEPER-like | 2.4061 | up |
| c200974_g2_i1 | zinc finger CCCH domain-containing protein | 1.7277 | up |
| c204294_g2_i3 | zinc finger CCCH domain-containing protein | 1.9904 | up |
| c197395_g1_i2 | zinc finger CCCH domain-containing protein 15 homolog | -Inf | Down |
| c165066_g1_i2 | zinc finger CCCH domain-containing protein 18-like | -3.3442 | Down |
| c204294_g1_i2 | zinc finger CCCH domain-containing protein 26 | 1.7274 | up |
| c204294_g2_i1 | zinc finger CCCH domain-containing protein 26 | 1.8702 | up |
| c200974_g1_i1 | zinc finger CCCH domain-containing protein 29 | 1.6402 | up |
| c200974_g1_i2 | zinc finger CCCH domain-containing protein 29 | 1.5992 | up |
| c200974_g1_i3 | zinc finger CCCH domain-containing protein 29 | 1.7642 | up |
| c202176_g1_i4 | zinc finger CCCH domain-containing protein 29 | 1.7507 | up |
| c202176_g1_i1 | zinc finger CCCH domain-containing protein 29 | 1.6238 | up |
| c204670_g1_i1 | zinc finger CCCH domain-containing protein 29- | 2.0393 | up |
| c204670_g2_i1 | zinc finger CCCH domain-containing protein 29- | 1.9977 | up |
| c202220_g1_i1 | zinc finger CCCH domain-containing protein 44 | 1.6479 | up |
| c194622_g1_i4 | zinc finger CCCH domain-containing protein 65-like | 1.6559 | up |
| c194622_g1_i8 | zinc finger CCCH domain-containing protein 65-like | 4.6559 | up |
| c194622_g1_i1 | zinc finger CCCH domain-containing protein 65-like | 3.154 | up |
| c202058_g1_i1 | Zinc finger family protein | 1.5331 | up |
| c205043_g1_i8 | Zinc finger family protein, putative isoform 2 | -1.9345 | Down |
| c203197_g1_i1 | zinc finger protein 385B | -4.8823 | Down |
| c194637_g1_i1 | zinc finger protein 5-like | -1.0519 | Down |
| c157692_g1_i1 | zinc finger protein 840 | -Inf | Down |
| c192105_g3_i4 | zinc finger protein AZF2-like | 2.8441 | up |
| c192105_g3_i6 | zinc finger protein AZF2-like | 2.5942 | up |
| c199576_g1_i6 | zinc finger protein CONSTANS-LIKE 11 | -2.1372 | Down |
| c197590_g1_i4 | zinc finger protein CONSTANS-LIKE 16-like | -1.0561 | Down |
| c200156_g1_i4 | zinc finger protein CONSTANS-LIKE 6-like | -1.0892 | Down |
| c200156_g1_i6 | zinc finger protein CONSTANS-LIKE 6-like | -1.4635 | Down |
| c191893_g1_i2 | zinc finger protein DOF1.7 | 1.7359 | up |
| c188336_g1_i1 | zinc finger protein ZAT10-like | 1.6259 | up |
| c203618_g1_i2 | ZINC INDUCED FACILITATOR 1 | 1.3376 | up |
| c194194_g1_i4 | zingipain-2-like | 5.9756 | up |
| c200297_g1_i2 | zingipain-2-like | -Inf | Down |
