# Supplementary material for: Transcriptomic analysis of Eruca vesicaria subs. sativa lines with contrasting tolerance to polyethylene glycol-simulated drought stress
Source: BMC Plant Biol. 2019 Oct 11;19:419. doi: 10.1186/s12870-019-1997-2 (PMC6787972; doi:10.1186/s12870-019-1997-2)
Supplement: Supplementary file 9 — Table S5 Primers for quantitative RT-PCR. (DOCX 16 kb) [file 12870_2019_1997_MOESM9_ESM.docx]

| Gene ID | Primer name | Primer sequence（5’-3’） | Amplicon size/bp | Expression  in RNA-seq | Expression  in qRT-PCR |
| --- | --- | --- | --- | --- | --- |
| c194336 g1 i1 | Ferritin-FP | ACCCTTCACTTCTCCCTTCCCAATTCTTCG | 126 | up | up |
|  | Ferritin-RP | CACGGTGGAGGCGCGGACAGAGA |  |  |  |
| c189274 g1 i1 | ERF 4-FP | TGGGAGAGGCGGAGTCGACGGTGCTAC | 180 | up | up |
|  | ERF 4-RP | CCATGGGGAAGATTCGCGGCTGAGAT |  |  |  |
| c203474 g1 i1 | receptor-like protein kinase-FP | TCCGTGTGCTGCTGCTCTGATCGATGG | 189 | up | up |
|  | receptor-like protein kinase-RP | TGGCGGCGGGAGATGTTTTGGAGTC |  |  |  |
| c205019 g2 i1 | MLO2 -FP | CCAAAGGTTATGACAAATGCGCTGACAAGG | 189 | up | up |
|  | MLO2 -RP | TCCAAAAGATGTGTCCCTCGCAAACCTGAA |  |  |  |
| c207395 g1 i3 | disease resistance protein-FP | TGGCTGAAGGTTGTGTACATGGACGGAA | 176 | up | up |
|  | disease resistance protein-RP | GGCCTTGCGAATTCACCGATCTGGATG |  |  |  |
| c194017 g1 i1 | dehydrin -FP | GGCGGCCGGAGAGGTCACTGATCGT | 187 | up | up |
|  | dehydrin -RP | CCTCTTCTCACCATCTTCACCTTCTTCCTC |  |  |  |
| c201592 g1 i1 | proline dehydrogenase-FP | CCGGCGCAAAAATGGTCATAAAACGTACTT | 195 | down | down |
|  | proline dehydrogenase-RP | GCCCGTCTTTTCTCCTCTATCCCCACATC |  |  |  |
| c178914 g1 i1 | Ribosome protein-FP | CCGGTCTCGCATGGTTTGGGCCTC | 142 | down | down |
|  | Ribosome protein-RP | TGCGGCTGCTCAGATTACCACCATAGTTAG |  |  |  |
| c193030 g1 i3 | NRT1-FP | CTTTGTGGGTGCTGCTGCTGCCTTTGAT | 159 | down | down |
|  | NRT1 -RP | TTTCGGGTTTGGTCTCTGCTTAGCTGCT |  |  |  |
| c175262_g2_i1 | O-acyltransferase WSD1-like-FP | TCAACGTCAAGATGGGGAAACTCA | 155 | up | up |
|  | O-acyltransferase WSD1-like-RP | GGAAAACCAGATATACTCGCGGCAACA |  |  |  |
| c194457_g2_i2 | aquaporin TIP1--FP | CTTCGAGAGAGCCAACGCCTACTACAAA | 270 | up | up |
|  | aquaporin TIP1--RP | GCCTTCTGCTTCTTCCATCGGTGAG |  |  |  |
| c161655_g2_i1 | pre-mRNA-splicing factor CLF1-FP | TCCGTGTGCTGCTGCTCTGATCGATGG | 250 | up | up |
|  | pre-mRNA-splicing factor CLF1-RP | TGGCGGCGGGAGATGTTTTGGAGTC |  |  |  |
| c197830_g3_i1 | potassium transporter -FP | TGAATATGAACAACCCCTGAGTCTCTAC | 174 | up | up |
|  | potassium transporter -RP | GCCGCCAGAGACATCAGACCAGAGA |  |  |  |
| c191638_g3_i8 | senescence-associated carboxylesterase -FP | GGGAGCCAAAAGCCAATAATCTGCAAAC | 240 | up | up |
|  | senescence-associated carboxylesterase -RP | GACGAATCGATGGCTTGAGCTGGAGA |  |  |  |
| c187590_g3_i3 | transcription factor MYC3-FP | CGCCGCGGCCAATAAAAACCAGTAAATA | 201 | up | up |
|  | transcription factor MYC3-RP | CGTCGGGTAGTTGTGTAGAGATGGAGATTG |  |  |  |
| c200550_g2_i1 | EARLY FLOWERING 3 –FP | TGCGGAACAACCTTTATCACTCTCGTCA | 269 | up | up |
|  | EARLY FLOWERING 3-RP | GCCAACAACAACAACAGCCCAATGAG |  |  |  |
